# Characterization of an AGAMOUS-like MADS Box Protein, a Probable Constituent of Flowering and Fruit Ripening Regulatory System in Banana

**DOI:** 10.1371/journal.pone.0044361

**Published:** 2012-09-11

**Authors:** Swarup Roy Choudhury, Sujit Roy, Anish Nag, Sanjay Kumar Singh, Dibyendu N. Sengupta

**Affiliations:** 1 Division of Plant Biology, Bose Institute, Kolkata, West Bengal, India; 2 Department of Chemistry, Bose Institute, Kolkata, West Bengal, India; University of Leeds, United Kingdom

## Abstract

The MADS-box family of genes has been shown to play a significant role in the development of reproductive organs, including dry and fleshy fruits. In this study, the molecular properties of an AGAMOUS like MADS box transcription factor in banana cultivar Giant governor *(Musa sp*, AAA group, subgroup Cavendish) has been elucidated. We have detected a CArG-box sequence binding AGAMOUS MADS-box protein in banana flower and fruit nuclear extracts in DNA-protein interaction assays. The protein fraction in the DNA-protein complex was analyzed by mass spectrometry and using this information we have obtained the full length cDNA of the corresponding protein. The deduced protein sequence showed ∼95% amino acid sequence homology with MA-MADS5, a MADS-box protein described previously from banana. We have characterized the domains of the identified AGAMOUS MADS-box protein involved in DNA binding and homodimer formation *in vitro* using full-length and truncated versions of affinity purified recombinant proteins. Furthermore, in order to gain insight about how DNA bending is achieved by this MADS-box factor, we performed circular permutation and phasing analysis using the wild type recombinant protein. The AGAMOUS MADS-box protein identified in this study has been found to predominantly accumulate in the climacteric fruit pulp and also in female flower ovary. *In vivo* and *in vitro* assays have revealed specific binding of the identified AGAMOUS MADS-box protein to CArG-box sequence in the promoters of major ripening genes in banana fruit. Overall, the expression patterns of this MADS-box protein in banana female flower ovary and during various phases of fruit ripening along with the interaction of the protein to the CArG-box sequence in the promoters of major ripening genes lead to interesting assumption about the possible involvement of this AGAMOUS MADS-box factor in banana fruit ripening and floral reproductive organ development.

## Introduction

The MADS-box genes, which represent a highly conserved gene family of DNA-binding transcription factors, have been identified in a wide range of eukaryotic genomes including insects, amphibian, yeasts, mammals and plants [1]. The MADS box motif has been found as a typical and unique domain for the members of the MADS-box family of transcription factors which binds to a highly conserved DNA motif known as CArG box. The plant type II MADS-domain transcription factors are comprising of an N-terminal conserved sequence called MADS box (MADS: MCM1-AGAMOUS-DEFICIENS-SRF), followed by an I region and a K box, both of which found to be involved in mediating protein-protein interactions, and the C-terminal domain which has been shown to be essential for ternary complex formation and transcription-activating function [2], [3].

In plants, floral organ identity is controlled by diverse families of homeotic transcription factors. The floral homeotic gene *AGAMOUS* (*AG*) is a group C gene and it encodes a MADS box transcription factor [4]. AG interacts with other MADS box proteins to play essential function for the induction of reproductive organ development in *Arabidopsis* [5].

Extensive studies have been made in recent years to understand the function of MADS-box family of genes in the regulation of flower and fruit development [6]. Several MADS-box genes have been shown to regulate fruit development in both climacteric and non-climacteric fruits. In tomato, a typical climacteric fruit, several paralogues of MADS-box genes have been identified, including *TAG1*, *TAGL2*, *TAGL11*, *TAGL12*, *TAGL1*, *TDR4* and *TDR6* and suggested to be involved in fruit development [7]. Mutation complementation and antisense gene expression analyses have demonstrated function of *LeMADS-RIN* in regulation of fruit ripening [8]. Furthermore, in tomato, a *SEPALLATA (SEP)4-like* gene has been shown to be necessary for normal ripening process. In strawberry, silencing of a fruit-related *SEP1/2-like* (*FaMADS9*) gene has been found to inhibit the normal development and ripening in the petal, achene and receptacle tissues, suggesting the key role of *SEP* genes in regulation of ripening in both climacteric and non-climacteric fruits [9]. However, since MADS-box genes have been found to be functional as dimers or heterogeneous multimers, involvement of additional members of MADS-box genes has been indicated in fruit ripening.

MADS-box genes have also been identified from several other fruits and shown to be involved in fruit development during early stages. In apple, six *Md-MADS* genes, classified to the *AP1* clade and one to the *AG* clade, were isolated and found to be expressed during early stages of fruit development [10]. In grapes, MADS-box genes *VuMADS1* and *VuMADS5*, homologous to *AG* and *SHP*, have been demonstrated to be associated with fruit development [11]. Two MADS-box genes, homologous to *TAG1* and *TAGL1*, have been characterized from peach and their functions in fruit development have been suggested [12]. In banana, another model climacteric fruit, six MADS-box genes have been isolated very recently from Grand Nain cultivar of banana and the interaction between ethylene and the expression of these *MA-MADS* box genes have been studied [13].

In this report, we have made an attempt to systematically investigate the role of an AGAMOUS MADS-box transcription factor in regulation of fruit ripening and floral reproductive organ development in banana cultivar Giant governor *(Musa sp*, AAA group, subgroup Cavendish). DNA-protein interaction studies using banana fruit nuclear extract and mass spectrometry analyses have enabled us to identify a CArG-box binding MADS domain transcription factor from banana fruit and floral tissue. We have cloned the full length cDNA of the identified *AGAMOUS MADS-box* gene from banana fruit tissue and examined the expression profile of gene both at transcript and protein levels in various floral tissues of banana and in different parts of banana fruit during ripening. We have also investigated the interaction of the identified MADS-box protein with the putative CArG-box elements in the promoters some of the major ripening genes to further understand the role of this gene in banana fruit ripening.

## Results

### Detection of CArG-box Motif Binding AGAMOUS-MADS Box Protein from Banana Flower and Fruit Nuclear Extract

**Figure 1:**
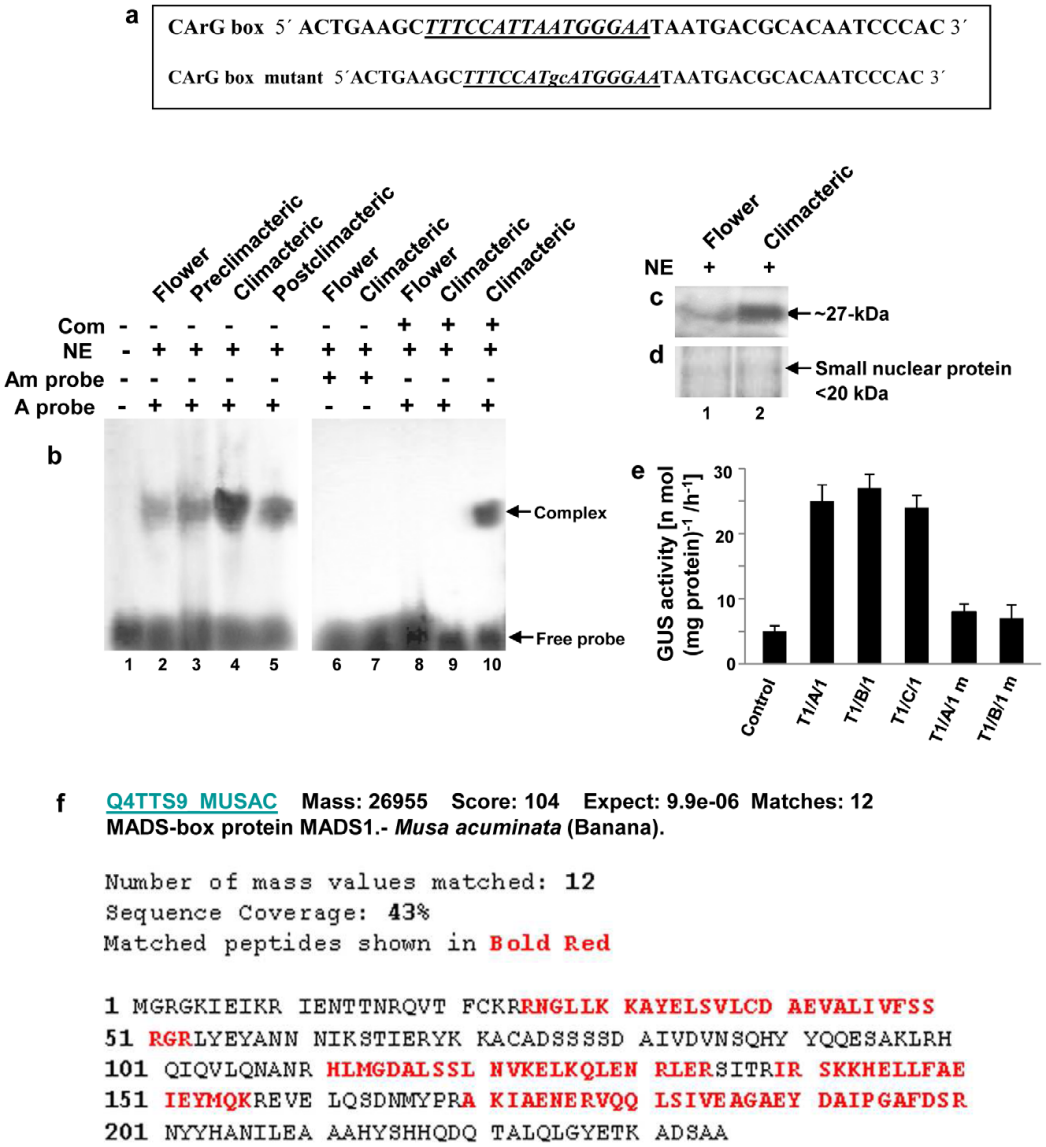
Detection of CArG-box DNA binding protein in banana fruit and flower nuclear extracts. **a** CArG box motif, derived from *Arabidopsis* Agamous MADS box binding site, was used as probe for gel mobility shift assays. The MADS domain consensus binding site (CArG box motif) has been indicated (italics and underlined). DNA fragment containing the modified CArG box motif (highly conserved T and A residues were changed to G and C residues) has been indicated (small letters underlined). **b** Gel mobility shift assay using labeled CArG-box DNA as probe (A probe). Lane 1 contained only radiolabeled CArG-box DNA probe, while 15 µg nuclear protein extract was added in lanes 2–10. Lane 2 to 5 contained nuclear protein extract from banana flower and fruit pulp tissues at the preclimacteric, climacteric and postclimacteric stages of ripening with labeled CArG box motif as probe. Lane 6 and 7 contained nuclear protein extract from banana flower and climacteric fruit pulp with labeled modified CArG box motif as probe (Am probe). In lane 8 and 9, nuclear protein extract from banana flower and climacteric pulp was incubated with labeled CArG box motif in presence of 100-fold excess of unlabeled (non-radioactive) CArG box motif. Lane 10 contained nuclear protein extract from climacteric banana pulp with labeled CArG box motif in presence of 100-fold excess of unlabeled modified CArG box motif. Com-competitor, NE-nuclear extract. **c** South-Western blot analysis with nuclear protein extracts isolated from flower and climacteric banana fruit pulp (lanes 1–2). ∼25 µg of nuclear extract was loaded in each lane. The radiolabeled synthetic oligo containing CArG box motif was used as probe. **d** Equal amounts of small nuclear protein (SNP) from banana flower and climacteric pulp tissues were resolved in 12% SDS-PAGE and has been shown as loading control. **e** Measurements of GUS activity in transgenic tobacco lines carrying trimeric CArG-box motif (3X CArG) or the modified trimeric CArG-box sequence (m) in fusion with *GUS*. GUS activity was detected in the leaves of control and transgenic tobacco lines. The error bars indicate mean values from three independent observations. **f** Identification of 27-kDa CArG-box binding MADS-domain protein by mass spectrometry. Overall sequence coverage of the peptides with the matched protein (Q4TTS9_MUSAC of *Musa acuminata*). Matched peptides shown in red letters. Experiments were repeated three times. Representative images from at least three independent experiments are shown for Figure b–d.

Previously, involvement of floral homeotic gene *AGAMOUS* (*AG*), which encodes a MADS-domain transcription factor, has been identified for the induction of reproductive organ development in *Arabidopsis* [5]. In addition, role of MADS-box genes in regulation of fruit ripening has been demonstrated [8]. Guided by this information, we first carried out gel shift assays to detect the existence of any CArG-box motif binding AGAMOUS-MADS box protein (s) in banana flower and fruit pulp nuclear extract. The *in-vivo* targets for AGAMOUS-MADS box like protein (s) are not well defined in banana. Previous studies have demonstrated the consensus sequence for AGAMOUS MADS-box protein binding site in *Arabidopsis* [14]–[16]. Consensus DNA-binding sites of MADS box proteins have also been identified in additional studies [14]–[21]. Based on this information, initially, we have prepared pool of synthetic oligonucleotides containing various combinations of consensus CArG-box sequences. DNA-binding experiments were then carried out using nuclear extracts prepared from banana floral tissue and fruit pulp with the pool of synthetic oligonucleotides tested one-by-one for protein binding. We have found strong DNA binding activity in the nuclear protein extracts with the CArG-box core consensus sequences containing ‘CCA’ as the first three nucleotides, ‘TGG’ in the last three and either A or T in the central four nucleotides. The AGAMOUS binding site of *Arabidopsis*, reported previously [14]–[16] was closely related to this consensus CArG-box sequence. Therefore, we designed a synthetic olinucleotide that contained the CArG-box motif, similar to *Arabidopsis* AGAMOUS-MADS box protein binding site and used as probe in DNA-protein interaction studies. The sequence which corresponds to the binding site of *Arabidopsis* AGAMOUS-MADS box protein [14]–[16] and used in our study has been shown in Figure 1a. As shown in Figure 1b, a strong DNA binding activity was detected in case of both flower and fruit nuclear extract using the ^32^P-labeled DNA fragment containing the CArG-box motif. The DNA binding activity was significantly induced in climacteric banana pulp (day 8 after harvest) nuclear extract as compared to nuclear extract from flower and pre and postclimacteric (day 0 and day 12 after harvest) fruit respectively (Figure 1b, lane 2, 3, 4 and 5). To further verify this result, we used a modified version of the ^32^P-labeled CArG-box motif containing synthetic DNA fragment which had two base pair substitutions in the CArG-box motif (Figure 1a). No DNA-protein complex was detected using either flower or fruit pulp nuclear extract when this mutated form of CArG-box motif was used as the target (Figure 1b, lane 6 and 7), demonstrating that DNA binding activity was sequence specific. Whereas 100 molar excess of unlabeled CArG box DNA clearly competed out the DNA binding activity (Figure 1b, lane 8 and 9), unlabeled GATA-box (one of the light responsive elements commonly found in the promoters of light regulated genes) DNA (used as negative control) was unable to compete the binding activity even at 100 molar excess ratio to labeled probe (data not shown). Furthermore, 100 molar excess of unlabeled modified CArG-box motif could not able to compete the CArG-box motif binding activity in nuclear extract prepared from banana fruit pulp at the climacteric phase (Figure 1b, lane 10), suggesting that the DNA binding activity was specific to CArG-box motif in banana flower and fruit.

To further study the CArG-box specific DNA binding activity in banana flower and fruit nuclear extracts, we carried out South-Western blot analysis using the similar 5′-end labeled CArG-box motif used in gel shift assays as probe. South Western blotting identified a nuclear protein factor with an approximate molecular mass of 27-kDa in banana flower and climacteric pulp nuclear extract, indicating existence of a CArG-box motif binding protein both in banana flower and fruit tissues (Figure 1c, lanes 1 and 2). The abundance of the 27-kDa *trans*-acting factor was significantly higher in the climacteric fruit as compared to flower and nuclear extracts prepared from pre and postclimacteric banana fruit pulp tissues (data not shown). This observation was consistent with the relative CArG-box DNA binding activities obtained in gel shift assay with nuclear extracts from banana flower and fruit pulp tissues at various stages of ripening.

Our results have revealed CArG-box sequence specific high affinity DNA-protein complex formation in nuclear protein extracts prepared from banana floral and fruit tissue. Therefore, we next tested whether the AGAMOUS MADS-box binding CArG-box sequence, derived from *Arabidopsis* AG binding sequence, is functionally active in driving gene expression in heterologous system. For this, we have generated transgenic tobacco lines carrying tandem repeats of triple AGAMOUS MADS-box binding element (3X CArG) in fusion with *GUS* reporter gene. Approximately 5-fold higher GUS activity was detected in leaves of transgenic tobacco for *3X CArG-GUS* transgene as compared to non-transformed control lines. On the other hand, GUS activity was significantly reduced in transgenic tobacco lines carrying mutated version of the transgene (*3X CArG_m_-GUS*) (Figure 1e). Furthermore, we have detected an increased GUS activity level in the flower and fruit tissues as compared to leaves in *3X CArG-GUS* transgenic tobacco lines (Figure S1). Taken together, these results have indicated that *Arabidopsis* AGAMOUS-MADS box binding CArG element is functionally active in the heterologous systems and retains its tissue specific activity.

### Identification of CArG-box Motif Specific DNA Binding Factor

The results in the above section have indicated existence of CArG-box specific DNA binding activity in banana flower and fruit pulp tissues. Therefore, to identify the *trans*-acting protein factor which specifically binds to the CArG-box motif in banana flower and fruit tissue, we next eluted the protein fractions from the DNA-protein complex obtained using the ^32^P-labeled DNA fragment containing the CArG-box motif with nuclear protein extracts from banana flower and fruit tissues. Since the abundance of the CArG-box motif binding factor was higher in climacteric fruit than other stages of ripening, in case of fruit tissue, we isolated the CArG-box motif binding factor from the DNA-protein complex obtained using climacteric fruit pulp extract. Similarly, to find out whether the similar protein factor binds to CArG-box sequence in flower, protein fractions were also eluted from the DNA-protein complex obtained using nuclear extract from banana flower. Eluted protein fractions were concentrated, desalted and then resolved in 10% SDS-PAGE followed by staining of the gel with silver salt to visualize the protein bands. A single distinct protein band with an approximate molecular mass near 27-kDa was obtained in the elution fractions from both flower and fruit specific DNA-protein complexes (Figure S2a). This observation was also consistent with the results of South-Western blotting using labeled CArG-box motif with nuclear protein extracts from banana flower and fruit at the climacteric phase (Figure 1c).

The CArG-box binding protein was then isolated from the DNA-protein complex of dried EMSA gel and protein identification was performed by MALDI-TOF/MS analysis as described under “Materials and methods”. The resulting spectrum was used to search the matching protein in the NCBI database, using the Mascot search Program. The search yielded a top score of 104 for Q4TTS9_MUSAC, MADS1 [*Musa acuminata*] (protein score greater than 71 are significant; *P*<0.05] for the samples from climacteric fruit pulp extract. Figure S2b illustrates the sequences of peptides obtained by MS/MS analysis for fruit protein sample which showed appreciable sequence identity with banana MADS-box protein MADS1 (AAY53908). The CArG-box binding protein from flower was also identified as MADS1 [*Musa acuminate*] (Q4TTS9_MUSAC) with a top score of 102 (data not shown). The sequence coverage of the peptides for fruit protein sample against the whole sequence of MADS-box protein MADS1 was ∼43% (Figure 1f). The nominal mass of the identified protein was found to be ∼26.95-kDa which was close to the molecular mass of the CArG-box binding nuclear protein factors from banana flower and fruit as identified in South-Western blotting.

### Molecular Cloning, Sequential and Phylogenetic Analysis of the CArG-box Motif Binding Protein Identified from Banana Fruit

We have next used the PCR based approaches, as described under “Materials and methods” to isolate full-length coding sequence of the gene encoding the identified CArG-box motif binding protein in climacteric pulp tissues of banana cultivar Giant governor (Subgroup Cavendish). The coding sequence of the gene appeared to be a full-length cDNA of 732 bp (Accession number HQ730892) and contained an open reading frame (ORF) that encode for a protein of 243 amino acids (predicted molecular mass of ∼27-kDa). The deduced protein sequence of 243 amino acids contains the N-terminal highly conserved MADS domain (1–60 amino acids, domain I), a short intervening I domain (61–74 amino acids, domain II), a keratin like domain (75–183 amino acids, domain III) and a C terminal region (184–243 amino acids, domain IV), detected in CDD [22] and SMART [23] analyses. The identified protein sequence showed ∼95% amino acid sequence identity with banana MADS5 (MA-MADS5, Accession number ACJ64682) and MADS-box protein MADS1 (AAY53908) and ∼26–43% overall average sequence similarity with other fruit specific MADS-domain protein factors from banana including MA-MADS1 (33%, ACJ64679), MA-MADS2 (34%, ACJ64678), MA-MADS3 (43%, ACJ6468), MA-MADS4 (34%, ACJ64681) and MA-MADS6 (26%, ACJ64683) respectively. Based on this information, the AGAMOUS-MADS-box element binding 27-kDa nuclear protein detected in banana fruit and floral tissues has been considered as the MA-MADS5 in banana cultivar Giant governor and the gene encoding the protein has also been designated as *MA-MADS5*. The full-length protein sequence of the identified MA-MADS5 factor showed high sequence similarity with different MADS group of proteins (Table 1, Table S1). Furthermore, the different signature domains of MADS family proteins were also well conserved in MA-MADS5 and showed high level of similarity with other members of MADS family (Table 1).

**Table 1:** Percentage of sequence similarity of agamous like MADS (MA-MADS5) protein with different MADS group of protein.

Protein	MADS domain	K-domain	Full-length protein
MA-MADS5	97	93	95
AtSHP2	88	59	62
AtSTK	91	61	65
AtAG	88	61	65
LIMADS2	88	78	78
DtSTK	90	77	80
AvMADS	96	81	82
AtAGL6	80	40	53
AtSEP4	67	35	38
AtAGL8	66	35	75

It is suggested that MADS box proteins of the same subfamily or group share similar expression patterns and are often involved in regulating similar kind of developmental processes [24]. Therefore, subfamily membership can be indicative for the putative function of a given MADS box gene. To decipher the position and function of AGAMOUS MADS-box binding MA-MADS5 in MADS family, we performed phylogenetic analysis using the neighbor-joining approach (Figure S3, Table S2). As shown in the dendogram in Figure S3a, MA-MADS5 showed highest sequence similarity with the members of the AG-subfamily of MADS box genes (AG clade), suggesting that the identified MADS-box gene belongs to AG-clade of MADS-box gene in banana.

### Characterization of DNA Binding Activity of Various Domains of MA-MADS5

**Figure 2:**
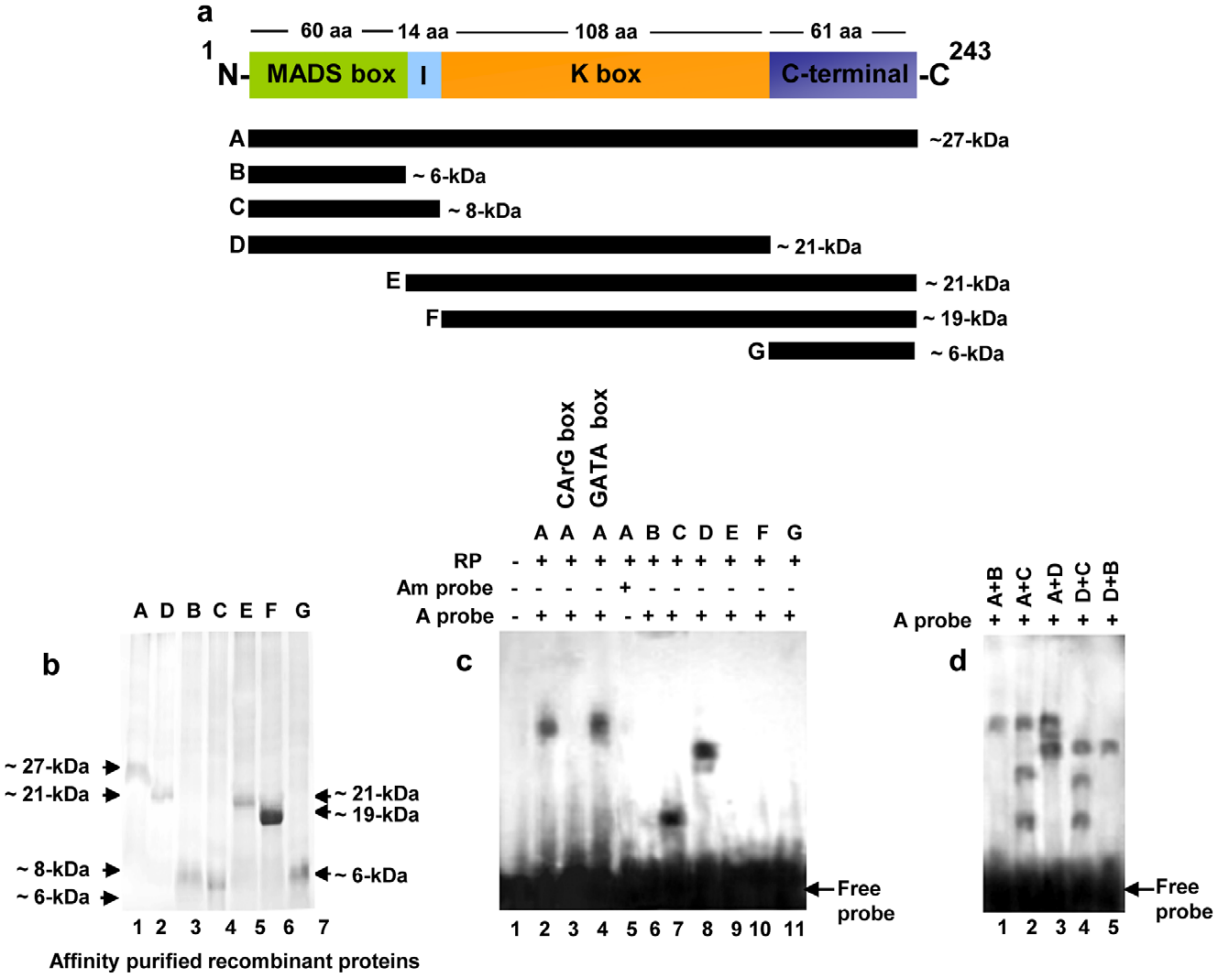
Homodimer formation by MA-MADS5. a Schematic representation of the domain structures of putative MA-MADS5 protein obtained using CDD, SMART and Proscan database analyses. Deleted versions of the wild type full-length protein have been shown. The molecular mass of each protein fragment has been indicated at the right side. **b** Coomassie blue stained gel showing affinity purified fraction of recombinant wild type and different deletion versions of MA-MADS5 proteins. Molecular mass markers are shown on the left and right. **c** Gel mobility shift assay with recombinant wild type and truncated forms of MA-MADS5 proteins using labeled CArG box motif as probe (A probe). No protein was added in lane 1, while lanes 2–4 contained 2 µg purified recombinant wild type protein (A) incubated with labeled CArG-box DNA probe. Lane 3 contained 100-fold excess of unlabeled CArG box motif as competitor. Lane 4 contained 100-fold excess of unlabeled GATA box motif as non-specific competitor. Lane 5 contained labeled modified CArG box motif as probe (Am probe) with wild type recombinant MA-MADS5. Lanes 6 to 11 correspond to gel shift assays using various deletion versions of recombinant MA-MADS5 (B, C, D, E, F and G) with labeled oligo containing the CArG box motif as probe. **d** Detection of dimerization of MA-MADS5 protein by gel mobility shift assay. Formation of heterodimers between wild type and various deletion versions of recombinant MA-MADS5 protein (MIKC with M, MI and MIK or A+B, A+C, A+D respectively) and among the deletion fragments of MA-MADS5 like proteins (MIK with MI and M or D+C, D+B) were detected as relative band shift. Representative images from at least three independent experiments are shown for Figure b–d. M, I, K and C represent the MADS-box, I-region, K box and C-terminal domains of MA-MADS5, respectively.

The MADS-domain proteins generally act as transcription factors and bind to DNA sequence known as CArG-box. Therefore, to understand the structure-function property of the identified MADS-box protein, we next investigated the relative DNA binding activity of different structural domains of MA-MADS5 protein. As shown in Figure 2a, MA-MADS5 has been found to be comprising of an N-terminal highly conserved MADS-box domain, followed by an ‘I’ and K-box domains and a C-terminal domain. To study the DNA binding activity of these structural regions, we generated six deletion fragments from *MA-MADS5* cDNA which corresponds to region ‘B’ (includes only MADS domain), region ‘C’ (MADS and I domains), region ‘D’ (MADS, I and K-box domains), ‘E’ (I, K-box and C-terminal domains), ‘F’ (K-box and C-terminal domains) and ‘G’ (only C-terminal domain) respectively (Figure 2a). The full-length *MA-MADS5* cDNA (shown as region ‘A’ which contains all the four domains) as well as the other deleted versions of the cDNA were individually overexpressed in *E coli* (M15 pREP4 host) cells to generate 6X-His-tagged fusion proteins. The recombinant proteins were purified using Ni^2+^-NTA affinity resin as described under ‘Materials and Methods’ (Figure 2b, Figure S4a and b).

To first test whether MA-MADS5 specifically interacts with the CArG-box motif, we used affinity resin purified recombinant MA-MADS5 protein (Figure 2b) and CArG-box DNA (Figure 1a) as probe in gel shift assay. A high affinity DNA-protein complex was detected along with the free probe as shown in Figure 2c (lane 2). Whereas this DNA binding activity was clearly competed out with 100 molar excess of unlabeled CArG-box DNA (Figure 2c, lane 3), no competition could be detected with 100 molar excess of unlabeled GATA-box DNA (Figure 2c, lanes 4) and no complex was formed in presence of CArG_m_ (mutated version of CArG-box) probe (Figure 2c, lane 5), indicating that the DNA binding activity was specific to CArG-box motif.

To further identify the specific structural region (s) of MA-MADS5 protein associated with the CArG-box-binding activity, we then performed gel shift assays using affinity resin purified various deletion versions of recombinant MA-MADS5 proteins (corresponding to regions ‘B’–‘G’ respectively) and ^32^P-labeled CArG-box DNA as probe. As shown in Figure 2c, distinct low mobility DNA-protein complex was detected with the ‘C’ (includes MADS and I domain) and ‘D’ (includes M, I and K-box) fragments only, while a very weak complex could be detected using ‘B’ fragment (includes only MADS domain) (Figure 2c, lanes 6–8). Conversely, no DNA-protein complex was detected using ‘E’, ‘F’ and ‘G’ fragments of recombinant MA-MADS5 protein (Figure 2c, lanes 9–11). Together, these results clearly suggest that the I-region of MA-MADS5 protein is essential and sufficient for the CArG-box motif binding activity together with MADS-box domain while the MADS-domain region alone is unable to form high affinity complex with CArG-motif.

### MA-MADS5 Forms Homodimers

In *Arabidopsis*, AG (AGAMOUS) has been shown to bind DNA as dimers [18]. In addition, biophysical studies have indicated that dimerized MADS-box domain binds to CArG-box DNA [25]. Therefore, we next studied whether MA-MADS5 shows similar DNA binding features as like AG of *Arabidopsis* and other MADS-box proteins *in vitro*. Dimer formation can be directly detected by the formation of a heterodimer between two proteins of different length. We investigated the possibility of dimer formation by MA-MADS5 *in vitro* using the truncated versions of recombinant MA-MADS5 proteins, including fragments ‘B’, ‘C’ and ‘D’, which showed clear DNA binding activity. However, fragment ‘B’ alone showed very weak CArG-box DNA binding activity (Figure 2c). As shown in Figure 2d, DNA binding assays using labeled CArG-box motif revealed formation of a single high affinity complex when ‘B’ fragment (includes MADS domain) was mixed with either full-length (‘A’) or ‘D’ fragment of MA-MADS5 (Figure 2d, lanes 1 and 5). In contrast, when ‘C’ or ‘D’ was individually mixed with ‘A’ or ‘C’ and ‘D’ were mixed together, distinct and additional DNA-protein complexes with intermediate mobilities in between the mobility of DNA-protein complexes formed individually with either ‘A’, ‘C’ and ‘D’ were detected (Figure 2d, 2–4). The complex with intermediate mobility indicated the possibility of formation of dimers between the truncated versions of recombinant MA-MADS5 protein. Interaction between the various fragments of recombinant MA-MADS5 *in vitro* in DNA binding assays have suggested that MA-MADS5 may bind to DNA as homodimer or heterodimer as other MADS-domain transcription factors also possess the highly conserved MADS-domains and other motifs like I and K-box.

### MA-MADS5 Mediated DNA Bending at the Core CArG-box Sequence

**Figure 3:**
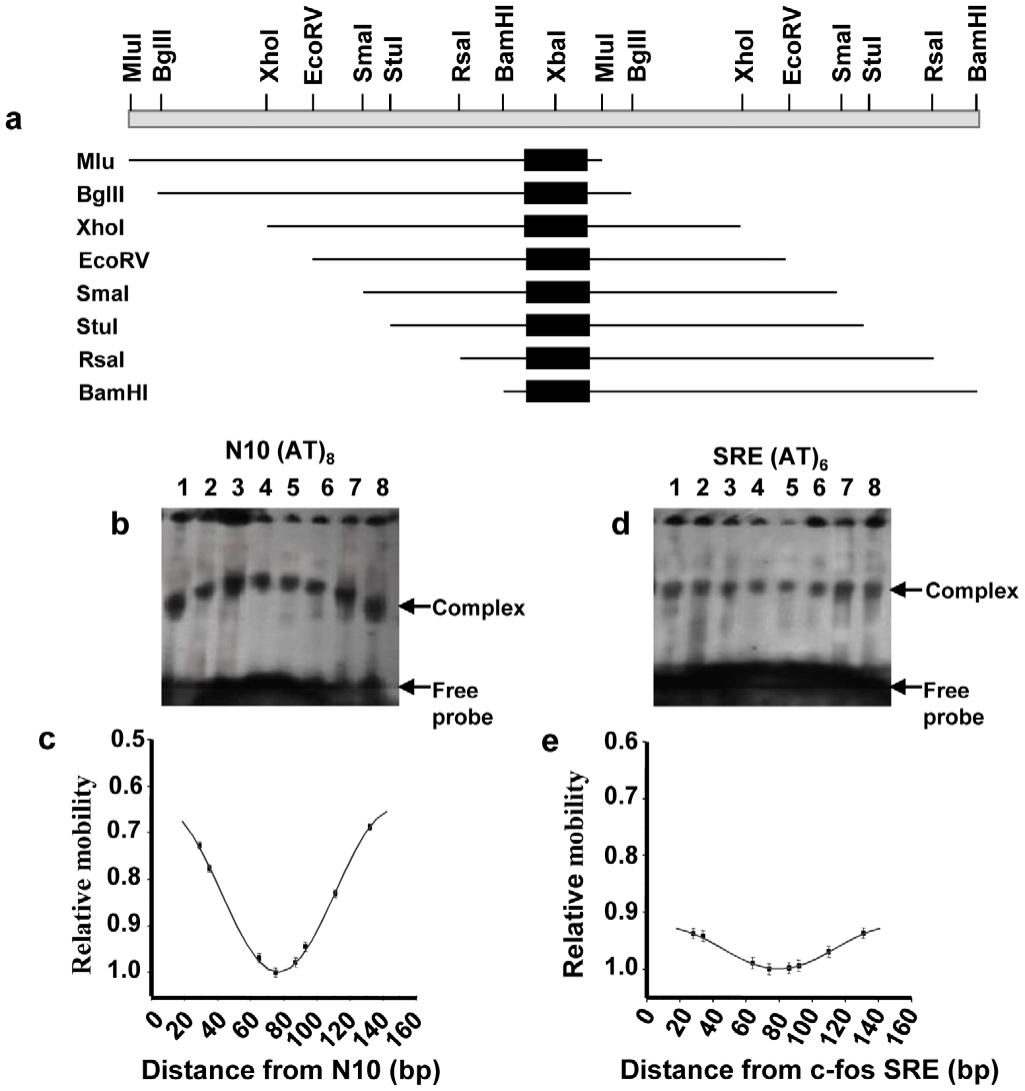
Circular permutation analysis of MA-MADS5 protein using the N10 and c-*fos* SRE sites. **a** Diagrammatic representation of N10 and SRE sites generated by restriction digestion of pAS152 and pAS76 respectively. The location of N10 site and c-*fos* SRE were indicated by filled boxes. Probes were generated by digestion with MluI, BglII, XhoI, EcoRV, SmaI, StuI, RsaI and BamHI, respectively. **b and d** Gel mobility shift assay of MA-MADS5 protein bound to each of the circularly permuted probes containing either the N10 or SRE sites (lanes 1–8). DNA-protein complexes were analyzed on a 6% non-denaturing polyacrylamide gel. **c and e** The data from each circular permutation analysis was shown graphically beneath each set of primary data. The relative mobilities of DNA-protein complexes were normalized for differences in probe mobility and plotted as a function of the position of the center of the N10 or SRE site from the 5′ end of the probe respectively. The points were connected by a curve of the best fit of cosine function. Error bars indicated standard deviations calculated from at least three independent experiments. Representative images from at least three independent experiments have been shown for Figure b and d.

Transcription factor-induced DNA bending is important in determining local promoter architecture and it is thought to be a key determinant of their function. In banana, although number of MADS box transcription factors have been identified in recent years [13], [26], their function in DNA bending have not been studied. Therefore, in order to study the protein induced DNA bending, we next investigated the proficiency of MA-MADS5 to bend DNA by using circular permutation analysis following Sharrocks and Shore [27]. Studies in mammalian and yeast cells have shown that MADS box protein binds to one of two classes of binding sites based on the central consensus motifs, 5′-CC(A/T)_6_GG-3′ (SRE-like) and 5′-CTA(A/T)_4_TAG-3′ (N10-like). Therefore, a panel of CArG-box containing binding site of identical sequence and length on N10-site and SRE were used for gel shift assays (Figure 3a). Among the sites tested, greater extent of DNA bending by MA-MADS5 was detected at the N10 site (5′AAAA**CTATTTATAG**ATCA 3′), while the degree of bending was appreciably lower at the SRE site (5′ATGT**CCATATTAGG**ACAT3′) (Figure 3b, c, d and e). On the other hand, in case MA-MADS5-N10 interaction (DNA binding), saturation (no increase in DNA-protein complex formation even when DNA concentrations were increased further) was achieved much earlier than with SRE, suggesting relatively higher affinity of MA-MADS5 towards N10-site than SRE like element. The relative binding efficiencies of recombinant MA-MADS5 protein towards N10 and SRE probe DNA were analyzed in nonlinear regression plot for determination of BMAX value and dissociation constant (KD) (Text S2, Figure S5). Our results indicated that MA-MADS5 binds to N10-site more efficiently as compared to the SRE-site [reflected by the lower KD values for N10 (4.515) than SRE-sites (10.63) respectively]. Furthermore, as indicated earlier, MA-MADS5 induced DNA bending was higher in case of N10-site than SRE. Therefore, it appears that different intrinsic properties of the protein lead to differences in the recognition of DNA sequences and thus results in differential DNA bending. In fact, we observed several differences within the central core 10 bp regions and also in the flanking nucleotide sequences of N10- and SRE-sites. Based on this information, it can be speculated that MA-MADS5 specifically induces DNA bending depending on the relative affinity towards the target DNA molecule and specificity of recognition sequence.

**Figure 4:**
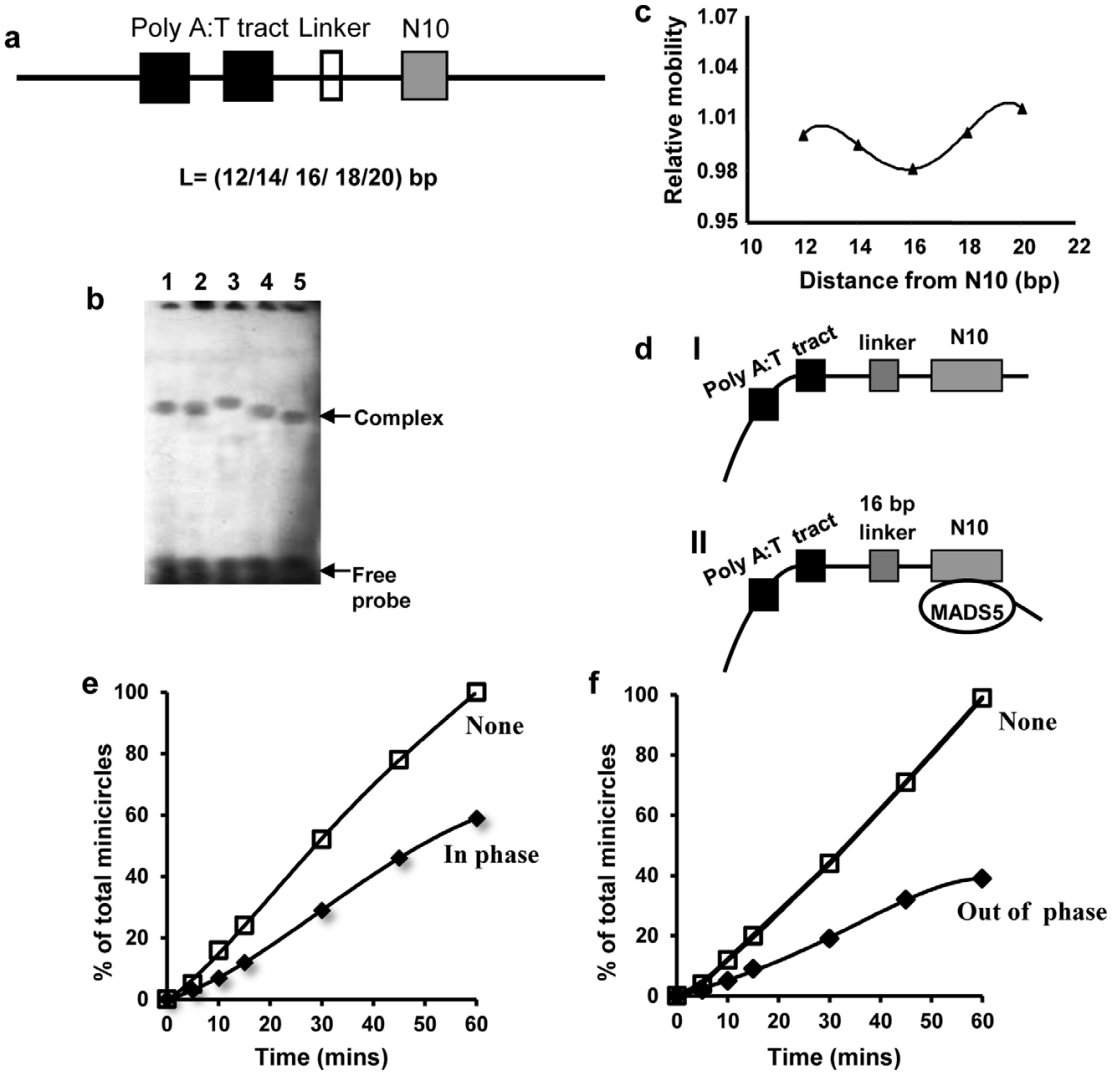
Phasing analysis of MA-MADS5 induced DNA bending in complexes with N10-site. a Diagrammatic representation of phasing probes. The sequence giving rise to the intrinsic bend (filled box) is composed of two helically phased hexameric [A:T] tracts. The linker lengths (open box) varies between 12 to 20 bp, giving rise to spacing of 51 and 59 bp respectively between the centre of the first hexameric [A:T] tract and the centre of the CArG box (N10-site) (grey box). **b** Bacterially expressed recombinant MA-MADS5 protein was incubated with each of the phasing probe and analyzed by electrophoresis through a 6% non-denaturing polyacrylamide gel. The mobilities of free probes have been indicated. The different linkers in each probe resulted in spacer lengths of 51 bp (lane 1), 53 bp (lane 2), 55 bp (lane 3), 57 bp (lane 4) and 59 bp (lane 5) respectively between the centre of the first hexameric poly [A:T] tract and the centre of the CArG box (N10-site). **c** The relative motilities of DNA-protein complexes were normalized for differences in probe mobilities and plotted as a function of the distance between the centre of the N10 from the intrinsic DNA bend. **d** Diagrammatic representation of the structure of the complexes containing an intrinsic DNA bend (I), MA-MADS5 induced bend on N10-site and ‘in-phase’ (II) with the intrinsic DNA bend. **e–f** Ligase mediated circularization analysis of MA-MADS5 protein on the N10 site. Assays were carried out on sites containing the linker of 16 and 20 bp in which the centre of the N10 site was ‘in-phase’ (e) or ‘out-of-phase’ (f) with respect to the centre of a first hexameric poly [A:T] tract. Minicircle formation after incubation with DNA ligase for the indicated time points (0 to 60 min) has been graphically represented. The circularization reactions were carried out in the absence or presence of MA-MADS5 protein. Experiments were repeated three times.

Circular permutation analysis alone is not sufficient to confirm whether protein binding induces DNA bending or only increases flexibility of DNA. Therefore, to further investigate DNA bending by MA-MADS5, we first performed phasing analysis to verify the DNA bending by the protein within the DNA-protein complexes and to determine the ability of protein induced bend to enhance and counteract an intrinsic bend. For phasing analysis, we used N10-site since MA-MADS5 induced DNA bending was comparatively higher in this site than SRE. DNA fragments were constructed in which N10 site was located at different locations downstream from six poly [A:T] tract as described under “Materials and methods” (Figure 4a). This resulted in a series of fragments in which the intrinsic bend, resulting from poly [A:T], was phased throughout one helical turn from the N10. When the DNA bending at the N10 site was in the same orientation as the intrinsic bend, cooperativity could be observed, resulting in slow moving complex (Figure 4b, lane 3). Here transcription factor binding did not neutralize the effect of intrinsic bend on DNA. Conversely, fast migrating complex was formed, when the protein induced bend on N10 and intrinsic bend were in opposite direction, resulting in neutralization of the effect of the intrinsic bend (Figure 4b, lane 5). The ratio of relative mobility of DNA-protein complex vs relative mobility of free DNA was plotted as a function of the distance between the intrinsic and protein induced bend centres (Figure 4c). The resulting curve indicated that maximum cooperatively could be observed, when the two bend centres were separated by 55 bp (center of the N10 site to center of first A:T tract) (linker 16 bp) (Figure 4b, c and d II). This corresponded to 5.2 helical turns (10.5 bp/helical turn), demonstrating that the intrinsic and protein induced bends were ‘in- phase’ (Figure 4c and dII).

Based on the above results we next carried out ligase-mediated circularization assay to further study the DNA-bending efficiency of MA-MADS5. In this assay, the intra-molecular ligation rates of probes containing the N10 sites either in or ‘out of phase’ (slower or faster moving complex) with an intrinsic DNA bend were analyzed in the presence of DNA bend inducing protein, MA-MADS5. Gel mobility shift assay during phasing analysis has indicated that two bend centers when separated by 55 bp or 59 bp, caused the N10 binding site to be in or out of phase with poly A:T tract. A higher ligation rate would be expected when probe is ‘in-phase’ due to increased bending and closer proximity of two ends. On the other hand, lower ligation rate results in case of ‘out of phase’ probe. We studied ligation mediated circularization in presence of purified recombinant MA-MADS5 protein (Figure 4e and f). The ligase-mediated circularization rate was found to be relatively higher when probe was ‘in phase’ (Figure 4e) than ‘out of phase’ probe (Figure 4f). The percentage of total minicircles shown on the Y axis refers that without any protein there is formation of 100% minicircles (minicircle formation detected without any added protein was considered as 100%). In comparison to free DNA, MA-MADS5 appeared to inhibit ligase mediated circularization. These results have indicated that MA-MADS5 induced DNA bending affect the circularization rate differently *in vitro* in case of ‘in-phase’ and ‘out of phase’ condition of the probes.

### MA-MADS5 Protein Accumulates in Pulp Tissue at the Climacteric Phase during Banana Fruit Ripening

Role of MADS-domain protein in regulation of fruit ripening has been extensively studied in tomato [21], [28]. A more recent study has demonstrated that several MA-MADS-box genes may participate in the ripening in banana [13]. Therefore, to understand the biological function of *MA-MADS5* in banana fruit ripening, we next examined the expression pattern of *MA-MADS5* transcripts in various tissues of banana (Giant Governor, subgroup Cavendish) (Text S2). Higher message levels of *MA-MADS5* were detected in female flower ovary and fruit pulp at the climacteric peak (Figure S6). Furthermore, in pulp, the abundance of *MA-MADS5* mRNA increased in parallel with the ripening days and maximum expression was detected at climacteric phase. In peel tissue, *MA-MADS5* message level remained relatively low throughout the ripening days (Figure S6). *MA-MADS5* expression was found to be induced by ethylene only in pulp (Figure S6), suggesting that *MA-MADS5* expression is probably regulated by ethylene induced tissue specific transcription factor (s).

**Figure 5:**
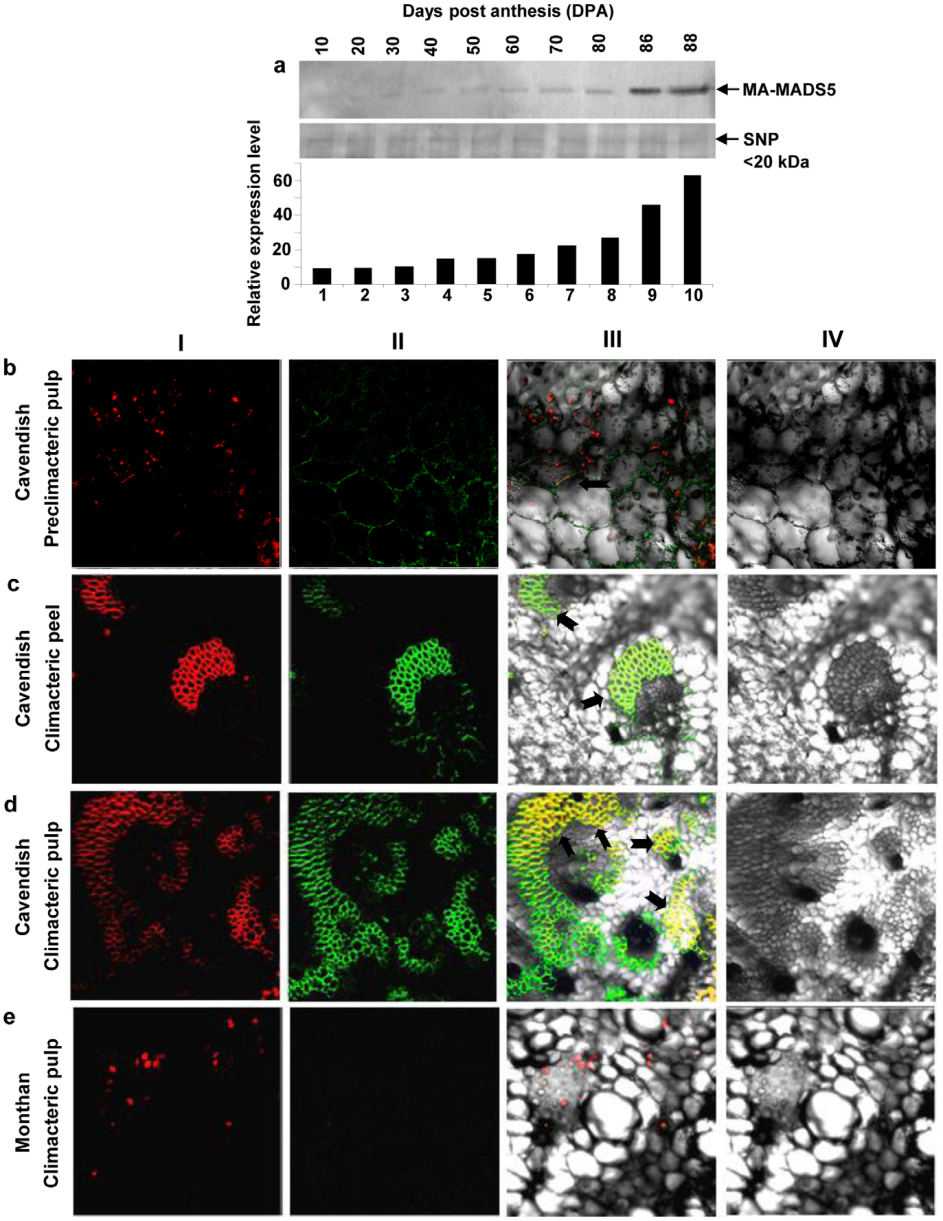
Immunoblotting and immunolocalization of MA-MADS5 protein in fruit tissues of banana. **a** Changes of protein level of MA-MADS5 in developing fruit tissues were analyzed by immunoblotting using affinity purified anti-MA-MADS5 polyclonal antibody (1∶1000 dilution) (upper panel). Equal amounts of small nuclear protein (SNP) was loaded in each lane and shown as loading control (middle panel). Quantification of the data in (**a**) by densitometry (Bio-Rad Immage Densitometer G700) (lower panel). Representative images from at least three independent experiments are shown for Figure a. **b–d** *In situ* localization of MA-MADS5 protein using FITC-coupled affinity purified anti-MA-MADS5 IgG in the preclimacteric pulp (0 DAH), climacteric peel and climacteric (88 DAH) pulp tissues of Cavendish and in climacteric pulp tissues of Monthan. Nuclei were stained with DAPI (red fluorescence) (First row, I). MA-MADS5 was detected using anti-MA-MADS5 IgG coupled to FITC (green images) (Second row, II). Nuclear localization of MA-MADS5 (yellow fluorescence) has been shown in the merged pictures of I and II of b to e respectively (third row, III). Fourth rows (IV) of b–e indicate difference interference contrast pictures.

Based on the observation of increased expression of *MA-MADS5* transcripts in ripening banana fruit, we next studied the accumulation levels of MA-MADS5 protein in banana fruit pulp during *ex-planta* ripening at different days after anthesis using affinity purified anti-MA-MADS5 polyclonal antibody (Text S2, Figure S7) We have observed an increased accumulation of MA-MADS5 protein along with the ripening days at different days post anthesis (DPA) (Figure 5a, lanes 1–10). Maximum expression of the protein was detected at 88 DPA which corresponded to the climacteric phase of ripening in this cultivar of banana [29].

To further substantiate this observation, we next examined changes in sub-cellular localization of this protein in peel and pulp tissues of banana fruit at the preclimacteric (unripe green fruit at 80 DPA or day 0 after harvest) and climacteric (88 DPA or 8 DAH) stages of ripening in Cavendish banana. We were not able to detect any signal of MA-MADS5 in peel tissue at the preclimacteric stage (data not shown) while very weak and mainly cytosolic localization signal of the protein was detected in pulp tissue at similar stage of ripening (Figure 5b). Interestingly, in peel and pulp tissues of climacteric fruit, MA-MADS5 localization was mainly associated with the vascular tissue (indicated by black arrow heads). In climacteric peel tissue, MA-MADS5 signal was mainly cytosolic, while in pulp, in addition to cytosolic staining, notable increase in nuclear localization signal of the protein was detected (yellow fluorescence indicated by black arrow heads) (Figure 5c and d). We have also confirmed the increased level of this MADS-box protein in climacteric pulp nuclear protein fraction as compared to cytosolic fraction in Western blotting (data not shown). In banana, due to starch deposition in the tissues, the nuclei are pushed towards the cellular periphery and thus nuclear staining may appear at the peripheral region.

Previously we have characterized ripening behaviour in banana cultivar Monthan (commonly known as ‘cooking’ banana) which showed significantly delayed ripening with extremely reduced expression levels of major ripening genes and ethylene production [29]. We used this cultivar as negative control to compare the sub-cellular localization signal of MA-MADS5 protein with Cavendish cultivar. We could not able to detect any transcript or protein of *MA-MADS5* in Monthan fruit (data not shown). On the other hand, in contrast to climacteric fruit pulp tissue of Cavendish (Figure 5d), FITC-tagged antibody was unable to detect any signal of MA-MADS5 protein in climacteric pulp tissue of Monthan (Figure 5e), even in mature ripe fruit (15 DAH) (not shown). However, it was necessary to test whether the anti-MA-MADS5 antibody also works in Monthan cultivar. For this, we performed Western blot analyses using equal amounts of protein extracts (40 µg) from various floral tissues including bracts, tepals, stamens, stigma, style and ovary tissues from Monthan flower. In contrast to fruit tissues, we have detected the expression of MA-MADS5 protein in the stamens, style, stigma and ovary tissues using the affinity purified anti-MA-MADS5 polyclonal antibody. However, the expression level was low compared to the similar tissues in Cavendish (data not shown). These results demonstrated that the immunolocalization assay of MA-MADS5 protein for Monthan was negative in fruit tissue due to lack of expression of the target protein in fruit. Taken together, the accumulation profile and sub-cellular localization pattern of MA-MADS5 in ripening banana fruit may suggest possible role of the protein in fruit ripening.

### MA-MADS5 Protein Accumulation is Associated with Banana Fruit Ripening

**Figure 6:**
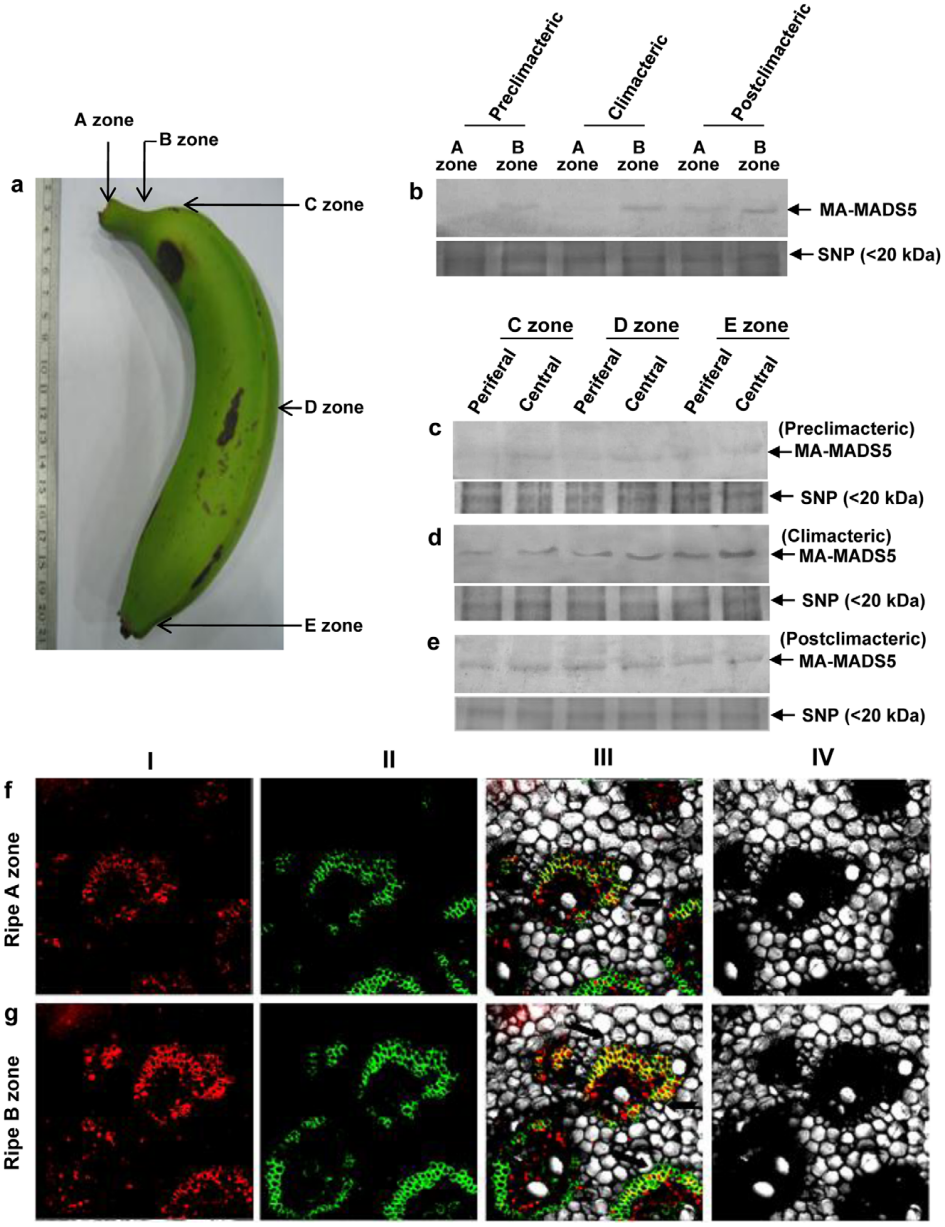
Analysis of expression of MA-MADS5 protein in various regions of banana fruit tissue. a Various regions of banana fruit from the cultivar Giant governor (*Musa sp*, AAA group, subgroup Cavendish) have been indicated (zones A–E). **b** Detection of MA-MADS5 protein in A and B zones of preclimacteric (0 DAH), climacteric (88 DAH) and postclimacteric (92 DAH) banana fruit by immunoblotting using anti-MA-MADS5 polyclonal antibody (1∶1000 dilution) (upper panel). Equal amounts of small nuclear protein was loaded in each lane and shown as loading control (lower panel). **c–e** Immunodetection of MA-MADS5 protein levels in C, D and E zones (including both peripheral and central parts) of preclimacteric, climacteric and postclimacteric fruit pulp tissues of banana (upper panel). Equal amounts of small nuclear protein was loaded in each lane and shown as loading control (lower panels of c, d and e respectively). Representative images from at least three independent experiments are shown for Figure b–e. **f and g** Immunolocalization of MA-MADS5 protein in A and B zones in postclimacteric banana pulp tissues (92 DAH). The legends of the panels I–IV are similar as described in Figure.5 b–e.

To further understand the functional relevance of MA-MADS5 in banana fruit ripening, we next investigated the accumulation pattern of the protein in different regions of banana fruit at various stages of ripening including preclimacteric (80 DPA), climacteric (88 DPA) and post climacteric (92 DPA) phases respectively. For this, we have sub-divided ripening banana fruit into five different regions from the upper pedicel region to the bottom part of the fruit (zone A to E) (Figure 6a). Regions A and B correspond to the upper pedicel and its adjacent lower ‘finger drop’ zone of fruit respectively. Regions C, D and E correspond to the fruit parts just below the ‘drop zone’, central region and the bottom part of the fruit. MA-MADS5 protein level in different parts of banana fruit at various stages of ripening was detected by immunoblotting using banana fruit pulp nuclear protein extracts prepared from the indicated regions (Figure 6a). As shown in Figure 6b, near the upper pedicel region (zone ‘A’) accumulation level of MA-MADS5 was negligible at the preclimacteric and climacteric stages of ripening while increased marginally in postclimacteric fruit. Conversely, in ‘finger drop’ region (‘B’ zone), MA-MADS5 protein level was increased steadily along with the ripening stages and reached its maximum level at postclimacteric stage (Figure 6b).

We have further sub-divided each of zones C, D and E into peripheral and central regions respectively. At the preclimacteric stage, MA-MADS5 protein level was considerably low in both the peripheral and central regions of zones ‘C’, ‘D’ and ‘E’ respectively with no significant difference among the three zones (Figure 6c). Interestingly, in climacteric pulp tissue, MA-MADS5 accumulation level increased appreciably in the central parts of zones C–E and maximum expression of the protein was detected in the central region of zone E of ripening banana fruit (Figure 6d). The abundance level of the protein decreased notably in both peripheral and central regions of zone ‘C’, ‘D’ and ‘E’ respectively at the postclimacteric stage of ripening (Figure 6e). Furthermore, only little, if any, difference in the accumulation level of MA-MADS5 could be detected between the peripheral and central parts of fruit in the ‘C’, ‘D’ and ‘E’ zones in postclimacteric fruit. Taken together, these results indicate that MA-MADS5 accumulate in most of the regions of ripening banana fruit particularly at the climacteric stage. However, increased abundance of the protein as well as nuclear localization in the finger drop region (‘B’ region) at the postclimacteric stages (ripe fruit at 92 DPA or 12 DAH) was interesting (Figure 6f and g), since this region is associated with fruit abscission at the later stages of *ex-planta* ripening in banana as in other climacteric fruits.

### Study of *in vivo* Binding of MA-MADS5 to the Promoters of Genes Involved in Ethylene Biosynthesis and Fruit Ripening in Banana

**Figure 7:**
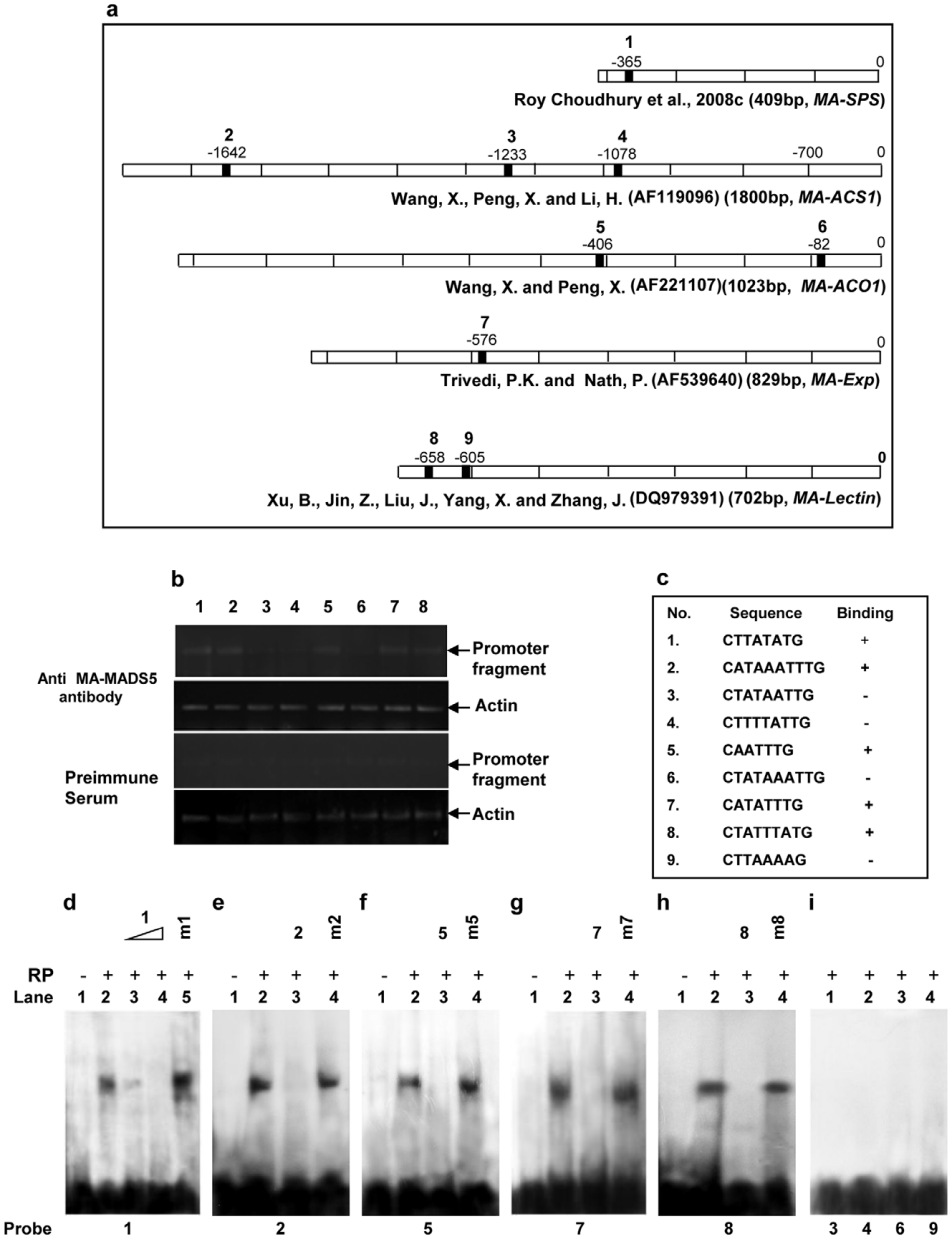
Binding of MA-MADS5 to CArG-box elements in the promoters of different ripening genes in banana. a Analysis of promoter fragments of different ripening specific genes (*MA-SPS*, *MA-ACS1*, *MA-ACO1*, *MA-EXP*, *MA-LEC*) from banana showing the presence of CArG-box elements in different regions of promoter. CArG-box motifs identified in the promoters of different ripening genes have been serially numbered as 1–9 respectively. **b** ChIP PCR assay for the *cis*-elements of *MA-SPS*, *MA-ACS1*, *MA-ACO1*, *MA-EXP*, *MA-Lec*. Chromatin was prepared from climacteric banana fruit, and was immunoprecipitated using anti-MA-MADS5 polyclonal antibody or pre-immune serum, respectively. PCR reaction was performed using primers amplifying the CArG-box motif region of the promoters of the indicated ripening specific genes. Actin was used as control. Representative image from at least three independent experiments are shown for Figure b. **c** DNA sequences of the CArG-boxes within the promoter of different ripening specific genes of banana and the relative binding ability of recombinant MA-MADS5 to these sequences have been indicated by + and – symbols respectively. **d** Gel mobility shift assay using 5′-end labeled synthetic oligonucleotide containing CArG-box motif 1 as probe. No protein extract was added in lane 1, while 2 µg recombinant MA-MADS5 protein was added in lanes 2–5. 50 and 100-molar excess of unlabeled CArG-box motif 1 was added in lanes 3 and 4 as competitor. Lane 5 contained 100 molar excess of unlabeled corresponding mutant version of CArG-box motif 1 (m1) as competitor. **e–h** Gel mobility shift assays using 5′-end labeled synthetic oligonucleotide containing CArG-box motif 2, 5, 7 and 8 respectively as probe. No protein extract was added in lane 1, while 2 µg recombinant protein was added in lanes 2–4. 100-molar excess of unlabeled respective CArG-box motif and unlabeled corresponding mutant version of CArG-box motifs of 2, 5, 7 and 8, respectively (m2, m5, m7, m8) were added in lanes 3 and 4 respectively. **i.** Gel shift assay using 5′-end labeled synthetic oligonucleotide containing CArG-box motifs 3 (lane 1), 4 (lane 2), 6 (lane 3) and 9 (lane 4) as probe. 2 µg recombinant proteins were added in lanes 1–4. Representative images from three independent trials have been shown for Figures d–i. RP-recombinant proteins; Com-competitor.

Our results indicate that MA-MADS5 specifically binds to CArG-box motif and may be involved in banana fruit ripening as indicated by the increased accumulation level of this protein in ripening banana fruit. Based on these results, we were next interested to study whether MA-MADS5 protein binds to the promoter of the major ripening genes in banana. To investigate this, we searched for the existence of putative CArG-box sequences in the promoter regions of major ripening genes in banana like *MA-SPS* (*Sucrose phosphate synthase*), *MA-ACS1* (*1-Amino cyclopropane 1-carboxylic acid synthase 1*), *MA-ACO1* (1*-Amino cyclopropane 1-carboxylic acid oxidase 1*), *MA-Exp* (*Expansin)* and *MA-Lec* (*Lectin*) respectively [30]–[34]. To detect potential MA-MADS5 binding sequences, a possible CArG-box motif [C(C/T)(A/T)6(A/G)G] was searched against the promoters of these genes (∼400 bp to 2 kb). Previously we have characterized the *SPS* gene promoter and identified the transcription start site of the gene [34] while for other genes only promoters were characterized and reported [30]–[34]. The CArG-box motif includes three groups of CArG-box sequences: SRF-like [canonical CArG-box, C(A/T)_6_G], N10 (MEF2)-like [CTA(A/T)_4_TAG], and intermediate [C(A/T)_6_AG]. Previously besides the typical CArG-box motif, some atypical CArG-box motifs were analyzed in tomato for RIN protein [35]. Two atypical CArG-box sequences, CATTTATATG and CAATTTAAAG (here the underlines indicate atypical bases) were detected in the promoter of *LeEXP1* and three atypical CArG-box sequences of CAAATATAAG, CAATTTTAAG and CTAGTTAAAG (underlines indicate atypical bases in contrast to the usual bases of typical CArG-box motif -[C(C/T)(A/T)6(A/G)G]) were detected in the promoter of *LeACS4* [35]. We have identified 9 (typical and atypical) CArG-box motifs from these five genes and then analyzed the sequences using ChIP and gel mobility shift assays. We have detected putative CArG-box sites in the promoter regions of the selected ripening genes (CArG-box sequences were numbered serially as type 1–9 respectively) (Figure 7a). We first carried out chromatin immunoprecipitation (ChIP) assay to examine *in vivo* binding of MA-MADS5 protein to these putative CArG box sequences detected in the promoters of banana ripening genes. Chromatin was prepared from climacteric fruit (88 DPA) and was then immunoprecipitated with the anti-MA-MADS5 antibody. Primer pairs specific to sequences flanking the selected sites were designed (Table S3B). PCR was performed using primers specific to the target site of MA-MADS5. The amplified signal in type 1, 2, 5, 7 and 8 CArG sequences in the ChIP DNA from climacteric fruit was significantly stronger as compared to the pre-immune serum ChIP DNA, suggesting enrichment of those fragments by ChIP (Figure 7b). In contrast, no significant enrichment was detected in the same assays for ChIP DNA from type 3, 4 and 6 CArG sequences. The results were confirmed with the chromatin independently prepared from five climacteric fruits. No significant enrichment was detected, when chromatin independently prepared from preclimacteric fruits (data not shown).

To further validate the above results, we carried out gel shift assays using the 5′-end labeled synthetic promoter fragment carrying each of the specific type CArG-box motif sequence (indicated by the numbers 1–9 as detected in the promoters of the ripening genes) (Table S3B). Whereas strong DNA binding activity was detected with labeled synthetic promoters carrying putative CArG-box motif indicated as type 1, 2, 5, 7 and 8 respectively, no DNA binding activity was detected with type 3, 4, 6 and 9 CArG-box sequences (indicated by +/− symbols) (Figure 7d–i). Competitive gel shift assays in presence of 100 molar excess of unlabeled and mutated or unlabeled and non-mutated form of each of type 1, 2, 5, 7 and 8 CArG-box like motif sequences indicated specificity of the DNA binding activities for each of the respective type of CArG-box like sequences (Figure 7d–h). Furthermore, similar DNA binding activity was obtained with type 1, 2, 5, 7 and 8 CArG-box sequences (Figure S8a–c) but not with type 3, 4, 6 and 9 CArG-box like motifs (Figure S8d) when banana climacteric pulp nuclear extract was used in place of recombinant MA-MADS5. Taken together, these results indicate that MA-MADS5 protein binds specifically to the CArG-box like motifs found in the promoters of the genes which are up regulated during banana fruit ripening.

**Figure 8:**
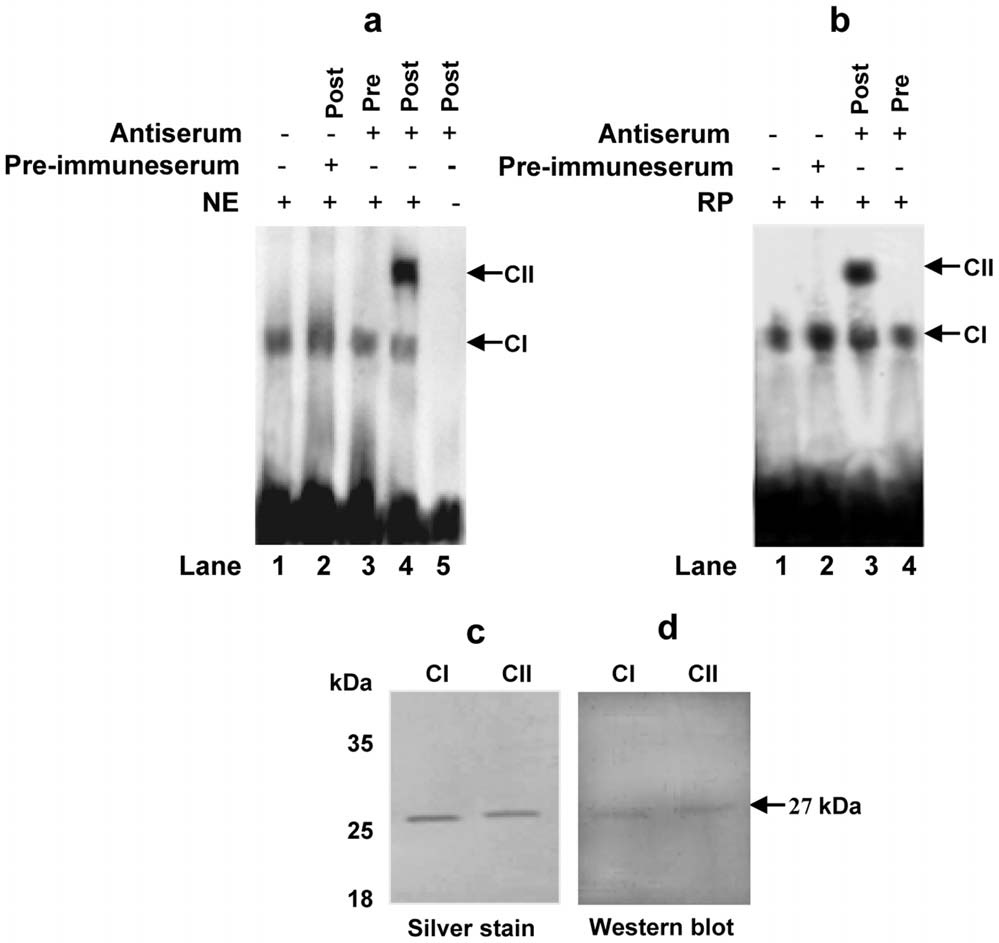
Detection of MA-MADS5 protein in DNA-protein complex involving CArG-box DNA. **a** Gel shift assay using 22-bp 5′-end labeled synthetic oligonucleotide containing CArG-box motif 1 (present in the promoter of banana *SPS* gene) as probe. 15 µg postclimacteric fruit pulp nuclear protein extract was loaded in lanes 1–4. 2 µg preimmune serum was added in lane 2 (post incubation). 2 µg purified anti-MA-MADS5 polyclonal antibody was added in lanes 3 and 4 before (pre) and after (post) the addition of probe to the protein extract. No protein extract was added in lane 5. Formation of shifted and ‘super shifted’ complexes has been indicated as CI and CII. **b** Gel shift assay using 22-bp 5′-end labeled synthetic oligonucleotide containing CArG-box motif 1 (present in the promoter of banana *SPS* gene) as probe. 2 µg purified recombinant MA-MADS5 protein was loaded in lanes 1–4. 2 µg preimmune serum was added in lane 2. 2 µg purified anti-MA-MADS5 polyclonal antibody was added in lanes 3 and 4 after (post) and before (pre) the addition of probe. **c** The DNA-protein complex (CI) from lane 1 (a) and ‘supershift complex’ (CII) from lane 4 (a) were extracted from dried gel and analyzed by silver stain. **d** Western blot analysis of the protein samples eluted from DNA-protein complex (CI) from lane 1 (a) and supershift complex (CII) from lane 4 (a) were performed using anti-MA-MADS5 polyclonal antibody (1∶1000 dilution). Representative images from at least three independent experiments are shown for Figures a–d. Pre-preincubation; Post-postincubation.

The presence of MA-MADS5 protein in CArG-box DNA-protein complex was validated by ‘super shift assay’. We used labeled oligo containing type 1 CArG-box sequence (detected in promoter of *MA-SPS* with positive interaction to MA-MADS5) as probe with climacteric fruit nuclear extract. During gel shift assay, affinity purified anti-MA-MADS5 antibody was added before or after incubation of the labeled probe with the nuclear extract. ‘Super shift’ or complex II was clearly detected for post incubation reaction (Figure 8a). Similar result was obtained when affinity purified recombinant MA-MADS5 was used (Figure 8b), suggesting the presence of MA-MADS5 protein factor in the CArG-box DNA: nuclear protein complex. Rabbit pre-immune serum was used as control (Figure 8a and b). Immunoblot analysis using anti-MA-MADS5 antibody with protein samples eluted directly from the ‘super shifted’ complex of dried EMSA gel (Fig. 8c) have also specifically recognized the 27-kDa protein band of MA-MADS5 (Fig. 8d).

Our results have indicated that MA-MADS5 protein binds specifically to the CArG-box sequence in the promoter of ripening genes in banana as in case of *SPS* gene promoter (Figure 7d). We have next investigated whether the CArG-box sequence present in ripening related gene promoter is functionally active in regulating gene expression. To study this, we have generated transgenic tobacco plants carrying trimeric CArG-box sequences (3X CArG) derived from *MA*-*SPS* gene promoter. We have found that the trimeric CArG-box like motif (MADS-box binding element) of *MA*-*SPS* gene promoter was functionally active in controlling tissue specific gene expression in tobacco (Text S2, Figure S9). Overall, these results have indicated that MA-MADS5 forms complex with the CArG-box sequence in the promoters of ripening genes and the CArG-box sequence detected in ripening gene promoter, as detected in *MA-SPS*, is functional in regulating gene expression.

### MA-MADS5 Protein Accumulation Pattern Correlates with the Transcript Expression Profiles of Major Ripening Genes during Banana Fruit Ripening

**Figure 9:**
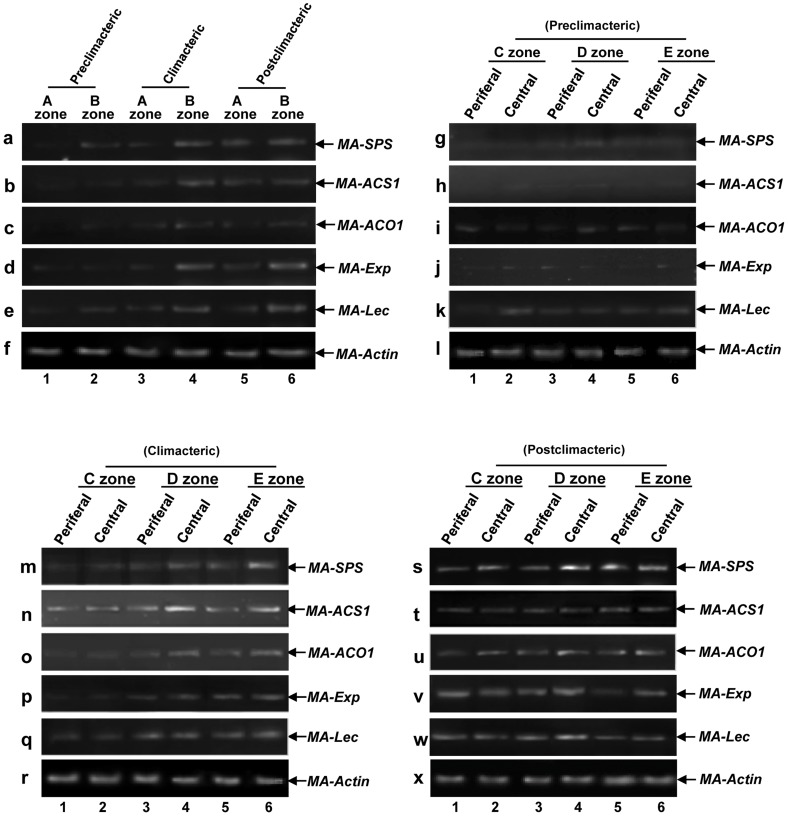
Analysis of expression patterns of major ripening genes in different zones of banana fruit. **a–e** Semi-quantitative RT-PCR analysis was carried to detect the changes of transcript level of *MA-SPS, MA-ACS1*, *MA-ACO1*, *MA-EXP* and *MA-LEC* in A and B zones of preclimacteric (lanes 1 and 2), climacteric (lanes 3 and 4) and postclimacteric (lanes 5 and 6) banana fruit. **g–k** Detection of transcript levels of the selected ripening genes from peripheral and central regions of C, D and E zones of preclimacteric (0 DAH) banana fruit pulp. **m–q** Transcript abundance of the indicated ripening genes in the peripheral and central regions of C, D and E zones of climacteric (88 DAH) banana fruit pulp. **s–w** Changes in transcript levels of the ripening genes in peripheral and central regions of C, D and E zones of postclimacteric (92 DAH) banana fruit pulp. Figures f, l, r and x represent relative message levels of *MA-Actin* measured under the corresponding conditions as internal control. Representative images from at least three independent experiments are shown.

Previously, expression of major ripening genes like *MA-SPS, MA-ACS1*, *MA-ACO1*, *MA-Exp* and *MA-Lec* have been characterized during banana fruit ripening and key roles of these genes in regulation of banana fruit ripening have already been demonstrated [30]–[34], [36]. Based on our observation of accumulation profile of MA-MADS5 in various regions of banana fruit during different ripening stages, we further tried to understand the role of MA-MADS5 in banana fruit ripening in relation to the expression of the major ripening genes. For this, we next investigated whether MA-MADS5 protein expression pattern correlates with transcript expression levels of the major ripening genes during banana fruit ripening. To address this issue, we carried out semi-quantitative reverse transcription PCR (Table S3C) to examine the changes in the endogenous message levels of the above indicated ripening genes in various zones (‘A’ to ‘E’) of ripening banana fruit at the preclimacteric, climacteric and postclimacteric stages of ripening (Figure 9). In general, transcript abundance of the ripening genes were relatively low in ‘A’ zone, which corresponds to the upper pedicel region of fruit, in both preclimacteric and climacteric phases of ripening and then increased marginally at postclimacteric stage (Figure 9a–e, lanes 1, 3 and 5). In the B zone, which corresponds to the finger drop region, similar to ‘A’ region, expression levels of the genes were low in preclimacteric fruit but increased subsequently at the climacteric and particularly at the postclimacteric stage (Figure 9a–e, lanes 2, 4 and 6, Figure S10a–e). However, as compared to climacteric stage, no significant increase in *MA-ACS1* and *MA-ACO1* mRNA level was detected in postclimacteric fruit in ‘B’ region (Figure 9b and c, lanes 4 and 6). Low expression levels of the ripening genes particularly for *MA-ACS1*, *MA-ACO1*, *MA-SPS* and *MA-Exp* were detected in both peripheral and central regions of zones ‘C’, ‘D’ and ‘E’ of banana fruit at the preclimacteric stage (Figure 9g–k, lanes 1–6). At the climacteric stage, transcript levels of the ripening genes like *MA-SPS*, *MA-ACO1* and *MA-EXP* were low as compared to *MA-ACS1* and *MA-Lec* in both peripheral and central regions in zone ‘C’ of banana fruit. Transcript abundance of the genes then increased considerably particularly in the central regions of zones ‘D’ and ‘E’ (specifically) respectively as compared to respective peripheral regions (Figure 9m–q, lanes 1–6). In postclimacteric fruit, in addition to the central regions, expression levels of the ripening genes were increased in the peripheral regions of zones ‘C’ – ‘E’ as compared to the peripheral regions of zones C, D and E of climacteric fruit. However, in contrast to climacteric fruit, it was interesting to note that there was hardly any difference in expression levels of the genes between peripheral and central regions of zones C, D and E in postclimacteric fruit. Quantification of transcript levels has been shown in Figure S10. Overall, the expression profiles of the major ripening genes expressed during banana fruit ripening showed close similarity with the accumulation pattern of MA-MADS5 protein in the different regions of ripening banana fruit at various phases of ripening. Based on these observations and binding of MA-MADS5 to the CArG-box motifs in ripening gene promoters, we assumed that MA-MADS5 possibly involve in the regulation of expression of ripening related genes in banana fruit during ripening.

### Expression of MA-MADS5 in Floral Reproductive Organs

**Figure 10:**
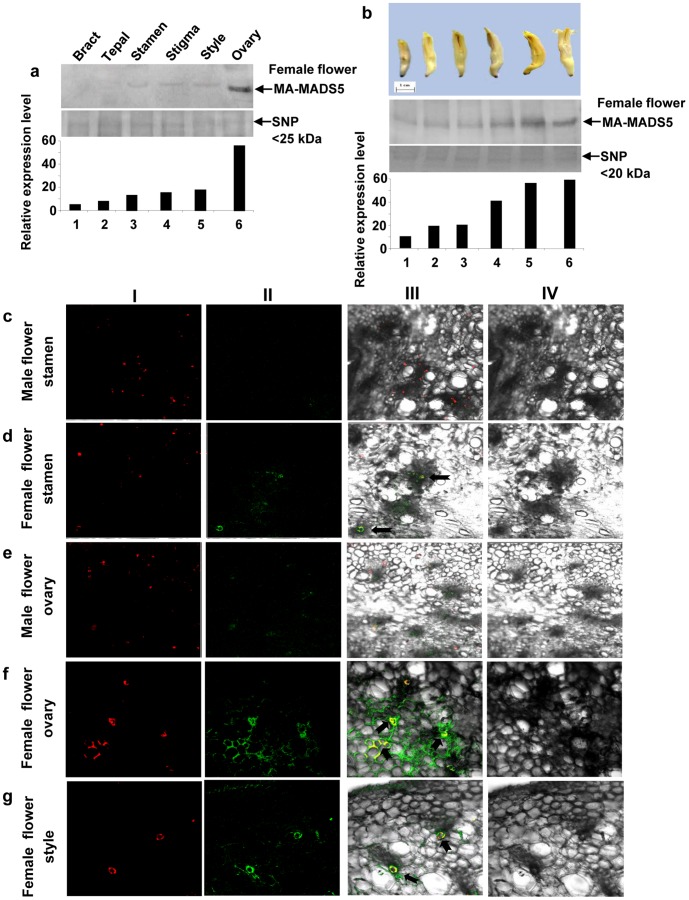
Immunodetection and *in situ* localization of MA-MADS5 protein in different floral tissues of banana. **a** Changes in the protein level of MA-MADS5 in different tissues of female flower (upper panel). Equal amount of small nuclear protein (SNP) lane was loaded in each lane and has been shown as loading control (middle panel). Protein abundance in each lane in (a) was detected by densitometry (lower panel). **b** Various stages of development of banana female flower (first panel). Changes in the accumulation levels of MA-MADS5 protein in different stages of developing female flower ovary tissues (second panel). Equal amount of small nuclear protein lane was loaded in lane and has been shown as loading control (third panel). Quantification of the data in the second panel by densitometry (lower panel). **c–g** Immunolocalization of MA-MADS5 protein in different tissues of banana flower including male flower stamen, female flower stamen, male flower ovary, female flower ovary and female flower style respectively. The legends of the panels I–IV for c-g were identical to those described in Figure 5b–e. Representative images from at least three independent experiments are shown.

Our results have indicated considerable level of CArG-box DNA binding activity in nuclear extract of banana mature female flower (Figure 1b). Moreover, *MA-MADS5* was found to be highly expressed in female flower ovary (Figure S6a). Therefore, we next investigated whether *MA-MADS5* plays any role in development of floral reproductive organ in banana. To study this possibility, we next analysed the expression levels of MA-MADS5 protein in various floral tissues of banana. Nuclear protein extracts were prepared from various parts of mature female flower for immunoblot analysis. As shown in Figure 10a **,** MA-MADS5 protein level was found to be significantly low or undetectable in the accessory floral parts like bracts and tepals, while low expression of the protein was detected in stamen of mature female flower (Figure 10a, lanes 1–3). MA-MADS5 protein was not detected in immature stamens of female flower (data not shown). On the other hand, whereas expression level of the protein was slightly higher in style and stigma tissues than stamen, significant level of MA-MADS5 protein was found to accumulate in mature ovary (Figure 10a, lanes 4–6). Furthermore, as shown in Figure 10b, in female flower, the abundance of this protein increased gradually along with the maturation stages of ovary and maximum expression was obtained in mature ovary (Figure 10b, lanes 1–6). Together, these results indicate that MA-MADS5 protein predominantly accumulates in ovary of mature female flower.

To further analyze the above results, we next investigated the sub-cellular localization pattern of MA-MADS5 protein in different floral tissues. We carried out immunolocalization studies using affinity resin purified, FITC-tagged (green fluorescence) anti-MA-MADS5 IgG as immunoprobe to detect intracellular location of MA-MADS5. As shown in Figure 10c and e, we could not able to detect any signal for MA-MADS5 in mature male flower stamen and ovary tissues, while very weak signals for cytosolic localization (only green fluorescence signals) were detected in stamen of mature female flower (Figure 10d)**.** In mature ovary tissue, nuclear localization signal for MA-MADS5 (yellow fluorescence spots indicated by arrow heads) was evident along with cytosolic signals (green fluorescence of FITC-tagged anti-MA-MADS5 antibody) (Figure 10f). Again, low levels of cytosolic and nuclear localization were also detected in style tissue of mature female flower (Figure 10g). No immunolocalization signal for MA-MADS5 was detected in bracts and tepals (not shown). In control experiments, we have used the FITC-linked pre-immune serum for immunolocalization which showed no *in-situ* binding to MA-MADS5 protein. Similarly, in another set of experiment, we pre-absorbed the FITC-linked anti-MA-MADS5 antibody to Protein-A agarose resin to trap the IgG in the resin. In this case, the serum eluted out from Protein-A resin was mostly devoid of the anti-MA-MADS5 IgG. The immunolocalization signals reduced significantly when MA-MADS5 IgG depleted serum was used for *in-situ* localization of MA-MADS5 in banana tissue (data not show). This observation confirmed the specificity of immunolocalization assays for MA-MADS5 protein in various tissues of banana using the FITC-tagged anti-MA-MADS5 IgG. Together, the increased accumulation level of MA-MADS5 protein in female flower ovary may indicate possible role of this protein in development of floral reproductive part like AG protein which has been shown to play important function in floral reproductive organ development in *Arabidopsis* [5]. However, further functional analysis may require establishing this assumption.

## Discussion

In this study, we have identified a CArG-box binding AGAMOUS-MADS domain protein from the flower and fruit tissue of banana Giant governor cultivar (*Musa sp*, AAA group, subgroup Cavendish) and investigated its possible function in fruit ripening and floral organ development. Previously, banana MADS-box protein MADS1 (AAY53908) was reported by Liu et al. [26] from banana (*Musa acuminata* L. AAA group, cv. Brazilian). Very recently Elitzur et al. [13] reported 6 MADS box genes (MA-MADS1–6) from banana (*Musa acuminata* AAA Cavendish subgroup, Grand Nain). Interestingly, MADS1 protein reported by Liu et al. [26] showed 98% amino acid sequence homology to MA-MADS5 (ACJ64682), while 34% sequence homology to MA-MADS1 (ACJ64679) reported by Elitzur et al. [13]. These observations indicated that MADS1 reported by Liu et al. [26] is actually more closely related to MA-MADS5 than MA-MADS1 reported by Elitzur et al. [13]. In our study, the identified MADS-domain protein showed ∼95% sequence homology with both MADS1 (AAY53908) [26] and MA-MADS5 (ACJ64682) [13]. On the other hand, it showed ∼33% amino acid sequence homology with MA-MADS1 (ACJ64679). On the other hand, the minor difference in sequence between MA-MADS5 (or MADS1) and the MADS-box protein identified in this study has been found to be mainly confined to single traces only which may appear as a consequence of alternative splicing. However, based on the overall high degree of sequence similarity, it appears that the AGAMOUS binding MADS-box gene identified in our study probably represent the *MA-MADS5* gene reported earlier from banana Grand Nain cultivar by Elitzur et al. [13]. Therefore, we have considered the identified gene as *MA-MADS5*, encoding an AGAMOUS MADS-box binding factor in banana (*Musa acuminata*) Giant Governor Cultivar. In addition, it is also interesting to indicate here that although we observed some difference in the expression patterns of *MA-MADS5* in the present study with those in previous report [13] particularly during banana fruit ripening, considerable expression level of the gene has been detected in banana female flower ovary in both cases. Therefore, the difference in the expression patterns of *MA-MADS5* may appear due to cultivar difference and difference in ripening pattern of fruit in two different cultivars.

Extensive molecular and biophysical studies of MADS-box transcription factors family have demonstrated the importance of dimer formation as the basic functional unit for the activity of MADS-box transcription factors [21]. Analyses with crystal structures of MADS-box domains have revealed that dimer of MADS-box binds to CArG-box motif [25], [37]. In plants, MADS-box proteins like APETALA1 (AP1), AG, SEP1, SQUAMOSA (SQUA) binds to DNA as homodimers [22], [23]. Conversely, other MADS-domain proteins like APETALA3 (AP3), PI and GLOBOSA (GLO) bind to DNA only as heterodimers with their counterparts [38]. In this study, we have shown that MA-MADS5 protein forms stable homodimer *in vitro* to bind to CArG-box DNA. Furthermore, studies with deletion versions of recombinant MA-MADS5 proteins have indicated that MADS, I and K domains were required for dimer formation while the I-region along with the MADS-domain appeared to be essential for binding to the CArG-box motif.

MADS-box transcription factor binding mediated DNA bending has been exclusively studied using human MADS-domain transcription factor SRF [39]. Among MADS-box proteins in plants, *Arabidopsis thaliana* MADS-box proteins AP1, AG, AP3/PI [38] and *Antirrhinum majus* MADS-box proteins DEF/GLO, PLE and SQUA [19] have been shown to introduce significant level of bending upon binding to DNA. In this study we have investigated how MA-MADS5 induces DNA bending upon binding to specific sequence. To study this aspect, we have used two different types of MADS-box protein binding sites: *c-fos* SRE with the consensus CArG box sequence CC(AT)_6_GG and N10 [39]. Our results of DNA bending analyses have revealed the ability of MA-MADS5 protein to induce significant level of DNA bending upon binding to both N10 and SRE sites. The overall bend angle was 93° in case of N10 and 41° in case of SRE. In contrast to SRF, MA-MADS5 protein induced bends on DNA in a sequence dependent manner, which was similar to the SQUA. Our results have also demonstrated that the purified minimal DNA-binding domain of banana MADS-box protein (MADS and I domains) bends DNA in similar way as like the full length protein (data not shown). Furthermore, phasing analysis has confirmed that MA-MADS5 induces DNA bending.

Six MADS-box genes (*MA-MADS1-6*) have recently been isolated from banana fruit and their expression patterns were studied during banana fruit ripening [13]. *MA-MADS1-3* were found to be highly expressed in fruit tissues while *MA-MADS4-6*, besides being expressed in fruit, showed expression in other tissues. In many other cases, expression of MADS-box genes was not found to be associated with specific tissues while shown to be recruited for various functional tasks [40]. In tomato, *TAGL12*, besides being expressed in fruit, also found to be expressed in other tissues [41]. In this study, we observed higher expression levels of *MA-MADS5* in female flower ovary and fruit pulp at the onset of climacteric phase, while expression level was relatively low in peel at climacteric peak (Figure S6). Similar to other MADS-box genes reported earlier from banana [13], in fruit pulp, *MA-MADS5* expression level showed dynamic change during ripening. It has also been demonstrated that MADS-box genes particularly belonging to similar clade and with similar expression pattern may have functional redundancy [24]. The proteins encoded from these genes have been suggested to form different heterodimers in peel and pulp during ripening. In addition, changes in expression levels of MADS-box genes in banana fruit may indicate dynamic changes in transcriptional complexes formed during ripening [13].

Immunoblotting experiments and *in situ* localization analyses have revealed increased accumulation level of MA-MADS5 protein in the central parts of the middle and bottom regions (zones D and E) of banana fruit pulp at the climacteric phase of ripening. These observations have provided important clue to indicate possible involvement of *MA-MADS5* gene in banana fruit ripening. However, it important to note that, for *in-situ* analyses, we have used affinity purified FITC-tagged anti-MA-MADS5 IgG. Therefore, possible cross-reactivity of the antibody with the other yet unidentified AG-like protein (s) express in banana fruit may not be completely ruled out.

Earlier studies in tomato have demonstrated interaction of the RIN MADS-box transcription factor with the promoters of genes involved in ripening and associated major pathways like transcriptional control network involved in overall regulation of ripening, ethylene biosynthesis, ethylene perception, downstream ethylene response, cell wall metabolism, and carotenoid biosynthesis [42]. In our study we have found that the transcript accumulation patterns of five key ripening genes in different regions of banana fruit pulp were very similar with the expression levels of MA-MADS5 protein. Furthermore, our ChIP assay results have demonstrated that MA-MADS5 protein indeed bind specifically to the CArG-box elements present in the promoters of major ripening genes in banana (Figure 7b). These observations may suggest possible involvement of MA-MADS5 in the transcriptional regulation of major ripening genes in banana fruit.

The expression patterns of *MA-ACS1* and *MA-ACO1*, which play key role in ripening ethylene production in banana fruit, were consistent with the accumulation level of MA-MADS5 protein in various regions of fruit pulp during ripening. This observation may suggest direct involvement of MA-MADS5 in transcriptional regulation of *MA-ACS1* and *MA-ACO1* during ripening. On the other hand, function of *SPS*, *Exp* and *Lec* in banana is mainly associated with the later part of ripening, including synthesis of sugar to confer sweetness to fruit, binding of lectins to sugar and fruit softening [32], [43], [44]. Consistent with this observation, expression levels of *MA-SPS*, *MA-Exp* and *MA-lec* transcripts were relatively higher in various regions of pulp at the postclimacteric phase than the climacteric phase. In contrast, although ChIP assay results indicate specific binding of MA-MADS5 to the CArG box motifs in the promoters of *MA-SPS*, *MA-Exp a*nd *MA-Lec*; accumulation of MA-MADS5 decreased in all regions of fruit at postclimacteric stage. However, several assumptions may be predicted to explain the observed discrepancy in expression of MA-MADS5 and its targets. First, we have noted that the promoters of the five ripening genes in banana contain potential ethylene responsive elements (EREs) and their expressions were ethylene inducible. Therefore, after ethylene bursting at the climacteric phase, this endogenous ethylene is also able to activate ERE binding and transcriptional stimulation of *MA-ACS1* and *MA-ACO1* at climacteric and *MA-SPS*, *MA-Exp* and *MA-Lec* after climacteric peak. Therefore, a complex regulatory network involving the interactions of MA-MADS5 transcription factor (whose expression has also been found to be regulated by ethylene) and EREBPs (ERE binding proteins) may initiate the transcription of these ripening genes in banana after the perception of ethylene signal. Furthermore, at least three MADS-box genes belonging to SEP3 clade, *MA-MADS1*, *MA-MADS2* and *MA-MADS3* are highly expressed in ripening banana fruit. The SEP genes have been shown to often retain similar functional capacity and participate in the creation of multimeric complex [5]. On the other hand, MA-MADS5 has been found to form homodimer *in vitro* to bind to CArG-box sequence. Therefore, based on this information, we assumed the possibility that proteins encoded by *MA-MADS1-3*, expressing highly in banana fruit, may form heterodimers with MA-MADS5 *in vivo*.

One sub lineage of MADS box genes, the AGAMOUS clade, has been demonstrated to play key roles in regulating many aspects of flower and fruit development in angiosperms. Gene duplication in the AG clade has been shown to result in the euAG and PLE lineages within the core eudicots in angiosperms [45]. In *Antirrhinum majus*, FARINELLI (FAR) represent the euAG lineage gene, while PLENA (PLE) corresponds to the PLE lineage gene [46]. Loss-of function analyses have revealed role of PLE in stamen and carpel development and FAR has been shown to be involved in pollen development in the stamens [47], [48]. In *Arabidopsis*, the floral homeotic gene *AGAMOUS* (*AG*) plays a central role in reproductive organ (stamen and carpel) development [49]. A recent duplication has been shown to result in two paralagous PLE lineage genes in *Arabidopsis*, SHATTERPROOF 1 and SHATTERPROOF 2 (*SHP1* and *SHP2*, previously described as *AGL1* and *AGL5*, respectively). *SHP1* and *SHP2* were shown to be specifically expressed in carpel [50], [51] and shown to be redundantly required for dehiscence zone formation in the silique, as well as aspects of ovule development in *Arabidopsis* [52]. Interestingly, no mutants of AG clade genes have been reported in tomato. However, previously AG lineage genes have been identified and their expression patterns have been characterized in detail in tomato [41], [53]. *TAG1* belongs to the euAG clade [8]. Loss of *TAG1* function has been shown to be associated with homeotic transformations of stamens and carpels [54]. Previous studies involving functional analyses of *TAGL1*, using RNA interference or by repressing its function using a dominant chimeric repressor construct have revealed unique role of *TAGL1* in regulating several aspects of ripening, mainly carotenoid accumulation, fleshy fruit expansion, and ethylene production [8], [55]. A more recent study involving loss-of-function analyses of *TAG1* and *TAGL1* in the same genetic background in tomato (cv MicroTom) using RNAi have demonstrated role of *TAGL1* in regulating tomato fruit ripening, while *TAG1* has been shown to be involved in specifying normal stamen and carpel development [56].

In this study, the AGOMOUS MADS-box gene, *MA-MADS5*, identified in banana fruit and floral tissues, has been found to share close phylogenetic similarity with AG clade genes reported from other plants (Figure S3a). Furthermore, appreciable level of accumulation of MA-MADS5 protein in banana fruit drop zone (B zone) was interesting in relation to the function of two previously described AG subfamily genes like *SHP1* and *SHP2*, which have been shown to be redundantly involved in dehiscence zone formation in fruits in *Arabidopsis*. Interestingly, repression of *NTNAG1*, an *AG* gene in tobacco, in transgenic tobacco lines expressing the antisense *MA-MADS5* construct have resulted in altered flower and fruit morphology with delayed flowering and reduction of fruit size (unpublished data). Together, our results have provided interesting information for further study of functional relevance of *MA-MADS5* in fruit ripening and floral reproductive organ development. Transgenic banana with reduced expression of *MA-MADS5* will provide the system to further study these functions in detail.

## Materials and Methods

### Plant Material

The banana cultivars Giant governor *(Musa sp*, AAA group, subgroup Cavendish) and Monthan (ABB group, cooking banana) were obtained from West Bengal State Council of Science and Technology, India. Plants were grown in soil from August to April at the Bose Institute Experimental field under the conditions described earlier [29]. To study the transcript and protein expression pattern during *ex-planta* ripening, unripe green bananas (pre climacteric stage) were harvested and the hands were cut from a bunch of 80 days post anthesis (DPA) to avoid heterogeneity due to differences during development. Each banana hand was separated into individual fingers and kept at room temperature (25°C) until the fruit were fully ripe (15 d after harvest). For each experiment, banana fingers from a same hand representing the similar developmental stage were used as a sample group to rule out difference in ripening behaviors of fingers among different hands [57]. Different parts of floral and fruit tissues of banana (at different DPA) were collected from various stages of ripening, frozen in liquid N_2_ and stored at −80°C for isolation of RNA, crude and nuclear protein extracts. Except the isolation processes, all other experiments were repeated at least three times.

### DNA-binding Assay and South-Western Blotting

Nuclear protein was isolated from banana flower tissues and fruit pulp as described previously [29]. The synthetic promoter element AGAMOUS specific CArG box motif (AGAMOUS element 1) and 17 LS (Table S3B) with complementary sequence at the 3′ end of AGAMOUS element 1 and 3′ end of 17 LS were annealed by slow cooling after heating at 90°C and then filled in with Klenow enzyme [58]. The 22-bp synthetic dephosphorylated oligonucleotides, each containing a specific CArG-box *cis*-element, detected in the promoter of five ripening specific genes (Table S3B), were 5′ end labeled with [*γ*-^32P^] ATP by T4 PNK (T4 polynucleotide kinase) (Amersham Biosciences, UK) as described previously [29]. The radio-labeled DNA probes were purified by using Sephadex-G-50 quick spin column (Roche, Germany) following the manufacturer’s instruction. DNA-binding assays were performed with 15 µg nuclear protein extract or 2 µg purified recombinant protein by essentially following the protocol described previously [29]. South-Western blotting was carried out by using equal amounts of nuclear proteins (25 µg) and following the protocol described previously [29]. The sequences of oligonucleotides used in DNA-protein interaction studies are summarized in Table S3B.

### Generation of Transgenic Tobacco Plant with *3X CArG-GUS* Synthetic Promoter-reporter Construct and Detection of GUS Activity

For functional characterization of CArG-box motif, synthetic promoter fragment was designed which contained three copies of CArG box motifs in tandem with 3′ ends providing the basal promoter up to −70 region from CaMV 35S promoter. The 70LS-CaMV basal promoter 1/1 primer was designed in a way that it has complementary region at the 3′ region with the 3′ region of AGAMOUS element 1/1 primer and AGAMOUS element 1/1 m1 primers, respectively (containing typical triplicate version of AGAMOUS binding CArG-box sequence and its mutated form). The two oligonucleotides (AGAMOUS element 1/1 and 70 LS-CaMV basal promoter 1/1 or mutant AGAMOUS element 1/1 m1 and 70 LS-CaMV basal promoter 1/1 (Table S3D) were annealed by heating at 90°C for 5 min followed by slow cooling to room temperature and then filling in with Klenow enzyme (Promega). Two other synthetic promoter fragments were generated (SPS UP MDBE and 70 LS-CaMV basal promoter 1/1; mutant SPS UP MDBE and 70 LS–CaMV basal promoter 1/1, Table S3D) in similar way. The synthetic promoters were cloned into the *HindIII-BamHI* sites of pBI121 (Stratagen) by removing the CaMV 35S constitutive promoter. The recombinant plasmids were then individually introduced into *Agrobacterium* strain LBA4404. The promoter-reporter constructs were individually introduced into tobacco plants by *Agrobacterium* (Strain LBA4404) mediated leaf disc infection-co-culture method [30]. Kanamycin resistant plants were examined for transgene integration by genomic PCR using 70 UF and GUS R oligos (Table S3D). Total genomic DNA and RNA were isolated from the transgenic tobacco lines following the method of Roy Choudhury et al. [34] and *GUS* expression was detected by RT-PCR using GUS F (forward) and GUS R (reverse) oligos (Table S3D). GUS activity measurement was performed following the method of Chattopadhyay et al. [59].

### Elution of Protein Fraction from the DNA-protein Complex of Dried EMSA Gel and Protein Identification by Mass Spectrometry

The AGAMOUS element binding MADS-domain transcription was isolated from the DNA-protein complex of dried EMSA gel following the method described by Stenger et al. [60]. The gel slice originating from the dried gel was allowed to rehydrate in sterile milli-Q-water for 10 min. Following rehydration, the gel piece and the Whatman paper were separated by using sterile forceps. The gel piece was subsequently subjected to in gel tryptic digestion by essentially following the method described previously [61]. Tryptic digest products were analyzed by matrix-assisted laser desorption/ionization time-of-flight (MALDI-TOF) mass spectrometry (Autoflex II, Brucker Daltonics, Bremen, Germany). Proteins were identified by peptide mass fingerprints, generated via MALDI-TOF MS and MS/MS for peptide sequencing using the Mascot search engine (http://www.matrix science.com, Matrix Science, London, England) and Viridiplantae (Green Plants) protein database at NCBI. The Mascot search parameters were as follows: type of search, Peptide Mass Fingerprint and MS/MS ion search; enzyme, trypsin; mass value, monoisotopic; protein mass, unrestricted; peptide mass tolerance, ±0.3 ppm; peptide charge state, 1+; max missed cleavage, 3 per peptide; maximum allowed peptide mass error of 100 ppm; Instrument type: MALDI-TOF/TOF. According to MASCOT probability analysis, only significant (*P*<0.05) hit was accepted.

### Isolation and Molecular Cloning of cDNA Encoding CArG-box Element Binding AGAMOUS MADS-box Protein from Banana Fruit

Total RNA was isolated from pulp tissue of banana fruits by following the protocol described previously [29]. The first strand cDNA was synthesized from 2 µg of total RNA (DNase I treated) using Thermoscript reverse transcriptase kit (Life Technologies, USA) following manufacturer’s instruction. MS analysis has revealed 43% sequence coverage of the identified peptides against the matched protein (MADS-box protein MADS1 from banana, Swiss-Prot accession- Q4TTS9_MUSAC) (Figure 1f). Therefore, primers were designed based on matched peptide sequences corresponding to the N- and C-terminal regions of the protein. We next used rapid amplification of cDNA ends (RACE) to obtain the full length cDNA of the identified MADS-box protein from banana fruit. 5′ and 3′ RACE were carried out using the double stranded partial cDNA as template. The partial cDNA was amplified using oligonucleotides AUAP from the 3′ RACE system (Life Technologies, USA) and MD A primer (5′ GAACGAGTGCAGCAACTGA 3′). The PCR products were cloned into pBluescript cloning vector (Stratagen, Heidelberg, Germany) for sequence analysis by T3 and T7 primers (Bangalore Genei, India). Utilizing the nucleotide sequence of the cDNA clone obtained in 3′ RACE, sequence specific primers MD B and MD C (Table S3A) were designed and 5′ RACE was performed (Life Technologies, USA). Based on the 5′ and 3′ end sequences of the cDNA, a pair of primer was designed (MD F and MD R) for amplification of the entire open reading frame. The reaction for RT-PCR for obtaining the full coding sequence was subjected to 30 cycles of 94°C for 1 min, 59°C for 1 min and 72°C for 2 min using the primer MD F and MD R (Table S3A). The amplified product was cloned into pDRIVE U-A cloning vector (Qiagen) and sequenced, which revealed the coding sequence (732 bp) of an AGAMOUS-MADS box transcription factor (ADW08393) of banana (*Musa acuminata* AAA Group).

The transcript expression profiles of AGAMOUS-MADS box transcription factor in various tissues of banana and the message levels of *MA-ACS1*, *MA-ACO1*, *MA-SPS*, *MA-Lec* and *MA-Exp* in various parts of banana fruit during ripening were analyzed by semi-quantitative RT-PCR (Text S1).

### *In silico* Analysis of AGAMOUS-MADS Box Transcription Factor Sequence

*Blastn* analysis of the isolated cDNA sequence of banana AGAMOUS-MADS box transcription factor at the National Center for Biotechnology Information (NCBI) showed ∼95% of sequence similarity (699/732) to *MA-MADS5* (EU869310) and *MADS box protein MADS1* (697/732) (DQ060444), ∼77% (564/732) to MADS-box protein 2 encoding gene of *Lilium longiflorum* (AY522502), and ∼50% (367/732) to SEEDSTICK-like protein of *Prunus serrulata* (GU332504) in NCBI blast analysis [62]. Based on the sequence similarity results we considered the cDNA for banana (*Musa acuminata*) AGAMOUS-MADS box transcription factor as *MA-MADS5*. The sequence was virtually translated and aligned with the protein sequences of representative MADS-box genes from different plant species using the ClustalW [63] with default parameters. We then constructed a Neighbor-Joining (NJ) tree with p-distance using the MEGA4 [64]. The phylogenetic tree was tested by bootstrap analysis with 1000 replications.

### Expression and Purification of Recombinant Proteins

The full length *MA-MADS5* cDNA (corresponds to 732 bp ORF) was cloned into the *BamHI- HindIII* sites of pQE30 bacterial expression vector (Qiagen). Several deletion versions of *MA-MADS5* cDNA were generated by PCR amplification using gene specific primers with the 732 bp cloned cDNA fragment as template. The primers used for generation of deletion versions of *MA-MADS5* cDNA are listed in Table S3A. The full length and deletion constructs (cloned in to the *BamHI-HindIII* sites of pQE30 [Qiagen]) of *MA-MADS5* were individually introduced into *E coli* M15 (pREP4) host strain (Qiagen) for overexpression of 6X-His tagged recombinant proteins. Recombinant proteins were induced with the addition of 1 mM IPTG following standard protocol. Recombinant proteins were purified by Ni^2+^-NTA resin (Qiagen) following manufacturer’s instructions.

### Circular Permutation Analysis

For circular permutation analysis, the DNA fragments, carrying the transcription factor binding sites, were isolated from pAS152 and pAS76 vectors by digestion with the appropriate restriction enzymes (including *MluI, BglII, XhoI, EcoRV, SmaI, StuI, RsaI and BamHI*), purified via native 10% polyacrylamide gel, then dephosphorylated by calf intestinal alkaline phosphatase and end-labeled using T4 PNK in presence of [γ-^32^P] ATP. DNA binding reactions were carried out essentially as described previously [27]. The protein-DNA complexes and free DNAs were resolved by electrophoresis through 6% non-denaturing polyacrylamide gels and visualized by autoradiography. The magnitude of apparent DNA bending was calculated from the variation in the mobilities of protein-DNA complexes using the formula µM/µE = cos (α/2), where µM and µE correspond to the relative mobilities of the protein-DNA complexes and α stands for the induced bend angle. The values of µM and µE (relative mobility of the slowest and fastest migrating species) were calculated from the curve produced after fitting the data to a cosine function [65]. Curve fitting was carried out using Origin 6 software. Bend angles are given as the averages of three independent experiments.

### Phasing Analysis and Ligase-mediated Circularization Assay

For phasing analysis the plasmids pAS469-pAS474 were constructed by ligating the two annealed phosphorylated oligonucleotides ADS339 (5′-AATTAGGAAAACTATTTATAGATCAAATGAGCT-3′) and ADS340 (5′-CATTTGATCTATAAATAGTTTTCCT-3′) into the phasing vectors SB12, -14, -16, -18 and -20 [66]. These oligonucleotides contained the MEF2A binding site N10 which is underlined. DNA fragments were synthesized by PCR using the primers (ADS346∶5′-GGCTACAATGAATTCATAACCTT-3′ and ADS347∶5′-ATCGAAATGAATTCGACTCAC-3′). PCR products were subsequently digested with *EcoRI* and gel purified to generate the 230 to 238 bp products. These fragments were then radiolabeled with [γ-^32^P] ATP using T4 PNK. The 5′ and 3′ ends were 62 and 64 bp from the centers of the N10 site and the terminal A:T tract respectively. DNA binding reactions were performed in presence of wild type recombinant protein with radiolabeled 230 to 238 bp products containing the MEF2A binding site N10. The phasing vectors were constructed in a way that they have different lengths of linker between hexameric [A:T] tracts and N10 site. The linker length varies between 12 and 20 bp, giving rise to a spacing of between 51 to 59 bp between the centre of the first hexameric [A:T] tract and the centre of the N10 (CArG box), as a result different sized products (230–238 bp) were generated by PCR from these vectors. The resulting DNA-protein complexes were then resolved by 6% non-denaturing polyacrylamide gel electrophoresis, then gel was then dried and autoradiographed.

For ligase-mediate circularization assays, recombinant proteins were pre-incubated with phasing DNA probes in 50 µl gel retardation buffer (2 mM sperminidine, 60 mM KCl, 8 mM HEPES pH 7.9, 6.4% glycerol, 0.64 mM MgCl_2_, 0.32 mM DTT and 0.032 µM ZnCl_2_) for 30 min at 25°C and then placed on ice. Ligation reactions were initiated by the addition of an equal volume of ligation buffer (0.5 mM MgCl_2,_ 0.2 mM ATP, 0.1 mM DTT, 0.001% Nonidet P-40, 0.1 mM spermidine, 0.4 mM HEPES pH 7.9, 0.32% glycerol, 0.0016 µM ZnCl_2,_ 4 µg of BSA) and T4 DNA ligase (Fermentas). Each phasing probe tested re-circularized at a different rate in the absence of added binding protein. 10 µl samples were taken between 0 and 60 min and quenched with 5 µl of 75 mM EDTA, 2 mg/ml Proteinase K and 15% glycerol containing 0.2% xylene cyanol, 0.2% bromophenol blue. The samples were incubated at 55°C for 15 min and then separated on 6% non-denaturing polyacrylamide gel. A second set of sample was taken after 60 min, incubated at 65°C for 10 min followed by digestion with 1.6 U of Exonuclease III (Fermentas) at 37°C for 30 min. This reaction was performed to remove the linear DNA and to identify circular reaction products. The gel was then dried and exposed to X-ray film.

### Generation of Antibody Against Banana AGAMOUS-MADS Box Transcription Factor

All MADS-box proteins contain a highly conserved homologous MADS-box domain at the N-terminal region, while the C-terminal domain has been shown to be highly variable. Therefore, the antibody against MA-MADS5 protein was raised against the C-terminal region of the protein. We have used affinity resin purified recombinant partial MA-MADS5 protein corresponding to C-terminal 170 amino acids (74 aa–243 aa) for generation of the antibody. A male rabbit was immunized with affinity resin purified recombinant partial MA-MADS5 using multiple intra-dermal injection methods. The subsequent steps of immunization and generation of primary immune serum against MA-MADS5 were carried out by essentially following the method described by Lane and Harlow [67]. Affinity purification of immune serum was carried out using Protein A-Agarose Fast Flow resin (Sigma) by Bangalore Genei, Bangalore, India.

### Immunoblotting and Immunolocalization

Crude protein extracts and nuclear protein extracts were prepared by following the method as described previously [29], [36]. Protein concentration was determined using the Bradford protein assay kit (Bio-Rad) with BSA (Fraction V, Sigma) as the standard. Plant protein or bacterially expressed recombinant protein samples were separated in 12% SDS-PAGE and protein gel blot analysis was carried out by essentially following the method described previously with affinity resin purified anti-MA-MADS5 polyclonal antibody (1∶1000 dilution) as the primary antibody and Goat-anti-rabbit IgG conjugated with alkaline phosphatase (1∶1000 dilution) as the secondary antibody [34].

Immunolocalization studies for *in situ* localization of MA-MADS5 protein in various tissues of banana flower and fruits were carried out by essentially following the protocol of Paciorek et al. [68]. FITC-couples affinity purified anti-MA-MADS5 IgG was used to detect MA-MADS5 in the different tissues of banana. FITC-labeling of anti-MA-MADS5 IgG was carried out using 1 mg/ml of affinity purified anti-MA-MADS5 IgG and following the protocol of Harlow and Lane [66]. Unlabeled free FITC was removed by passing the labeling mixture through Sephadex G-25 column (25×2.0 cm). Percentage of FITC incorporation of FITC tagged to anti-MA-MADS5 IgG was calculated from the UV spectral peaks of anti-MA-MADS5 IgG and FITC at 280 and 495 nm respectively [69]. After serial incubation steps of tissue sections in 2% BSA solution (blocking solution), FITC-coupled anti-MA-MADS5 IgG solution and DAPI solution (1 µg/µl in 1× PBS), samples were washed with sterile milli-Q water for five times, 5 min each at room temperature to confirm removal of excess of stain. A drop of antifade medium (Keiser’s glycerol gelatine, Merck, Germany) was added to the sections on the slide and gently covered with a cover glass (22 mm×22 mm). The slides were examined in confocal laser scanning microscopy (Zeiss LSM-510 meta, Germany). Images were taken at 20-fold magnification with an excitation wavelength of 488 nm for FITC and 405 nm for DAPI and an emission wavelength of 405–450 nm for FITC and LP420 nm for DAPI respectively. Images were captured from five randomly selected regions for each sample. Three replicates were taken for each sample. In all the independent technical and biological trials we have observed the similar staining pattern as shown in the indicated figures.

### Chromatin Immunoprecipitation

To cross-link genomic DNA and protein in fruit tissue, sliced fruit pulp from preclimacteric and climacteric stages was submerged in extraction buffer [0.4 M sucrose, 10 mM Tris–HCl, pH 8.0, 5 mM b-mercaptoethanol, 0.1 mM PMSF and proteinase inhibitor cocktail with 1% formaldehyde] and vacuumed for 10 min. The cross-linking reaction was stopped by adding glycine to a final concentration of 0.125 M and application of vacuum for an additional 5 min. After rinsing with ice-cold water, fruit tissue was frozen in liquid nitrogen. Chromatin isolation was performed by the method described previously [70]. The chromatin solution was sonicated by using a Sonifier 450 (Branson, http://www.sonifier.com). The sonicated chromatin suspension was immunoprecipitated with anti-MA-MADS5 serum or pre-immune serum, and DNA was recovered using the method described previously [70].

## Supporting Information

Figure S1 **Detection of GUS activity in different tissues of transgenic tobacco lines.** Measurement of GUS activity in the leaf, flower and fruit tissues of transgenic tobacco lines carrying the trimeric version of *Arabidopsis* Agamous MADS box binding element (3XCArG:*GUS* construct) in fusion with the GUS reporter gene.
(TIF)

Figure S2 **Identification of 27-kDa banana Agamous MADS box element binding protein from climacteric pulp tissue by mass spectrometry. a** Eluted protein fractions from DNA-protein complexes of dried EMSA gel (flower, lane 2 and climacteric fruit pulp, lane 3) were concentrated, desalted and then resolved in 10% SDS-PAGE followed by staining of the gel with silver salt. Arrow indicates the position of 27-kDa single protein on gel. **b** Observed and expected monoisotopic [M+H]^+^ masses of ions from the tryptic digest of the Agamous MADS box element binding protein.
(TIF)

Figure S3 **Phylogenetic analysis of** ***Musa acuminata*** **27-kDa agamous MADS box element binding protein.** **a** A consensus bootstrap neighbour-joining tree based on the CLUSTALW alignments of the amino acid sequences of MADS box from different organisms has been constructed by MEGA version 4.0. The sequences which have been used for phylogenetic tree formation have been summarized in Table S2. **b** A consensus bootstrap neighbour-joining tree based on the CLUSTALW alignments of the amino acid sequences of banana MADS box has been constructed by MEGA version 4.0.
(TIF)

Figure S4 **Over-expression of full-length MA-MADS5 protein in** ***E.coli*** **.** **a** Separation of noninduced control (lane 1) and IPTG induced (lane 2) total protein extracts, obtained from M15 (Prep4) *E.coli* cells by 12% SDS-PAGE and stained with coomassie blue. After cell lysis small amount of recombinant protein remained in soluble fraction (lane 3), while considerable amount of recombinant protein remained insoluble fraction (lane 4). **b** The soluble fraction was further purified by Ni-NTA chromatography. Several wash fractions (lanes 1–3) and elution fractions (lanes 4–6) were detected. Arrow indicating the position of recombinant His-tagged MA-MADS5 protein on the gel.
(TIF)

Figure S5 **Detection of relative binding ability of MA-MADS5 protein to N10 and SRE DNA probe.** Saturation binding assay in case of N10 and SRE were carried out by incubating a fixed amount of wild type recombinant proteins (MA-MADS5) with increasing amounts of radiolabeled N10 and SRE. The apparent BMAX and KD values were created in each case by Graph Pad Prism v4.0.
(TIF)

Figure S6 **Transcript accumulation profiles of** ***MA-MADS5*** **. a** Transcript accumulation profiles of *MA-MADS5* in different tissues of banana (cultivar giant governor) by semi quantitative RT-PCR. Transcript profile of *MA-Actin* was used as internal control in middle panel. Quantification of data in (**a**) by densitometry (Bio-Rad Image Densitometer) (lower panel). **b and c.** Changes in accumulation pattern of *MA-MADS5* mRNA in the peel and pulp tissues during ripening in banana fruit ripened naturally at 25°C both. Transcript accumulation profiles for both the tissues were analyzed by semi quantitative RT-PCR with gene-specific primers. Transcript level of *MA-Actin* was used as internal control (middle panel). Transcript abundance of *MA-MADS5* in each lane was detected by densitometry (Bio-Rad Image Densitometer) (lower panel). **d and e** Changes in the abundance of *MA-MADS5* mRNA in the pulp and peel tissues of banana following ethylene treatment. Transcript abundance of *MA-Actin* was measured as internal control (middle panel). Relative transcript level of *MA-MADS5* was detected by densitometry (lower panel). Representative gel images from three independent trials have been shown.
(TIF)

Figure S7 **Detection of specificity of anti-MA-MADS5 polyclonal antibody.** **a** Immunoblot analysis with nuclear protein extracts isolated from preclimacteric, climacteric and postclimacteric banana fruit pulp (lanes 1–3). ∼25 µg of nuclear extract was loaded in each lane and then immunoblotting was performed using anti-MA-MADS5 polyclonal antibody. **b** Equal amounts (2 µg) of purified wild type and deletion versions of MA-MADS5 recombinant proteins were loaded on 10% polyacrylamide gel and then immunoblotting was performed using anti-MA-MADS5 polyclonal antibody. **c** The banana MA-MADS2 protein was expressed in *E. coli.* The affinity purified (4 and 8 µg) recombinant proteins were resolved on 10% polyacrylamide gel and then immunoblotting was performed using anti-MA-MADS5 polyclonal antibody. **d** Equal amount (10 µg) of purified wild type and three deletion versions of MA-MADS5 recombinant proteins (A, D, E, F) were immunoprecipitated with anti-MA-MADS5 polyclonal antibody. The proteins in immunocomplex were subjected to immunoblotting using anti-MA-MADS5 polyclonal antibody.
(TIF)

Figure S8 **Gel mobility shift assay using CArG-box motifs detected in the promoter of different ripening specific genes.** **a** 15 µg climacteric fruit pulp nuclear protein extract was incubated with the different CArG-box sequences, detected from various ripening gene promoters, as probes. The specific CArG-box motif which was used as probe indicated by numbers under the figure: CArG-box motif 1(lane 1), 2 (lane 2), 5 (lane 3), 7 (lane 4) and 8 (lane 4). **b** 15 µg climacteric fruit pulp nuclear protein extract was incubated with the different probes in presence of 100 molar excess of unlabeled same CArG-box motifs [1(lane 1), 2 (lane 2), 5 (lane 3), 7 (lane 4) and 8 (lane 4)] which were used as competitor. **c** In the similar gel mobility shift assay, 15 µg climacteric fruit pulp nuclear protein extract was incubated with the different probes in presence of 100 molar excess of unlabeled corresponding mutant CArG-box motifs which were used as competitor. **d** Gel mobility shift assay using different CArG-box motifs 3 (lane 1), 4 (lane 2), 6 (lane 3) and 9 (lane 4) used as probe. 15 µg climacteric fruit pulp nuclear protein extract was loaded in each lane. The specific CArG-box which was used as probe, has been indicated by numbers under the figure.
(TIF)

Figure S9 **Analysis of activity of trimeric CArG box motif (3XCArG) of banana** ***SPS*** **promoter in transgenic tobacco lines.** **a** The trimeric wild type and mutant CArG box motifs (pMDBE:GUS, pMDBE_m_:GUS) of banana *SPS* promoter fragment were fused to *GUS* reporter. **b** Stages of regeneration of transgenic tobacco plants after infection of tobacco leaf disc with *Agrobacterium* containing pMDBE:GUS constructs; Regeneration, shooting and rooting, hardening, mature stage in pot containing soil. **c** The integration of transgenes in transgenic tobacco lines were detected by genomic PCR with CArG box motif specific forward primer (70 UF) and GUS specific reverse primer (Table S3D). **d** Detection of *GUS* transcript level by semi quantitative RT-PCR in control (lane 2) and transgenic tobacco lines carrying trimeric wild type CArG box motif (lanes 3–6). Lane 1 indicates DNA molecular weight marker. **e** Detection of GUS activity in the leaves of control and transgenic tobacco lines carrying pMDBE:*GUS* or pMDBE_m_:*GUS* constructs. **f** Analysis of expression pattern of *GUS* transcript in different tissues of transgenic tobacco lines with wild type CArG-box motif (pMDBE:*GUS*) (upper panel, lanes 2–6) and mutant CArG box motif (pMDBE_m_:*GUS*) containing transgenic lines (lower panel, lanes 2–6) by semi quantitative RT-PCR. Lane 1 indicates DNA molecular weight marker. **g** GUS activity was detected in the leaf, flower and fruit tissues of transgenic tobacco lines carrying trimeric wild type CArG box motif.
(TIF)

Figure S10 **Transcript accumulation profiles major ripening genes in different zones of banana fruit. a-e** Semi-quantitative RT-PCR analysis was carried out to detect the changes in transcript levels of *MA-SPS, MA-ACS1*, *MA-ACO1*, *MA-EXP* and *MA-LEC* in A and B zones of preclimacteric, climacteric and postclimacteric banana fruit. Transcript levels of *MA-SPS, MA-ACS1*, *MA-ACO1*, *MA-EXP* and *MA-LEC* ripening genes from peripheral and central regions of C, D and E zones of (f–j) preclimacteric (0 DAH), (k–o) climacteric (88 DAH) and (p–t) postclimacteric (92 DAH) banana fruit pulp. Quantification of relative transcript levels by densitometry (Fig. 9).
(TIF)

Table S1 **MADS-box proteins from different plant species used in** **Table 1** **.**
(PDF)

Table S2 **MADS-box protein from various plant species used for the construction of phylogenetic tree (Figure S3).**
(PDF)

Table S3 **Primer sequences used for cloning of full-length and partial** ***MA-MADS5*** **cDNA (Table S3A), gel mobility shift assay and ChIP assay (Table S3B), semi quantitative RT-PCR (Table S3C) and cloning of promoter-reporter constructs,** ***GUS*** **(Table S3D), respectively.**
(PDF)

Text S1 **Supplementary Materials and Methods.**
(PDF)

Text S2 **Supplementary Results.**
(PDF)
