# Correction: Characterization of an AGAMOUS-like MADS Box Protein, a Probable Constituent of Flowering and Fruit Ripening Regulatory System in Banana

**DOI:** 10.1371/journal.pone.0357355

**Published:** 2026-09-01

**Authors:** Swarup Roy Choudhury, Sujit Roy, Anish Nag, Sanjay Kumar Singh, Dibyendu N. Sengupta

After this article [1] was published, concerns were raised regarding Fig 6. Specifically, panels I-IV in Fig 6F appear similar to panels I-IV in Fig 6G.

The corresponding author stated that panels I-IV in Fig 6F are incorrect and provided a corrected version of Figure 6 including replacement image panels for 6F that were acquired in the original experiment. The available underlying images for all panels of Figs 6F and 6G have been included with this notice as S1 File.

The original underlying data to support all results in the article and Supporting Information files are available from the corresponding author, except for the RT-PCR data supporting Fig 9 which are no longer available.

**Fig 6:**
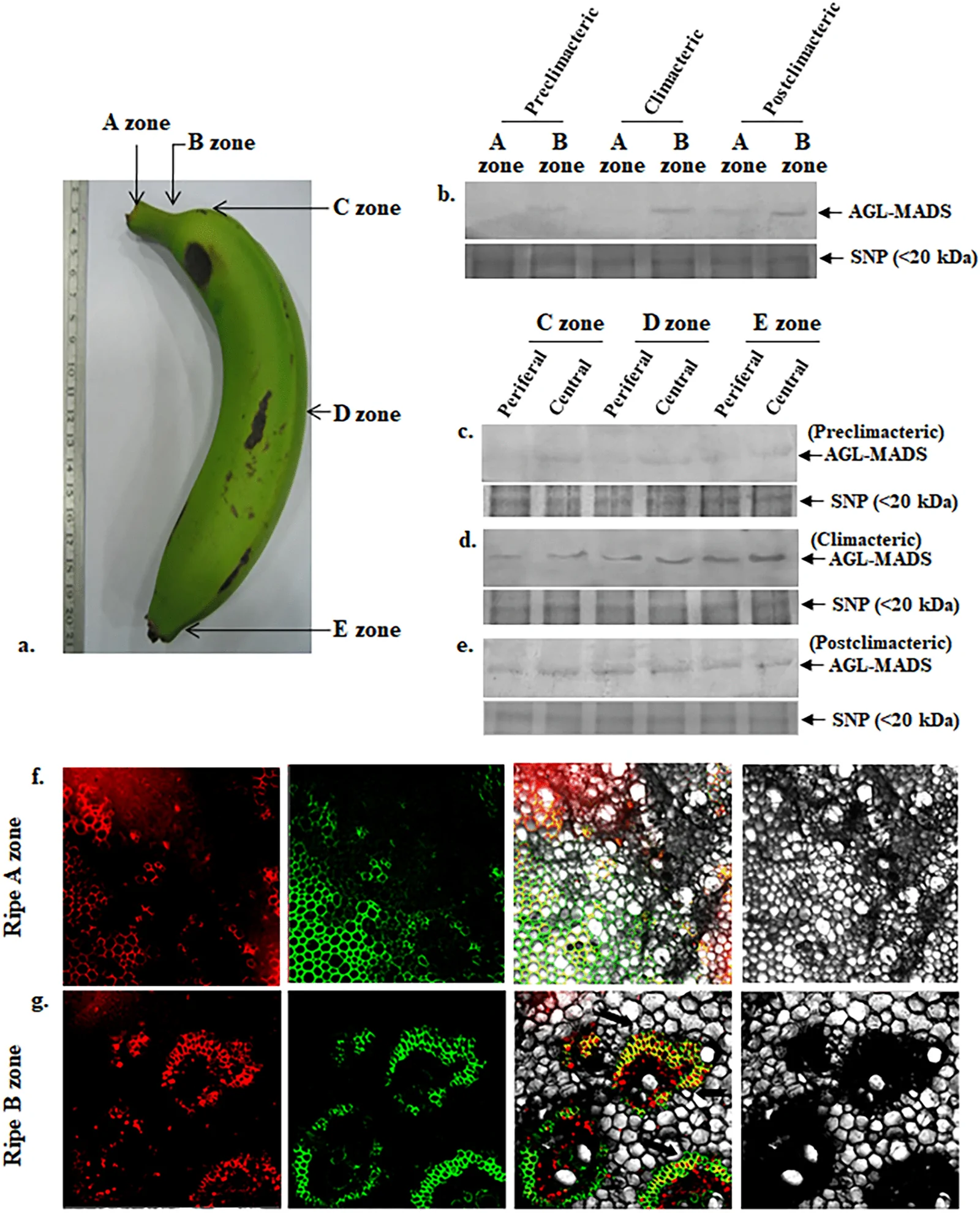
Analysis of expression of MA-MADS5 protein in various regions of banana fruit tissue. A Various regions of banana fruit from the cultivar Giant governor (*Musa sp*, AAA group, subgroup Cavendish) have been indicated (zones A–E). **b** Detection of MA-MADS5 protein in A and B zones of preclimacteric (0 DAH), climacteric (88 DAH) and postclimacteric (92 DAH) banana fruit by immunoblotting using anti-MA-MADS5 polyclonal antibody (1∶1000 dilution) (upper panel). Equal amounts of small nuclear protein was loaded in each lane and shown as loading control (lower panel). **c–e** Immunodetection of MA-MADS5 protein levels in C, D and E zones (including both peripheral and central parts) of preclimacteric, climacteric and postclimacteric fruit pulp tissues of banana (upper panel). Equal amounts of small nuclear protein was loaded in each lane and shown as loading control (lower panels of c, d and e respectively). Representative images from at least three independent experiments are shown for Figure b–e. **f and g** Immunolocalization of MA-MADS5 protein in A and B zones in postclimacteric banana pulp tissues (92 DAH). The legends of the panels I–IV are similar as described in Figure 5 b–e.

## Supporting information

S1 File Original images underlying all panels in Fig 6F and 6G. (ZIP)
