# Supplementary figures and images for: Characterization of an AGAMOUS-like MADS Box Protein, a Probable Constituent of Flowering and Fruit Ripening Regulatory System in Banana
Source: PLoS One. 2012 Sep 11;7(9):e44361. doi: 10.1371/journal.pone.0044361 (PMC3439491; doi:10.1371/journal.pone.0044361)

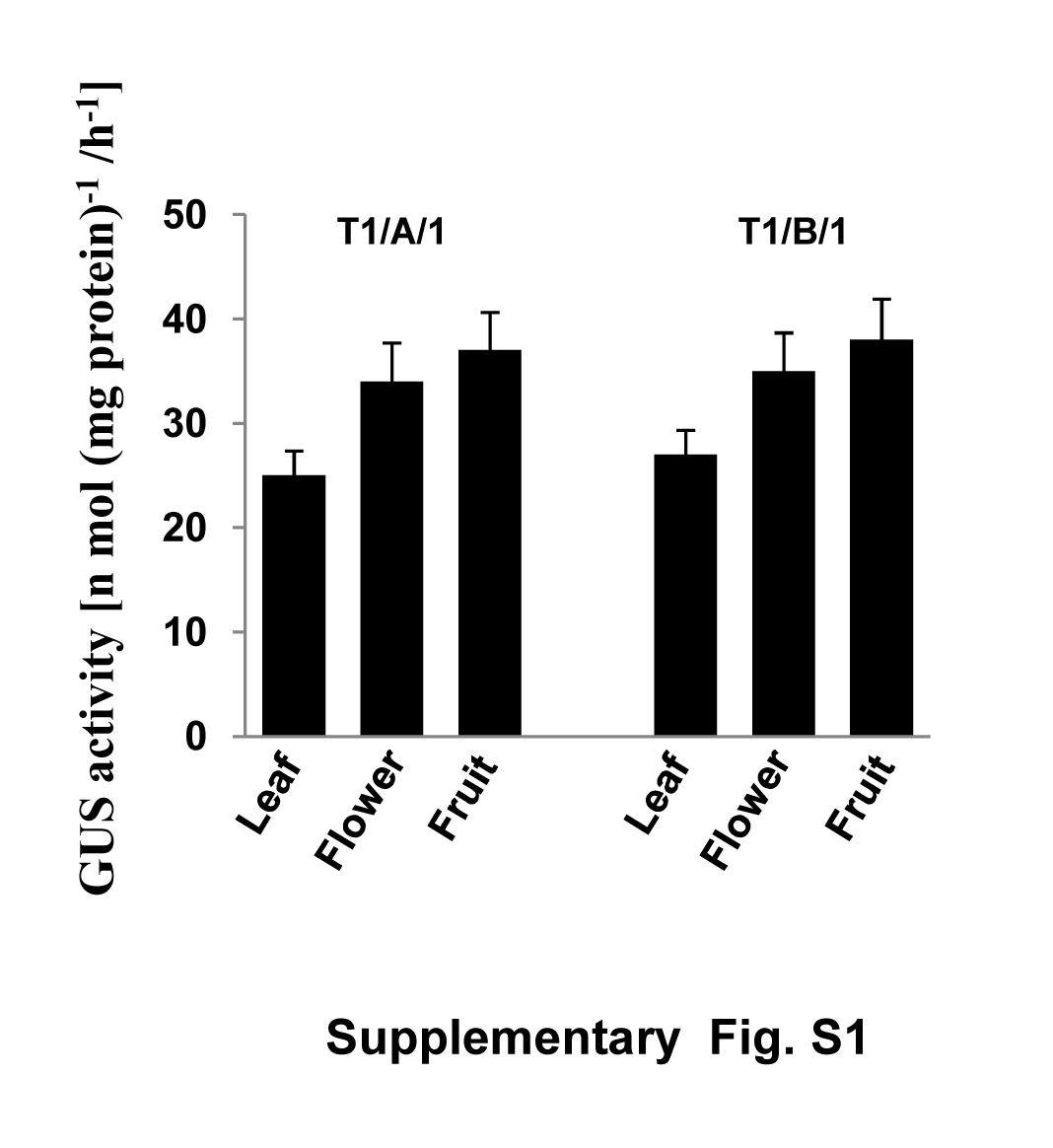

Supplement: Figure S1 — Detection of GUS activity in different tissues of transgenic tobacco lines. Measurement of GUS activity in the leaf, flower and fruit tissues of transgenic tobacco lines carrying the trimeric version of Arabidopsis Agamous MADS box binding element (3XCArG:GUS construct) in fusion with the GUS reporter gene. (TIF) [file pone.0044361.s001.tif]

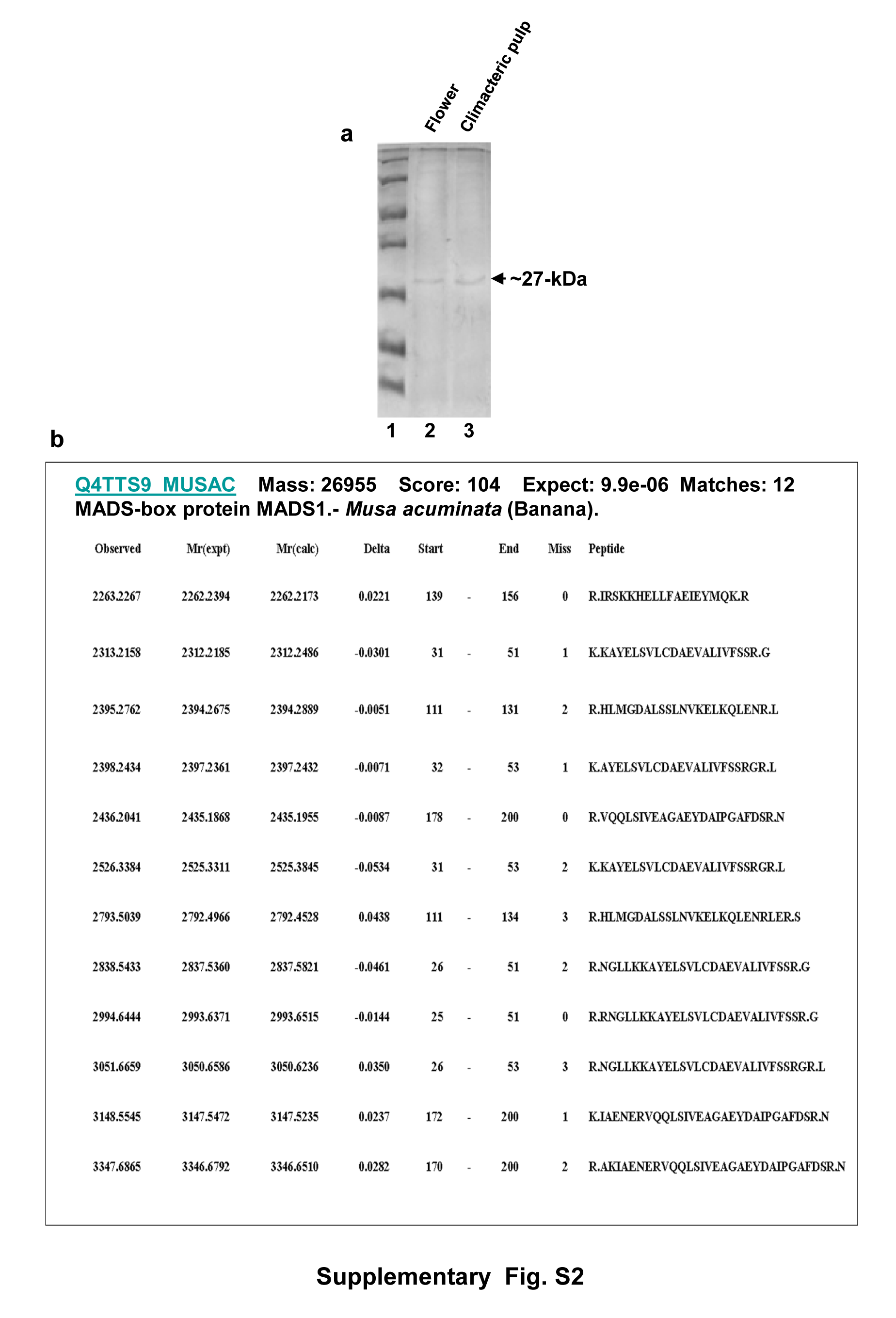

Supplement: Figure S2 — Identification of 27-kDa banana Agamous MADS box element binding protein from climacteric pulp tissue by mass spectrometry. a Eluted protein fractions from DNA-protein complexes of dried EMSA gel (flower, lane 2 and climacteric fruit pulp, lane 3) were concentrated, desalted and then resolved in 10% SDS-PAGE followed by staining of the gel with silver salt. Arrow indicates the position of 27-kDa single protein on gel. b Observed and expected monoisotopic [M+H]+ masses of ions from the tryptic digest of the Agamous MADS box element binding protein. (TIF) [file pone.0044361.s002.tif]

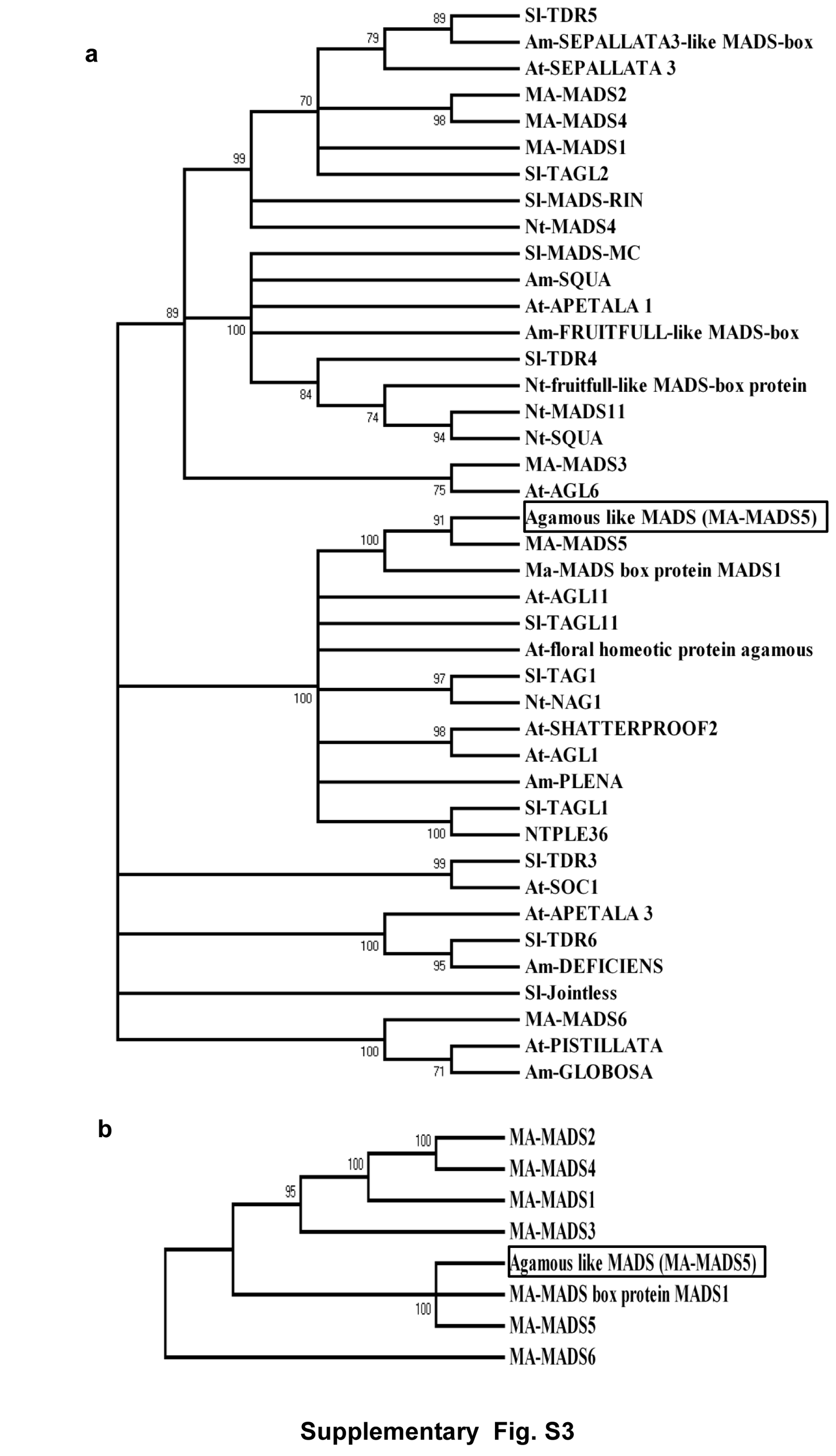

Supplement: Figure S3 — Phylogenetic analysis of Musa acuminata 27-kDa agamous MADS box element binding protein. a A consensus bootstrap neighbour-joining tree based on the CLUSTALW alignments of the amino acid sequences of MADS box from different organisms has been constructed by MEGA version 4.0. The sequences which have been used for phylogenetic tree formation have been summarized in Table S2. b A consensus bootstrap neighbour-joining tree based on the CLUSTALW alignments of the amino acid sequences of banana MADS box has been constructed by MEGA version 4.0. (TIF) [file pone.0044361.s003.tif]

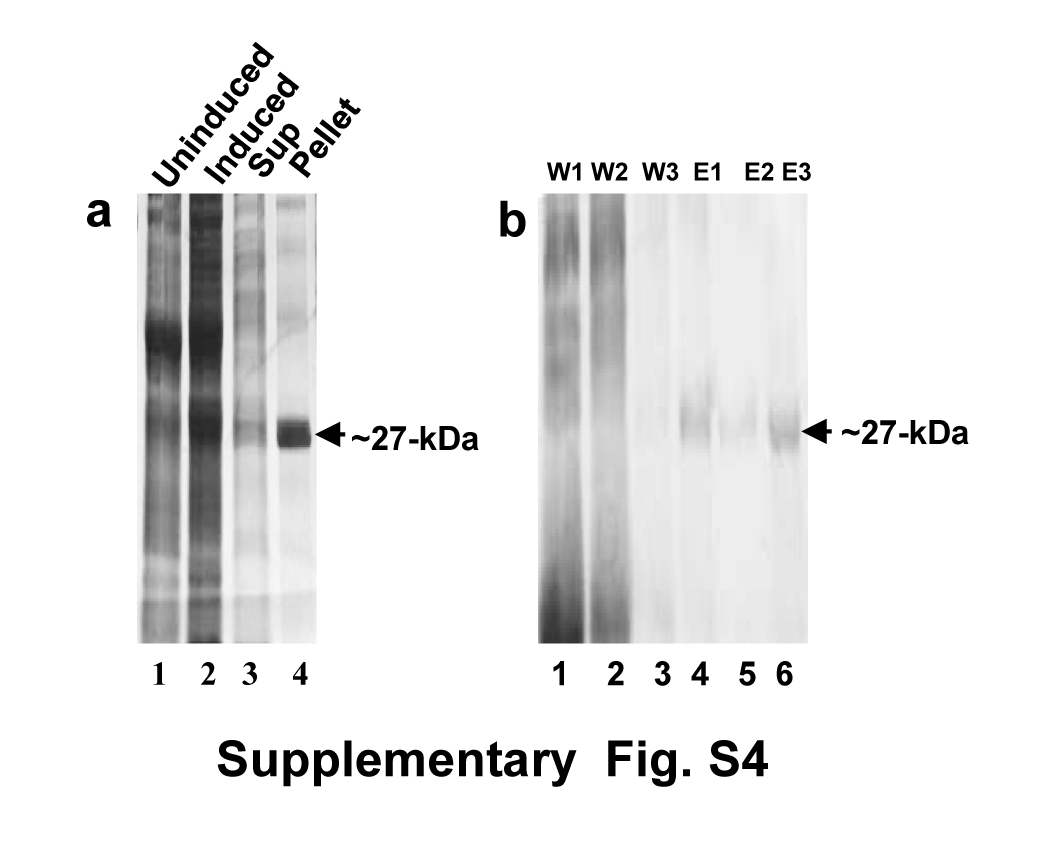

Supplement: Figure S4 — Over-expression of full-length MA-MADS5 protein in E.coli . a Separation of noninduced control (lane 1) and IPTG induced (lane 2) total protein extracts, obtained from M15 (Prep4) E.coli cells by 12% SDS-PAGE and stained with coomassie blue. After cell lysis small amount of recombinant protein remained in soluble fraction (lane 3), while considerable amount of recombinant protein remained insoluble fraction (lane 4). b The soluble fraction was further purified by Ni-NTA chromatography. Several wash fractions (lanes 1–3) and elution fractions (lanes 4–6) were detected. Arrow indicating the position of recombinant His-tagged MA-MADS5 protein on the gel. (TIF) [file pone.0044361.s004.tif]

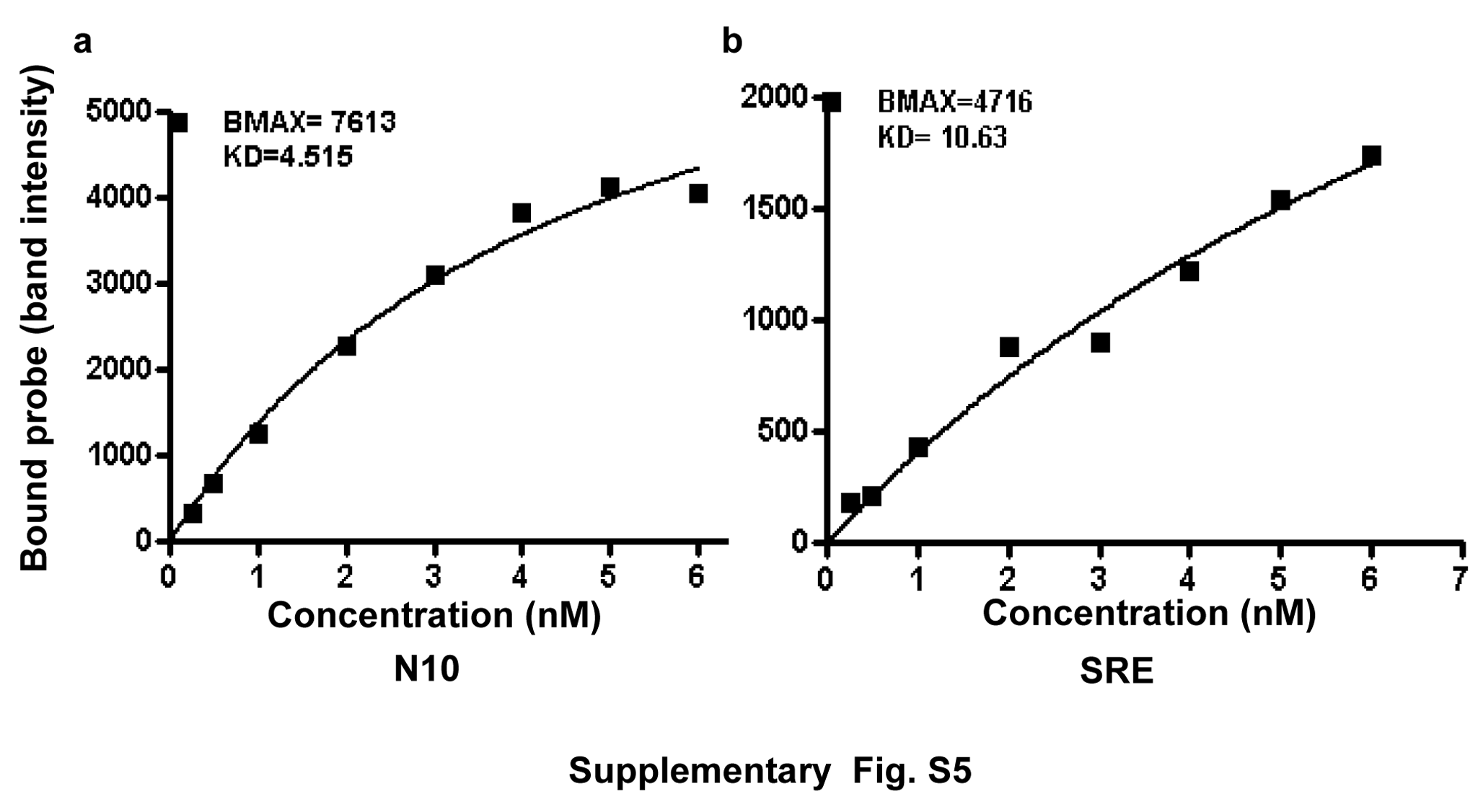

Supplement: Figure S5 — Detection of relative binding ability of MA-MADS5 protein to N10 and SRE DNA probe. Saturation binding assay in case of N10 and SRE were carried out by incubating a fixed amount of wild type recombinant proteins (MA-MADS5) with increasing amounts of radiolabeled N10 and SRE. The apparent BMAX and KD values were created in each case by Graph Pad Prism v4.0. (TIF) [file pone.0044361.s005.tif]

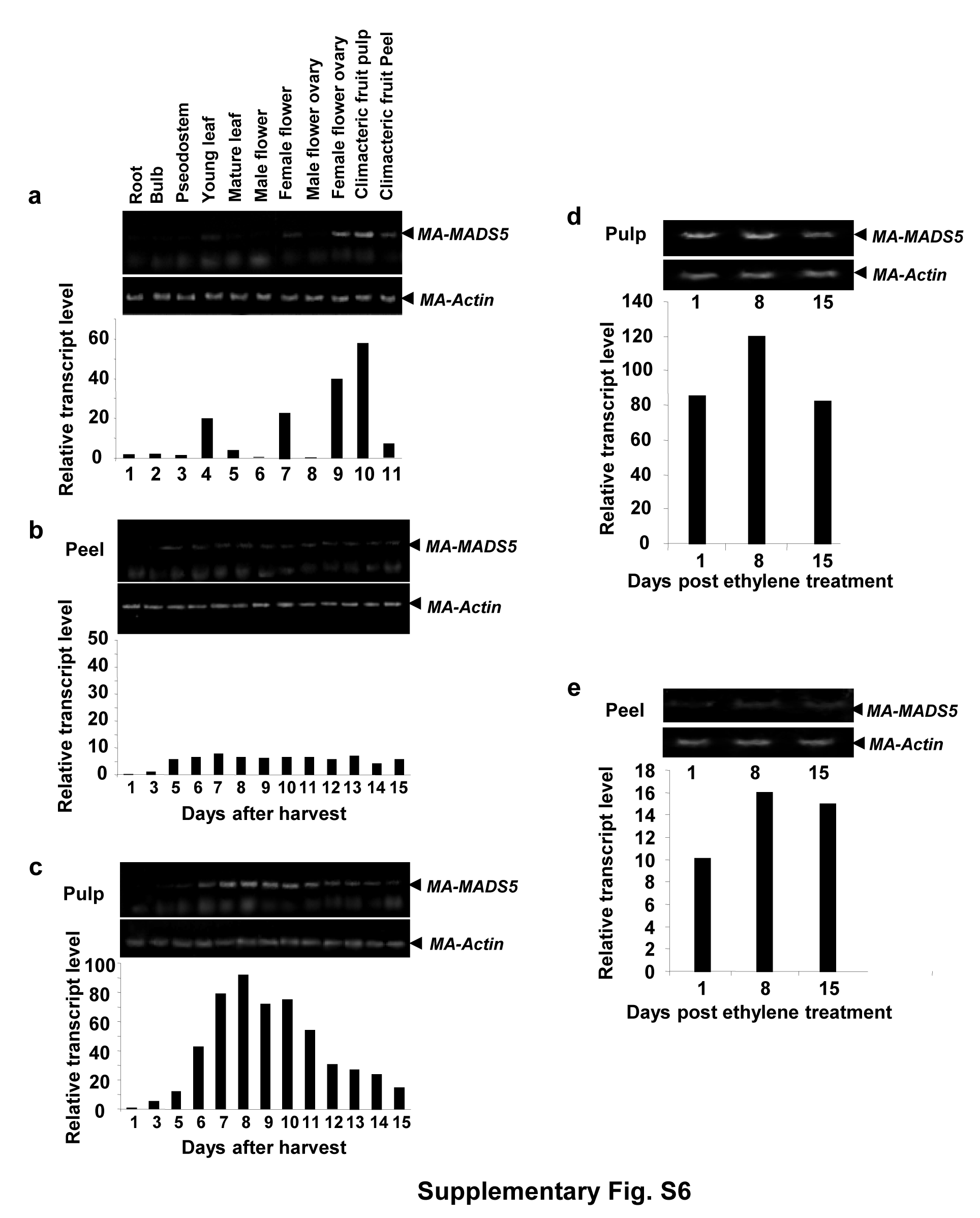

Supplement: Figure S6 — Transcript accumulation profiles of MA-MADS5 . a Transcript accumulation profiles of MA-MADS5 in different tissues of banana (cultivar giant governor) by semi quantitative RT-PCR. Transcript profile of MA-Actin was used as internal control in middle panel. Quantification of data in (a) by densitometry (Bio-Rad Image Densitometer) (lower panel). b and c. Changes in accumulation pattern of MA-MADS5 mRNA in the peel and pulp tissues during ripening in banana fruit ripened naturally at 25°C both. Transcript accumulation profiles for both the tissues were analyzed by semi quantitative RT-PCR with gene-specific primers. Transcript level of MA-Actin was used as internal control (middle panel). Transcript abundance of MA-MADS5 in each lane was detected by densitometry (Bio-Rad Image Densitometer) (lower panel). d and e Changes in the abundance of MA-MADS5 mRNA in the pulp and peel tissues of banana following ethylene treatment. Transcript abundance of MA-Actin was measured as internal control (middle panel). Relative transcript level of MA-MADS5 was detected by densitometry (lower panel). Representative gel images from three independent trials have been shown. (TIF) [file pone.0044361.s006.tif]

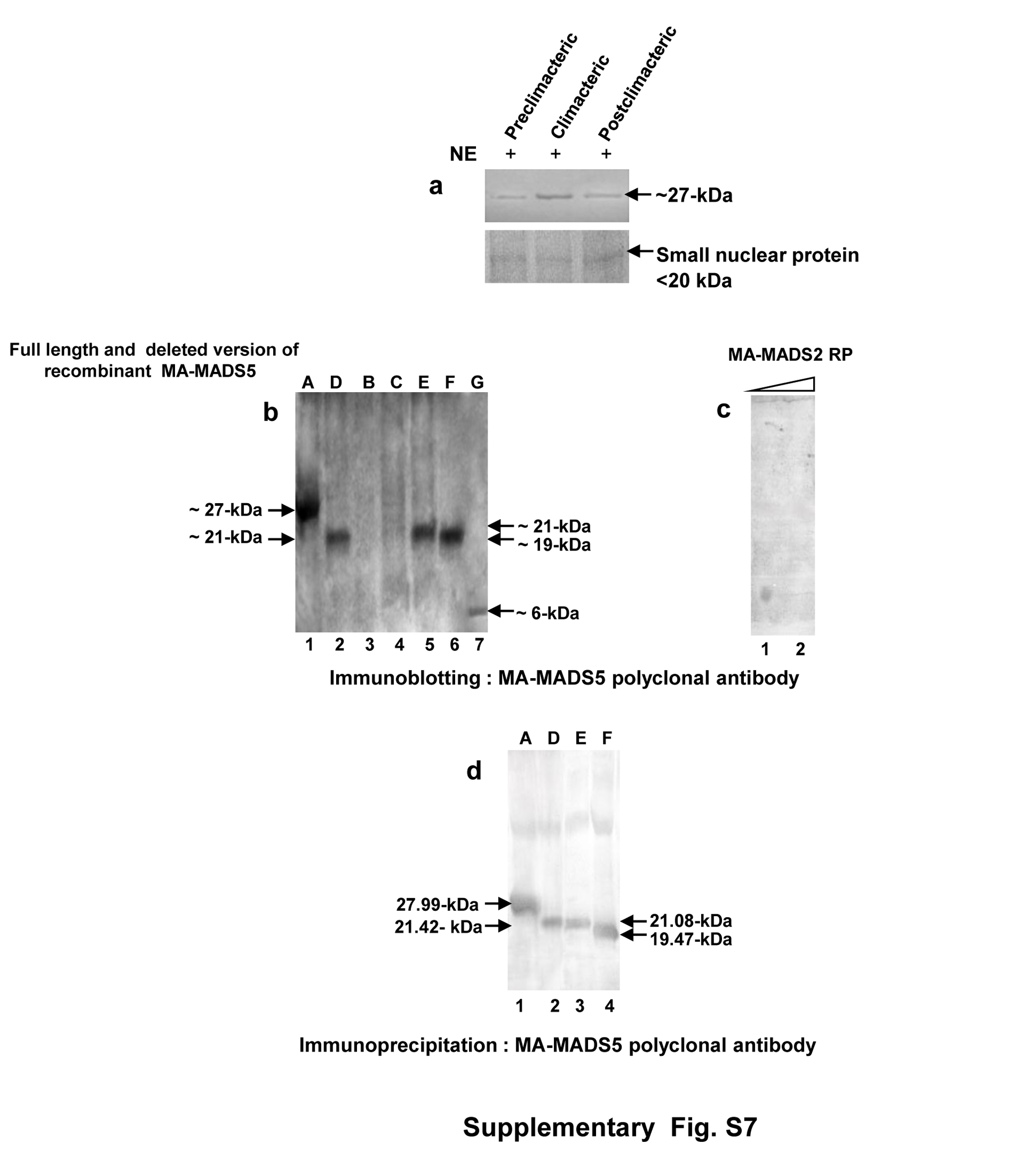

Supplement: Figure S7 — Detection of specificity of anti-MA-MADS5 polyclonal antibody. a Immunoblot analysis with nuclear protein extracts isolated from preclimacteric, climacteric and postclimacteric banana fruit pulp (lanes 1–3). ∼25 µg of nuclear extract was loaded in each lane and then immunoblotting was performed using anti-MA-MADS5 polyclonal antibody. b Equal amounts (2 µg) of purified wild type and deletion versions of MA-MADS5 recombinant proteins were loaded on 10% polyacrylamide gel and then immunoblotting was performed using anti-MA-MADS5 polyclonal antibody. c The banana MA-MADS2 protein was expressed in E. coli. The affinity purified (4 and 8 µg) recombinant proteins were resolved on 10% polyacrylamide gel and then immunoblotting was performed using anti-MA-MADS5 polyclonal antibody. d Equal amount (10 µg) of purified wild type and three deletion versions of MA-MADS5 recombinant proteins (A, D, E, F) were immunoprecipitated with anti-MA-MADS5 polyclonal antibody. The proteins in immunocomplex were subjected to immunoblotting using anti-MA-MADS5 polyclonal antibody. (TIF) [file pone.0044361.s007.tif]

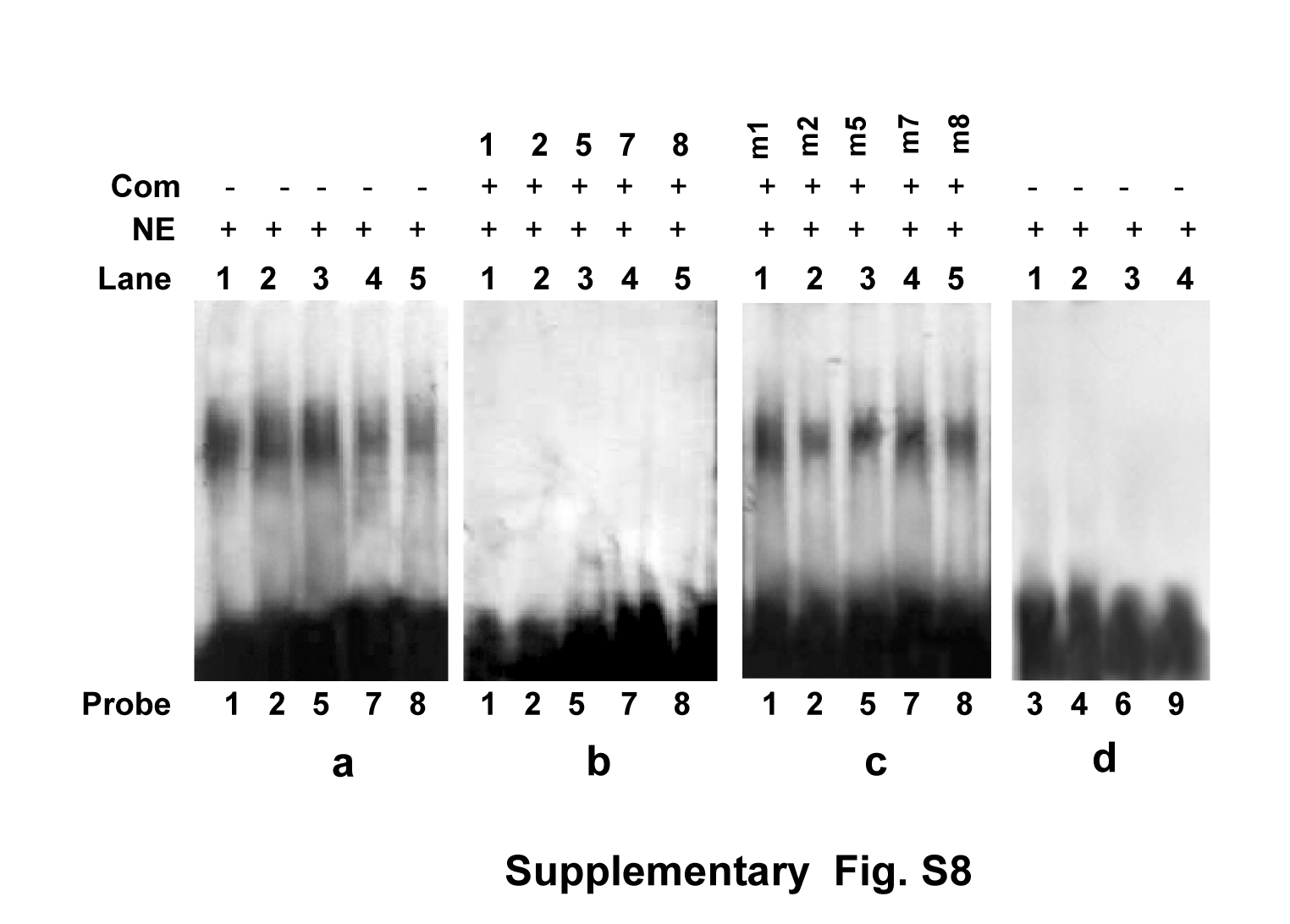

Supplement: Figure S8 — Gel mobility shift assay using CArG-box motifs detected in the promoter of different ripening specific genes. a 15 µg climacteric fruit pulp nuclear protein extract was incubated with the different CArG-box sequences, detected from various ripening gene promoters, as probes. The specific CArG-box motif which was used as probe indicated by numbers under the figure: CArG-box motif 1(lane 1), 2 (lane 2), 5 (lane 3), 7 (lane 4) and 8 (lane 4). b 15 µg climacteric fruit pulp nuclear protein extract was incubated with the different probes in presence of 100 molar excess of unlabeled same CArG-box motifs [1(lane 1), 2 (lane 2), 5 (lane 3), 7 (lane 4) and 8 (lane 4)] which were used as competitor. c In the similar gel mobility shift assay, 15 µg climacteric fruit pulp nuclear protein extract was incubated with the different probes in presence of 100 molar excess of unlabeled corresponding mutant CArG-box motifs which were used as competitor. d Gel mobility shift assay using different CArG-box motifs 3 (lane 1), 4 (lane 2), 6 (lane 3) and 9 (lane 4) used as probe. 15 µg climacteric fruit pulp nuclear protein extract was loaded in each lane. The specific CArG-box which was used as probe, has been indicated by numbers under the figure. (TIF) [file pone.0044361.s008.tif]

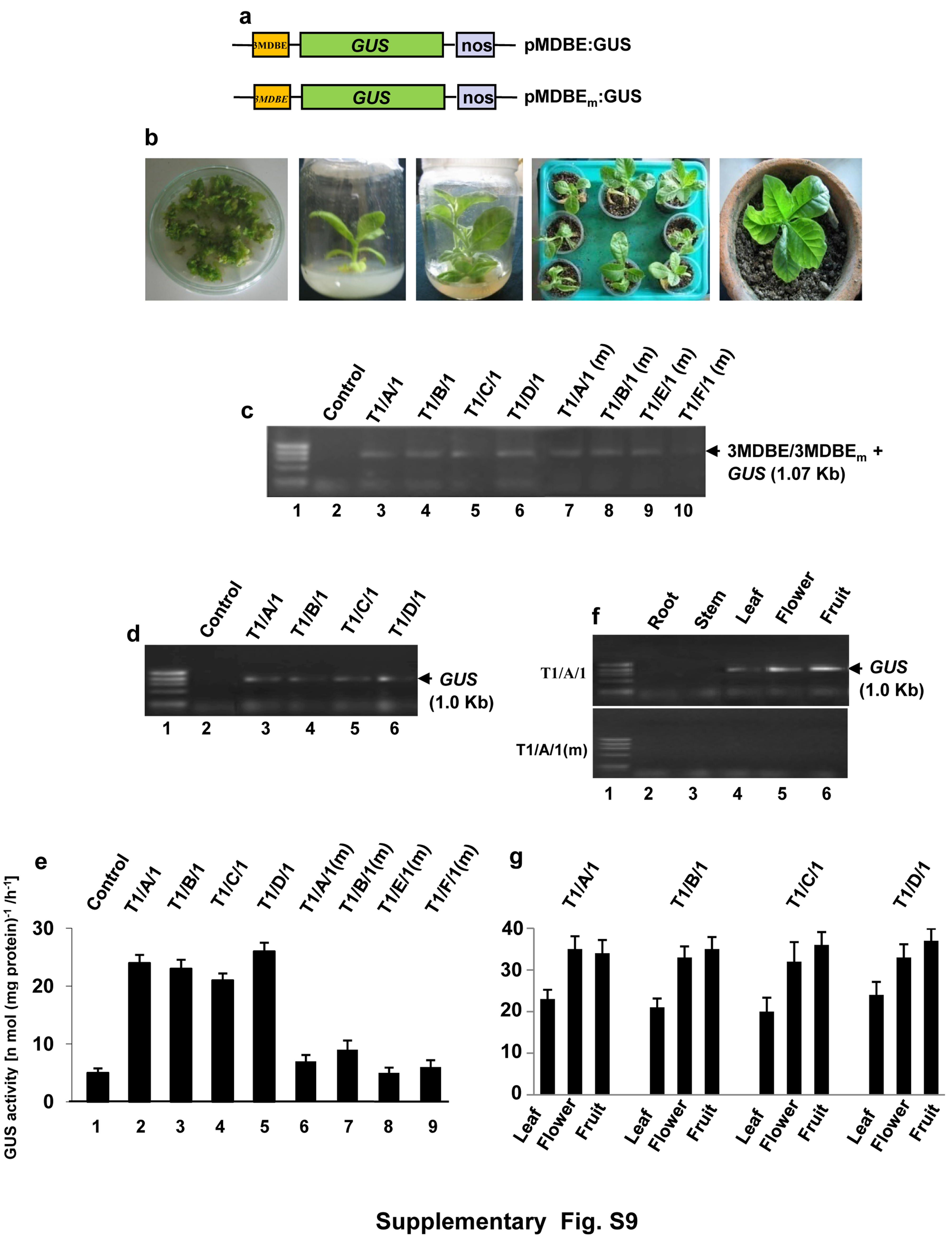

Supplement: Figure S9 — Analysis of activity of trimeric CArG box motif (3XCArG) of banana SPS promoter in transgenic tobacco lines. a The trimeric wild type and mutant CArG box motifs (pMDBE:GUS, pMDBEm:GUS) of banana SPS promoter fragment were fused to GUS reporter. b Stages of regeneration of transgenic tobacco plants after infection of tobacco leaf disc with Agrobacterium containing pMDBE:GUS constructs; Regeneration, shooting and rooting, hardening, mature stage in pot containing soil. c The integration of transgenes in transgenic tobacco lines were detected by genomic PCR with CArG box motif specific forward primer (70 UF) and GUS specific reverse primer (Table S3D). d Detection of GUS transcript level by semi quantitative RT-PCR in control (lane 2) and transgenic tobacco lines carrying trimeric wild type CArG box motif (lanes 3–6). Lane 1 indicates DNA molecular weight marker. e Detection of GUS activity in the leaves of control and transgenic tobacco lines carrying pMDBE:GUS or pMDBEm:GUS constructs. f Analysis of expression pattern of GUS transcript in different tissues of transgenic tobacco lines with wild type CArG-box motif (pMDBE:GUS) (upper panel, lanes 2–6) and mutant CArG box motif (pMDBEm:GUS) containing transgenic lines (lower panel, lanes 2–6) by semi quantitative RT-PCR. Lane 1 indicates DNA molecular weight marker. g GUS activity was detected in the leaf, flower and fruit tissues of transgenic tobacco lines carrying trimeric wild type CArG box motif. (TIF) [file pone.0044361.s009.tif]

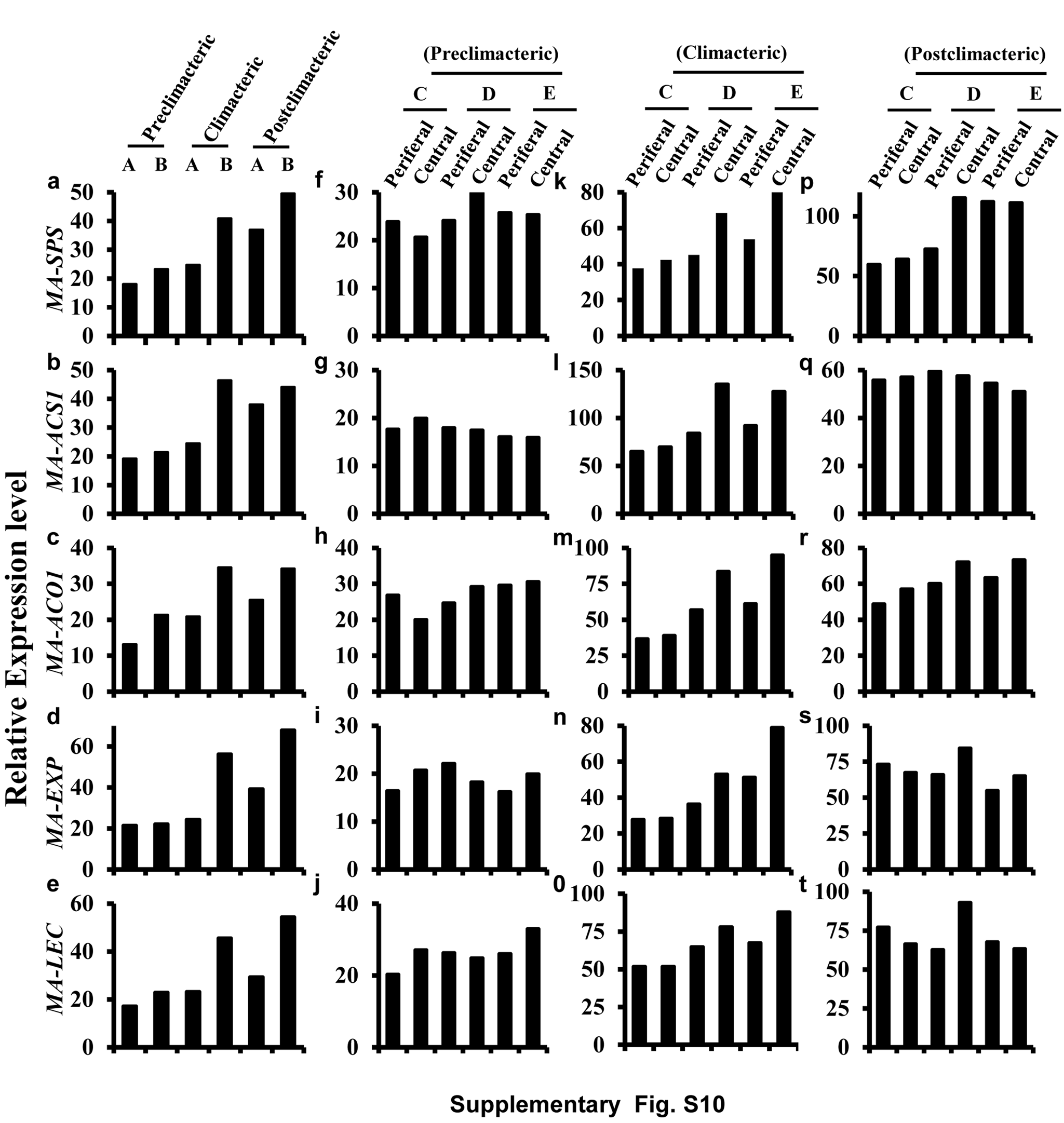

Supplement: Figure S10 — Transcript accumulation profiles major ripening genes in different zones of banana fruit. a-e Semi-quantitative RT-PCR analysis was carried out to detect the changes in transcript levels of MA-SPS, MA-ACS1, MA-ACO1, MA-EXP and MA-LEC in A and B zones of preclimacteric, climacteric and postclimacteric banana fruit. Transcript levels of MA-SPS, MA-ACS1, MA-ACO1, MA-EXP and MA-LEC ripening genes from peripheral and central regions of C, D and E zones of (f–j) preclimacteric (0 DAH), (k–o) climacteric (88 DAH) and (p–t) postclimacteric (92 DAH) banana fruit pulp. Quantification of relative transcript levels by densitometry (Fig. 9). (TIF) [file pone.0044361.s010.tif]
