# Supplementary material for: Characterization of an AGAMOUS-like MADS Box Protein, a Probable Constituent of Flowering and Fruit Ripening Regulatory System in Banana
Source: PLoS One. 2012 Sep 11;7(9):e44361. doi: 10.1371/journal.pone.0044361 (PMC3439491; doi:10.1371/journal.pone.0044361)
Supplement: Table S1 — MADS-box proteins from different plant species used in Table 1 . (PDF) [file pone.0044361.s011.pdf]

Table S1: MADS-box proteins from different plant species used in Table 1

Gene Name	Source	GeneBank Accession
**Agamous like MADS (MA-MADS5)**	*Musa acuminata*	**HQ730892**
**MA-MADS5**	*Musa acuminata*	**ACJ64682**
**SHATTERPROOF 2 (SHP2)**	*Arabidopsis thaliana*	**NP_850377**
**SEEDSTICK (STK)**	*Arabidopsis thaliana*	**NP_192734**
**AGAMOUS (AG)**	*Arabidopsis thaliana*	**NP_567569**
**MADS-box protein 2 (MADS2)**	*Lilium longiflorum*	**AAS01766**
**SEEDSTICK-like protein**	*Dendrobium thyrsiflorum*	**AAY86365**
**MADS-box**	*Asparagus virgatus*	**BAD83772**
**AGAMOUS-LIKE 6 (AGL6)**	*Arabidopsis thaliana*	**NP_182089**
**SEPALLATA 4**	*Arabidopsis thaliana*	**NP_973411**
**AGAMOUS-LIKE 8 (AGL8)**	*Arabidopsis thaliana*	**AAL66878**
