# Supplementary material for: Characterization of an AGAMOUS-like MADS Box Protein, a Probable Constituent of Flowering and Fruit Ripening Regulatory System in Banana
Source: PLoS One. 2012 Sep 11;7(9):e44361. doi: 10.1371/journal.pone.0044361 (PMC3439491; doi:10.1371/journal.pone.0044361)
Supplement: Table S2 — MADS-box protein from various plant species used for the construction of phylogenetic tree (Figure S3). (PDF) [file pone.0044361.s012.pdf]

Table S2: MADS-box protein from various plant species used for the construction of phylogenetic tree (Figure S3)

Gene Name	Source	GeneBank Accession
Ma-MADS-box protein MADS1	*Musa acuminata*	AAY53908
Agamous like MADS (MA-MADS5)	*Musa acuminata*	ADW08393
MA-MADS1	*Musa acuminata*	ACJ64679
MA-MADS2	*Musa acuminata*	ACJ64678
MA-MADS3	*Musa acuminata*	ACJ64680
MA-MADS4	*Musa acuminata*	ACJ64681
MA-MADS5	*Musa acuminata*	ACJ64682
MA-MADS6	*Musa acuminata*	ACJ64683
Sl-Jointless	*Solanum lycopersicum*	AAG09811
Sl-TAG1	*Solanum lycopersicum*	AAA34197
Sl-TAGL1	*Solanum lycopersicum*	AAM33101
Sl-TAGL2	*Solanum lycopersicum*	AAM33104
Sl-TAGL11	*Solanum lycopersicum*	AAM33102
Sl-TDR6	*Solanum lycopersicum*	AAM33100
Sl-TDR4	*Solanum lycopersicum*	AAM33098
Sl-TDR5	*Solanum lycopersicum*	CAA43170
Sl-TDR3	*Solanum lycopersicum*	CAA43168
Sl-MADS-MC	*Solanum lycopersicum*	AAM15774
Sl-MADS-RIN	*Solanum lycopersicum*	AAM15775
At-SEPALLATA 3	*Arabidopsis thaliana*	NP_564214
At-AGL6	*Arabidopsis thaliana*	NP_182089
At-SOC1	*Arabidopsis thaliana*	NP_182090
At- APETALA 1	*Arabidopsis thaliana*	NP_177074
At-APETALA 3	*Arabidopsis thaliana*	NP_191002
At-PISTILLATA	*Arabidopsis thaliana*	NP_197524
At-AGL11	*Arabidopsis thaliana*	NP_001078364
At-SHATTERPROOF2	*Arabidopsis thaliana*	AAU82079
At-floral homeotic protein agamous	*Arabidopsis thaliana*	CAA16753
At-AGL1	*Arabidopsis thaliana*	NP_191437
Am-PLENA	*Antirrhinum majus*	BAI68391
Am-GLOBOSA	*Antirrhinum majus*	BAI68390
EFICIENS	*Antirrhinum majus*	BAI68389
Am-FRUITFULL-like MADS-box	*Antirrhinum majus*	AAP83363
Am-SQUA	*Antirrhinum majus*	CAA45228
Am-SEPALLATA3-like MADS-box	*Antirrhinum majus*	AAP83366
Nt-NAG1	*Nicotiana tabacum*	AAA17033

Nt-MADS4	*Nicotiana tabacum*	AAF76381
Nt-MADS11	*Nicotiana tabacum*	AAO12211
Nt-fruitfull-like MADS-box protein	*Nicotiana tabacum*	ABF82231
Nt-SQUA	*Nicotiana tabacum*	AAD09496
NTPLE36	*Nicotiana tabacum*	AAD09499
