# Supplementary material for: Characterization of an AGAMOUS-like MADS Box Protein, a Probable Constituent of Flowering and Fruit Ripening Regulatory System in Banana
Source: PLoS One. 2012 Sep 11;7(9):e44361. doi: 10.1371/journal.pone.0044361 (PMC3439491; doi:10.1371/journal.pone.0044361)
Supplement: Table S3 — Primer sequences used for cloning of full-length and partial MA-MADS5 cDNA (Table S3A), gel mobility shift assay and ChIP assay (Table S3B), semi quantitative RT-PCR (Table S3C) and cloning of promoter-reporter constructs, GUS (Table S3D), respectively. (PDF) [file pone.0044361.s013.pdf]

**Table S3: Primer sequences used for cloning of full-length and partial *MA-MADS5* cDNA (Table S3A), gel mobility shift assay and ChIP assay (Table S3B), semi quantitative RT-PCR (Table S3C) and cloning of promoter-reporter constructs, *GUS* (Table S3D), respectively**

**Table S3A**

| | | |
|---|---|---|
| **1** | MD A | 5´ GAACGAGTGCAGCAACTGA 3´ |
| **2** | MD B | 5´ CGCCGCTGAATCCGCC 3´ |
| **3** | MD C | 5´ TCAGTTGCTGCACTCGTTC 3´ |
| **4** | MD F | 5´ GATCGGATCCATGGGAAGGGGTAAGATTGAGAT 3´ |
| **5** | MD R | 5´ GATCAAGCTTTCACGCCGCTGAATCCGCC 3´ |
| **6** | MD 60 R | 5´GATCAAGCTTGTCATTGGCGTACTCATATAGC 3´ |
| **7** | MD 73 R | 5´GATCAAGCTTGCATGCCTTCTTGTACCTCTC 3´ |
| **8** | MD 183 R | 5´GATCAAGCTTGTTCTCTGCTATCTTGGCTCTG 3´ |
| **9** | MD 61 F | 5´GATCGGATCCAACATAAAATCAACAATCGAGAG 3´ |
| **10** | MD 74 R | 5´GATCGGATCCGCTGACAGTTCGAGTTCGG 3´ |
| **11** | MD 184 R | 5´GATCGGATCCGAACGAGTGCAGCAACTGA 3´ |

**Table S3B**

| | | |
|---|---|---|
| **1** | AGAMOUS element 1 | 5´ACTGAAGC**TTTCCATTAATGGGAA**TGACGCACAA TCCCAC 3´ |
| **2** | AGAMOUS element m1 | 5´ACTGAAGC**TTTCCATGCATGGGAA**TGACGCACAA TCCCAC 3´ |
| **3** | 17LS | 5´GTGGGATTGTGCGTCAT 3´ |
| **4** | MA-SPS upstream F **1** | 5´ TTGGATT**CTTATATG**TCACGAA 3´ |
| **5** | MA-SPS upstream R **1** | 5´ TTCGTGA**CATATAAG**AATCCAA 3´ |
| **6** | MA-ACS1 upstream F **2** | 5´ACACCC**CATAAATTTG**ATCTTC 3´ |
| **7** | MA-ACS1 upstream R **2** | 5´GAAGAT**CAAATTTATG**GGGTGT 3´ |

| | | |
|---|---|---|
| **8** | MA-ACS1 upstream F **3** | 5´ GGGTCAA**CTATAATTG**CCTTCC3´ |
| **9** | MA-ACS1 upstream R **3** | 5´ GGAAGG**CAATTATAG**TTGACCC 3´ |
| **10** | MA-ACS1 upstream F **4** | 5´GATCATG**CTTTTATTG**TTTCTT 3´ |
| **11** | MA-ACS1 upstream R **4** | 5´AAGAAA**CAATAAAAG**CATGATC 3´ |
| **12** | MA-ACO1 upstream F **5** | 5´GAGGTTGG**CAATTTG**TCACTAC 3´ |
| **13** | MA-ACO1 upstream R **5** | 5´GTAGTGA**CAAATTG**CCAACCTC 3´ |
| **14** | MA-ACO1 upstream F **6** | 5´GAACGC**CTATAAATTG**CCTGGT 3´ |
| **15** | MA-ACO1 upstream R **6** | 5´ACCAGG**CAATTTATAG**GCGTTC 3´ |
| **16** | MA-EXP upstream F **7** | 5´AAGCACG**CATATTTG**TGCTGCA 3´ |
| **17** | MA-EXP upstream R **7** | 5´ TGCAGCA**CAAATATG**CGTGCTT 3 |
| **18** | MA-LEC upstream F **8** | 5´ CTGGTAT**CTATTTATG**AAATCT 3´ |
| **19** | MA-LEC upstream R **8** | 5´AGATTT**CATAAATAG**ATACCAG 3´ |
| **20** | MA-LEC upstream F **9** | 5´ ATAAATT**CTTAAAAG**ATCCTAG3´ |
| **21** | MA-LEC upstream R **9** | 5´ CTAGGAT**CTTTTAAG**AATTTAT 3´ |
| **22** | MA-SPS upstream F m**1** | 5´ TTGGATT**CTTGCATG**TCACGAA 3´ |
| **23** | MA-SPS upstream R m**1** | 5´ TTCGTGA**CATGCAAG**AATCCAA 3´ |
| **24** | MA-ACS1 upstream F m**2** | 5´ACACCC**CAGCAATTTG**ATCTTC 3´ |
| **25** | MA-ACS1 upstream R m**2** | 5´GAAGAT**CAAATTGCTG**GGGTGT 3´ |
| **26** | MA-ACO1 upstream F m**5** | 5´GAGGTTGG**TGATTTG**TCACTAC 3´ |
| **27** | MA-ACO1 upstream R m**5** | 5´GTAGTGA**CAAATCA**CCAACCTC 3´ |
| **28** | MA-EXP upstream F m**7** | 5´AAGCACG**CATATCGG**TGCTGCA 3´ |
| **29** | MA-EXP upstream R m**7** | 5´ TGCAGCA**CCGATATG**CGTGCTT 3´ |
| **30** | MA-LEC upstream F m**8** | 5´ CTGGTAT**CTGTTCATG**AAATCT 3´ |
| **31** | MA-LEC upstream R m**8** | 5´AGATTT**CATGAACAG**ATACCAG 3´ |
| **32** | GATA box F | 5´ CGCG**GATAAG**CGTACGCG**GATAAG**CGTA 3´ |
| **33** | GATA box R | 5´ TACG**CTTATC**CGCGTACG**CTTATC**CGCG 3´ |

| | | |
|---|---|---|
| **34** | 1CP F | F 5´ ATCTGACGCTTAACGTACGC 3´ |
| **35** | 1CP R | 5´ CGCTCTTGGGAATCAGCCGG 3´ |
| **36** | 2CP F | 5´ TTGATGGATTGCTGGAAATG 3´ |
| **37** | 2CP R | 5´ TTAAATAAATTATTAGGTGA 3´ |
| **38** | 3CP F | 5´ GACC CCATCGATATTAGATT 3´ |
| **39** | 3CP R | 5´ GTTGGAAGGCAATTATAGTT 3´ |
| **40** | 4CP F | F 5´ CCTACAAACAACACCAATCC 3´ |
| **41** | 4CP R | 5´ CCTTTGCCTAATTGGATTTT 3´ |
| **42** | 5CP F | 5´ TTTCTACCTATCGATCCCTG 3´ |
| **43** | 5CP R | 5´ TGCTTCCATCGGTCTCTCCC 3´ |
| **44** | 6CP F | 5´ TTATGGAATCGGCTCCGTTA 3´ |
| **45** | 7CP F | 5´ TGTTGTTTATTAGCGTACTG 3´ |
| **46** | 7CP R | 5´ GACTCACCCTTTGAAGGCTG 3´ |
| **47** | 8CPF | 5´ TTGACTATACAATTTCACTC 3´ |
| **48** | 8CPR | 5´ GATGGTGCAGATCGTAGATA 3´ |

**Table S3C**

| | | |
|---|---|---|
| **1** | MA-SPS F | 5´ TCGGATCCATGGCGGGAAACGACTGGAT 3´ |
| **2** | MA-SPS R | 5´ATGCGGATCCGCGGCATTCACCAAAAGCTTT 3´ |
| **3** | MA-ACS1 F | 5´GCATGAGCTCATAACGGGTCACATGAGGATCTAC 3´ |
| **4** | MA-ACS1 R | 5´GTTTCAGGTGGCGG CTTGAAC 3´ |
| **5** | MA-ACO1 F | 5´GATCGAATTCATGGATTCCTTTCCGGT 3´ |
| **6** | MA-ACO1 R | 5´ACGTGAATTCTTAAGAGGTAGCGATTGGGTG 3´ |
| **7** | MA-EXP 1 F | 5´GATCGGATCCATGGCTAAGTCAATCTCCTC 3´ |
| **8** | MA-EXP1 R | 5´GATCGAGCTCCTAGAATTGCTGTCCCTGG 3´ |
| **9** | MA-LEC F | 5´GATCGGATCCATGAACGGAGCGATCAAGGT 3´ |
| **10** | MA-LEC R | 5´GATCGAGCTCTTATGGCTCCAAGTAGACCC 3´ |

**Table S3D**

| | | |
|---|---|---|
| **1** | AGAMOUS element 1/1 | 5´ACTG*AAGCTTT*T**CCATTAATGG**GAATTT**CCATTAATGG**GAATTT**CCATTAATGGG**AATGACGCACAATCCCAC 3´ |
| **2** | AGAMOUS element m1 | 5´ACTG*AAGCTTT*T**CCATGCATGG**GAATTT**CCATGCATGG**GAATTT**CCATGCATGG**GAATGACGCACAATCCCAC 3´ |
| **3** | SPS UP MDBE element | 5´ACTG*AAGCTT*GATT**CTTATATG**TCACGATT**CTTATATGT**TCACGATT**CTTATATG**TCACATGACGCACAATCCCAC 3´ |
| **4** | SPS UP MDBE element m1 | 5´ACTG*AAGCTT*GATT**CTTGCATG**TCACGATT**CTTGCATG**TCACGATT**CTTGCATG**TCACATGACGCACAATCCCAC 3´ |
| **5** | 70 LS-CaMV basal promoter 1/1 | 5´AGGCTA*GGATCC*TCTAGATTCCCTCTCCAAATGAAATGAACTTCCTTATATAGAGGAAGGGTCTTGCGAAGGATAGTGGGATTGTGCGTCAT 3´ |
| **6** | 70UF | 5´ ATGACGCACAATCCCACTATCCTT 3´ |
| **7** | GUS F | 5´CAACGTCTGCTATCAGCGCGAAGT 3´ |
| **8** | GUS R | 5´ TATCCGGTTCGTTGGCAATACTCC 3´ |
