# Supplementary material for: Characterization of an AGAMOUS-like MADS Box Protein, a Probable Constituent of Flowering and Fruit Ripening Regulatory System in Banana
Source: PLoS One. 2012 Sep 11;7(9):e44361. doi: 10.1371/journal.pone.0044361 (PMC3439491; doi:10.1371/journal.pone.0044361)
Supplement: Text S1 — Supplementary Materials and Methods. (PDF) [file pone.0044361.s014.pdf]

Supplementary Materials and Methods

Detection of apparent KD values for DNA binding study

The plasmid pAS152 was constructed by ligating the two annealed phosphorylated oligonucleotides ADS165 (5′-CTAGGAGGAAAACTATTTATAGATCAAAT-3′) and ADS166 (5′-CTAGATTTGATCTATAAATAGTTTTCCTC-3′) into the *XbaI* site of pBEND2 [S1]. The oligonucleotide containing the N10 site has been underlined. pAS76 is an analogous construct based on pBEND2 and it contained *c-fos* SRE instead of N10 (central sequence, 5′-CCATATTAGG-3′) [27,39]. At first, after restriction digestion with *EcoRV*, the two DNA binding regions (N10 and SRE) were purified from native 10% polyacrylamide gel and then dephosphorylated by calf intestinal alkaline phosphatase (Roche, Germany). Dephosphorylated DNA fragments were then 5′-end-labeled using T4 polynucleotide kinase in the presence of [γ-^{32}P] ATP. DNA binding reactions were carried out essentially as described previously [29]. Saturation binding assay to detect the dissociation constants (KD) in case of N10 and SRE were carried out by incubating a fixed amount of wild type recombinant proteins (MA-MADS5) with increasing amounts of radiolabeled N10 and SRE under standard reaction conditions. Reaction products were resolved by electrophoresis in a 6% non-denaturing polyacrylamide gel and the amount of DNA-protein complex (bound probe) and free probe were calculated by densitometry (Bio Rad Imaging Densitometer, GS-700). The estimation of apparent BMAX and KD values were created in each case by Graph Pad Prism v4.0.

Immunoprecipitation

Immunoprecipitation of recombinant MA-MADS5 was carried out by essentially following the protocol described previously [S2]. Bacterially overexpressed recombinant proteins (~5 μg) were incubated with either anti-MA-MADS5 antibody (~3 μg of affinity purified IgG) or similar amounts of pre-immune serum (used as control) in presence of binding buffer (50 mM Hepes-KOH, pH 8.0; 10 mM $MgCl_2$, 1mM DTT, 1mM PMSF and 5 μg/ml Leupeptin) for 4 h at 4˚C with constant gentle rotation. The immunocomplex was adsorbed onto binding buffer equilibrated protein A agarose resin (Sigma). The mixture was then incubated for overnight at 4˚C with rotation. The immunocomplex was pelleted by centrifugation at 2,000 ×g for 10 min at 4˚C and then the pellet was dissolved in binding buffer containing protease inhibitors.

Semi quantitative RT-PCR of the ripening specific gene transcript

Total RNA was extracted from different regions of banana pulp tissues at different ripening stages by the methods described earlier [29]. Semi-quantitative RT-PCR was performed with first strand cDNA template using with Hot Start high fidelity Taq DNA polymerase (Qiagen). The *MA-MADS5*, *MA-SPS* (*Musa acuminata Sucrose Phosphate synthase*), *MA-ACS1* (*Musa acuminata 1-Amino cyclopropane 1-carboxylic acid synthase 1*), *MA-ACO1* (*Musa acuminata* 1-*Amino cyclopropane 1-carboxylic acid oxidase1*), *MA-EXP* (*Musa acuminata Expansin*) and *MA-LEC* (*Musa acuminata Lectin*) were amplified using the primers for the respective genes. The primers used for transcript analysis by semi-quantitative RT-PCR are listed in Table S3C. *MA-Actin* mRNA was used as endogenous control. For semi quantitative RT-PCR analysis of *MA-SPS*, a first cycle of 5 min at 94ºC, 45 s at 57ºC, and 1 min at 72ºC was followed by 45 s at 94ºC, 45 s at 57ºC and 1 min at 72ºC for 24 cycles. For *MA-ACS1*, a first cycle of 5 min at 94ºC, 45 s at 65ºC, and 1 min at 72ºC was followed by 45 s at 94ºC, 1 min at 65ºC and 1 min at 72ºC for 26 cycles. For *MA-ACO1*, a first cycle of 5 min at 94ºC, 45 s at 53ºC and 1 min at 72ºC was followed by 45 s at 94ºC, 45 s at 53ºC and 1 min at 72ºC for 25 cycles. For *MA-EXP*, a first cycle of 3 min at 94ºC, 45 s at 53ºC, and 1 min at 72ºC was followed by 45 s at 94ºC, 45 s at 53ºC and 1 min at 72ºC for 24 cycles. For *MA-LEC*, a first cycle of 3 min at 94ºC, 45 s at 55ºC and 1 min at 72ºC was followed by 45 s at 94ºC, 45 s at 55ºC and 1 min at 72ºC for 25 cycles. The conditions of RT-PCR were chosen so that none of the mRNAs selected for analysis reached a plateau at the end of the amplification protocol. We also performed a negative control containing RNA instead of cDNA to rule out genomic DNA contamination in each set of reactions. Equal amounts of PCR products were loaded on 1% agarose gel for transcript profile analysis. The transcript expression profiles of different ripening genes were normalized relative to the transcript levels of *MA-Actin*. Transcript abundance of *MA-Actin* measured by RT-PCR under specified conditions was used as loading control. The end products were quantified in-gel using Quantity One software (BioRad). Product quantity was normalized against *MA-Actin* products after background subtraction. Fold change was measured as the normalized product quantity for each gene transcript for specific tissue zone at particular stage of ripening /normalized product quantity for *MA-Actin*.

References

S1. Kim J, Zweib C, Wu C, Adhya S (1989) Bending of DNA by gene regulatory proteins: Construction and use of a DNA bending vector. Gene 85: 15-23.

S2. Roy S, Roy Choudhury S, Mukherjee SK, Sengupta DN. (2007) Tobacco PCNA interacts directly and stimulates both activity and processivity of mungbean DNA polymerase. Arch Biochem Biophys 468:22-31
