# Supplementary material for: Characterization of an AGAMOUS-like MADS Box Protein, a Probable Constituent of Flowering and Fruit Ripening Regulatory System in Banana
Source: PLoS One. 2012 Sep 11;7(9):e44361. doi: 10.1371/journal.pone.0044361 (PMC3439491; doi:10.1371/journal.pone.0044361)
Supplement: Text S2 — Supplementary Results. (PDF) [file pone.0044361.s015.pdf]

Supplementary Results

Differential DNA binding by MA-MADS5

To further look into the DNA binding properties of MA-MADS5, we next investigated the relative binding ability of MA-MADS5 to two different types of MADS-box protein binding sites: *c-fos* SRE and N10 [19,39]**.** We have used pAS76 (containing the SRE site) and pAS152 (containing the N10 site) for DNA binding analysis *in vitro*. Binding sites were chosen which represent natural binding sites for SRF (*c-fos* SRE) and MEF2 (N10-site). DNA-protein interaction assays using radiolabeled SRE or N10 with affinity resin purified full-length recombinant MA-MADS5 have revealed a comparatively weak DNA binding activity in case of SRE as compared to N10 (Figure S5a and b). The result also indicated that the affinity of protein towards the probe becomes constant after a certain probe concentration was reached. This can be explained by the fact that the protein starts to dissociate from the probe beyond a particular concentration of probe at which point protein-probe DNA interaction becomes saturated. In case of both N10 and SRE, initially a uniform increase in probe binding and as well as density were detected. However, in case of N10, DNA binding by MA-MADS5 appeared to saturate after 4 nM probe concentration. On the other hand, in case of SRE, probe binding increased steadily even after 5-6 nM concentration of the probe. Therefore, in case MA-MADS5-N10 interaction, saturation was achieved much earlier than with SRE and the maximum amount of complex formation was ~3 times higher in case of N10 than SRE, suggesting relatively higher affinity of MA-MADS5 towards N10 than SRE. For N10 site containing probe, BMAX value was higher (7613) than SRE (4716). The KD value was lower for N10 than SRE [the KD values were found to be as 4.515 nM and 10.63 nM for N10 and SRE respectively (Figure S5a and b)], indicating relatively higher affinity of MA-MADS5 for N10 than SRE. Student's t-test analysis was carried out to test the significance of difference in KD values using KD values obtained from N10 or SRE site binding in three independent trials.

Expression of *MA-MADS5* transcripts in peel and pulp during ripening

Previously we have characterized the ripening behaviour in Cavendish banana [25]. To understand possible role of *MA-MADS5* in fruit ripening, we have next examined the transcript expression levels of the gene in vegetative, reproductive and fruit tissues of this cultivar of

banana. *MA-MADS5* was found to be highly expressed in female flower ovary and fruit pulp at the climacteric phase (Figure S6a, lanes 9 and 10). In addition, expression of *MA-MADS5* transcripts was also detected in young leaf and female flower (Figure S6a, lanes 4 and 7). No significant expression of gene transcript was detected in other vegetative and reproductive tissues.

We next examined the changes in transcript expression levels of *MA-MADS5* in peel and pulp tissues during various ripening days after harvest. As shown in Figure S4b and c, an interesting pattern of expression of banana *MA-MADS5* was detected in peel and pulp tissues. In peel, expression of *MA-MADS5* was very low or only at detectable level during the initial phase of ripening. The transcript expression level increased only marginally at day 5 after harvest and then did not increase further along with the increase in ethylene production rate at climacteric stage. In contrast, in pulp *MA-MADS5* mRNA was very low at the preclimacteric stages (days 1-2 after harvest). It was then increased during onset of climacteric, showed maximum expression at the onset of climacteric peak on day 8 (Figure S6c) and then declined gradually during days 11-15 after harvest.

We have further investigated the effect of exogenously applied ethylene on the pattern of changes in the transcript levels of *MA-MADS5* during ripening. In pulp, as compared to normal ripening, transcript abundance of *MA-MADS5* was found to increase substantially at the preclimacteric stage after ethylene treatment. Expression of *MA-MADS5* was also increased at the climacteric and postclimacteric phases following ethylene treatment (Figure S6d and e). On the other hand, in peel, *MA-MADS5* expression did not increase significantly even after ethylene treatment as compared to fruit ripened naturally (Figure S6f and g).

Establishment and characterization of affinity purified polyclonal antibody raised against MA-MADS5

We have generated a polyclonal antibody against the purified partial recombinant MA-MADS5 protein as described under “Materials and methods”. We have used the C-terminal part of MA-MADS5 protein, which corresponds to C-terminal 170 amino acids, for antibody production. This C-terminal part of MA-MADS5 protein showed 14.7% amino acid sequence homology with MA-MADS1, 15.3% MA-MADS2, 30% with MA-MADS3, 15.9% with MA-MADS4, 94.7%

with MA-MADS5 and 10% with MA-MADS6 respectively. The affinity resin purified anti-MA-MADS5 antibody reacted specifically with the purified full length and partial recombinant MA-MADS5 proteins in an ELISA (data not shown) and with the 27-kDa MA-MADS5 protein in nuclear extract in Western bolt analysis (Figure S7a). The antibody was also found to specifically recognize the full length and other deleted versions of recombinant MA-MADS5 proteins (including fragments 'D', 'E', 'F' and 'G') while unable to detect the 'B' and 'C' fragments (Figure S7b). Preimmune serum was used as control which did not show any cross reactivity with MA-MADS5 protein (both full length and deleted versions) (data not shown). Regarding the specificity of cross recognition of anti-MA-MADS5 antibody, we have also validated the possibility of cross reactivity of the antibody with the other MA-MADS proteins reported from banana in previous study [13]. *MA-MADS1-3* have been found to be expressed in fruit tissues at their highest levels while *MA-MADS4*, *MA-MADS5* and *MA-MADS6* are shown to be expressed at higher levels in other tissues. To test whether the antibody cross reacts with other family members of MADS-domain proteins (via the more weakly conserved K domain, which was included in the overexpression construct), we selected MA-MADS2 protein, which has been shown to be highly expressed in banana fruit during ripening [13]. 6X-His tagged recombinant MA-MADS2 was overexpressed in *E coli* and affinity resin purified protein was immunoprobed with anti-MA-MADS5 polyclonal antibody in Western blotting. Such analysis revealed no cross reactivity of MA-MADS2 with the antibody (Figure S7c). We have also tested the relative cross reactivity of anti-MA-MADS5 antibody with recombinant MA-MADS1 and MA-MADS3 proteins since MA-MADS1 and MA-MADS3 are found to be highly expressed in banana fruit. The affinity purified anti-MA-MADS5 antibody did not cross react to recombinant MA-MADS1 or MA-MADS3 protein in protein gel blot analyses (data not shown). On the other hand, since MA-MADS5 showed high degree of amino acid sequence similarity and close phylogenetic similarity with MA-MADS5, we considered MA-MADS5 as the similar representative homologue of MA-MADS5 in Giant Governor and therefore did not consider to test the cross reactivity of the antibody with recombinant MA-MADS5 separately. Furthermore, the anti-MA-MADS5 polyclonal antibody was found to specifically immunoprecipitate the full length and other deleted versions of MA-MADS5 proteins (Figure S7d). Taking these results together, we confirm that this antibody is highly specific for MA-MADS5 protein and can be effectively used

to determine the accumulation level and sub-cellular localization of MA-MADS5 protein in banana fruit and other tissues.

Synthetic MADS box binding element (CArG) from banana *SPS* promoter regulates tissue specific gene expression in transgenic tobacco plants

To study whether the MA-MADS5 binding element, the CArG-box sequence, is functionally active in regulating gene expression, we prepared a synthetic promoter fragment containing tandem repeats of triple CArG-box sequence, identified from *MA-SPS* promoter (3XMADS box binding element or *3X MDBE*), in fusion with *GUS* reporter gene. A similar *3X MDBE-GUS* construct was prepared carrying mutated version of CArG-box element ($3X\ MDBE_m$) as control (Figure S9a). Since it was difficult to get reproducible results in transient expression system in banana, we introduced the promoter-reporter constructs individually into tobacco plants by leaf-disc transformation method to generate stable transgenic plants. Several homozygous lines for each transgene were generated to study promoter activity. Four independent transgenic lines were tested for each construct by genomic PCR to detect the integration of the construct (Figure S9c). To investigate the activity of *3XMDBE* containing synthetic promoter, we carried out RT-PCR to examine *GUS* expression in leaves of transgenic tobacco lines for each transgene. As shown in Figure S9d and e, *GUS* expression and GUS activity could be detected in all the four independent transgenic lines carrying *3XMDBE-GUS* transgene while no *GUS* mRNA was detected for $3X\ MDBE_m$-*GUS* transgene (data not shown). Together these results indicate the activity of the synthetic promoter carrying *3X MDBE* of banana in directing gene expression.

To further examine whether *3X MDBE* directs tissue specific gene expression, we examined endogenous message levels of *GUS* by RT-PCR in different tissues of transgenic tobacco lines carrying *3X MDBE-GUS* transgene. There was hardly any *GUS* expression in root and stem, while *GUS* expression was detected in leaf, flower and fruit tissues (Figure S9f, upper panel). Analysis of GUS activity in the indicated tissues of transgenic tobacco lines showed similar patterns of transgene activity as observed in case of *GUS mRNA* expression profile in various tissues of transgenic tobacco plants (Figure S9g). On the other hand no *GUS* expression was detected in any tissues of transgenic tobacco lines carrying $3X\ MDBE_m$-*GUS* construct (Figure S9f, lower panel). Together, these results have indicated that the CArG-box motif (MADS-box

binding element) of banana *SPS* gene promoter is functionally active in controlling tissue specific gene expression.
